# Multi-component self-assembled molecular-electronic films: towards new high-performance thermoelectric systems†

**DOI:** 10.1039/d2sc00078d

**Published:** 2022-04-15

**Authors:** Troy L. R. Bennett, Majed Alshammari, Sophie Au-Yong, Ahmad Almutlg, Xintai Wang, Luke A. Wilkinson, Tim Albrecht, Samuel P. Jarvis, Lesley F. Cohen, Ali Ismael, Colin J. Lambert, Benjamin J. Robinson, Nicholas J. Long

**Affiliations:** Department of Chemistry, Imperial College London, MSRH White City London W12 0BZ UK n.long@imperial.ac.uk; Physics Department, Lancaster University Lancaster LA1 4YB UK; Department of Physics, College of Science, Jouf University Skaka Saudi Arabia; Department of Mathematics, College of Science, Qassim University Almethnab Saudi Arabia; The Blackett Laboratory, Imperial College London, South Kensington Campus London SW7 2AZ UK; Department of Chemistry, University of York Heslington York YO10 5DD UK; Department of Chemistry, Birmingham University Edgbaston Birmingham B15 2TT UK; Department of Physics, College of Education for Pure Science, Tikrit University Tikrit Iraq

## Abstract

The thermoelectric properties of parallel arrays of organic molecules on a surface offer the potential for large-area, flexible, solution processed, energy harvesting thin-films, whose room-temperature transport properties are controlled by quantum interference (QI). Recently, it has been demonstrated that constructive QI (CQI) can be translated from single molecules to self-assembled monolayers (SAMs), boosting both electrical conductivities and Seebeck coefficients. However, these CQI-enhanced systems are limited by rigid coupling of the component molecules to metallic electrodes, preventing the introduction of additional layers which would be advantageous for their further development. These rigid couplings also limit our ability to suppress the transport of phonons through these systems, which could act to boost their thermoelectric output, without comprising on their impressive electronic features. Here, through a combined experimental and theoretical study, we show that cross-plane thermoelectricity in SAMs can be enhanced by incorporating extra molecular layers. We utilize a bottom-up approach to assemble multi-component thin-films that combine a rigid, highly conductive ‘sticky’-linker, formed from alkynyl-functionalised anthracenes, and a ‘slippery’-linker consisting of a functionalized metalloporphyrin. Starting from an anthracene-based SAM, we demonstrate that subsequent addition of either a porphyrin layer or a graphene layer increases the Seebeck coefficient, and addition of both porphyrin and graphene leads to a further boost in their Seebeck coefficients. This demonstration of Seebeck-enhanced multi-component SAMs is the first of its kind and presents a new strategy towards the design of thin-film thermoelectric materials.

## Abstract 2

Through an experimental and theoretical study, cross-plane thermoelectricity in Self-Assembled Monolayers (SAMs) was enhanced by adding extra molecular layers, presenting a new strategy towards the design of high thermoelectric materials.

## Graphical abstract

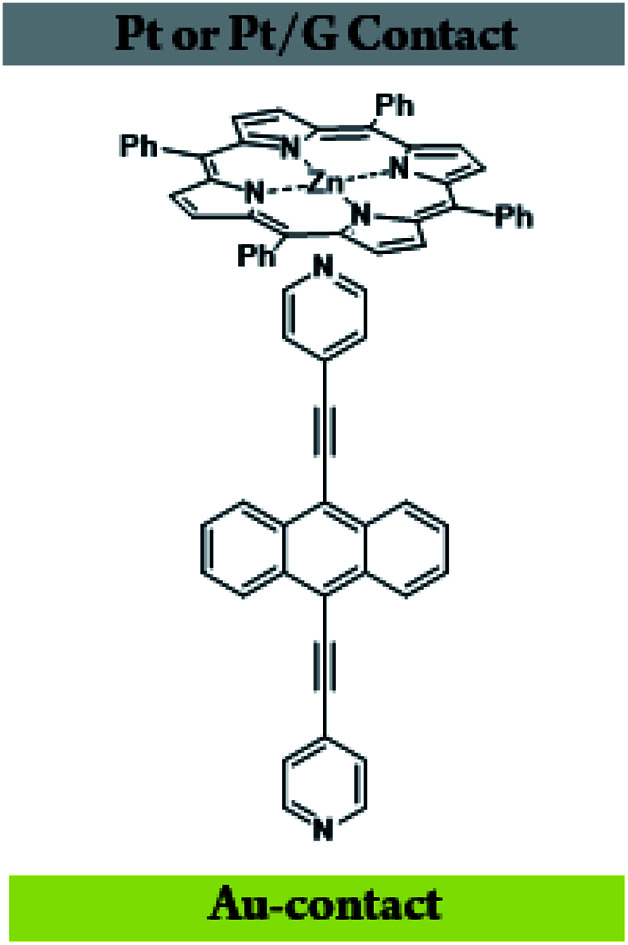

## Introduction

**Fig. 1:**
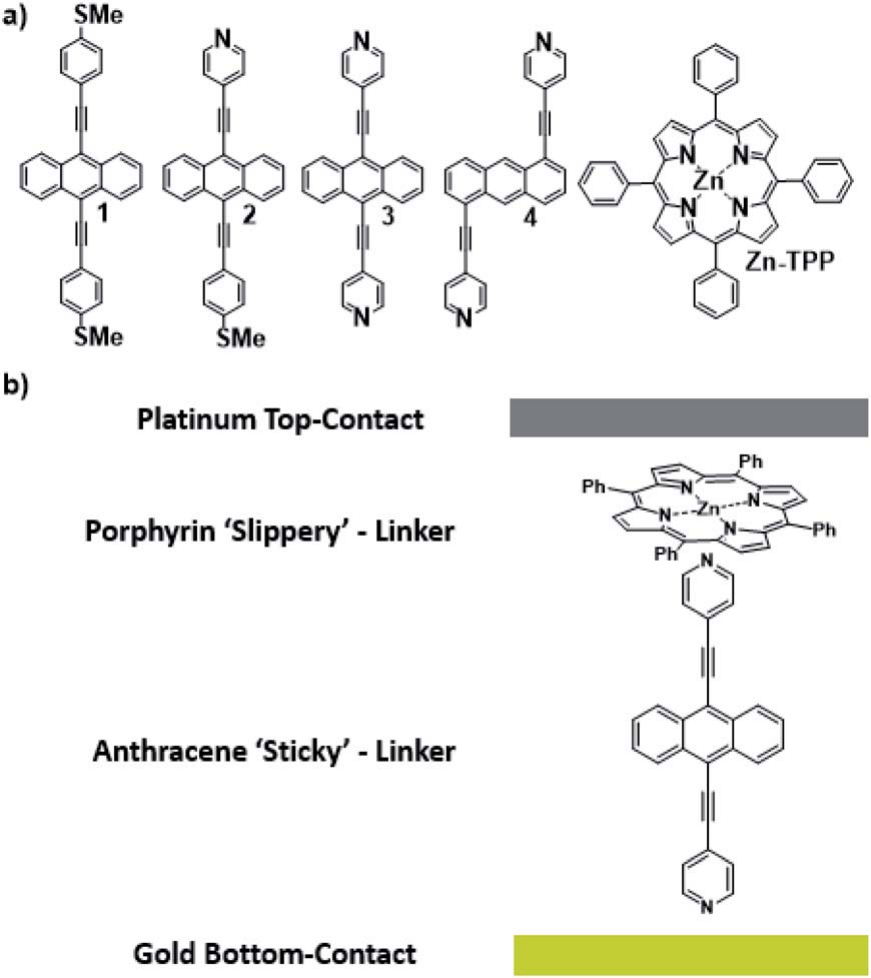
(a) Chemical structures of studied molecules; (b) typical schematic of a fabricated junction.

**Fig. 2:**
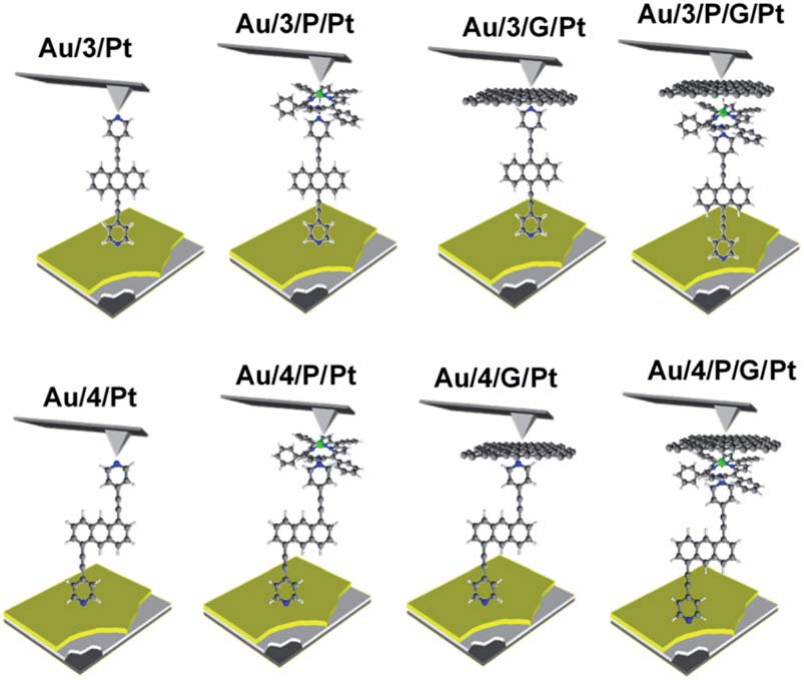
A schematic representation of the junctions fabricated, with junction ‘names’ nomenclature. Within this nomenclature Au represents a gold-bottom contact, 3 and 4 denote a monolayer of either molecule 3 or 4 respectively, P represents a porphyrin monolayer, G represents graphene and Pt represents a platinum-top contact to form Au/3/Pt, Au/4/Pt, Au/3/P/Pt, Au/4/P/Pt, Au/3/G/Pt, Au/4/G/Pt, Au/3/P/G/Pt, Au/4/P/G/Pt respectively.

Waste heat is generally regarded as a low quality form of energy, which unlike electricity, is relatively difficult to transform and utilize.^1^ With about 70% of the world's power usage currently going to waste in the form of heat, the development of new techniques to recapture this energy are of high importance.^2,3^ Organic materials have been widely studied for their ability to convert heat into electricity *via* the Seebeck effect, with notable performance presented by polymers of thiophenes, perylene diimides and beyond.^4–6^ However, this focus on polymer chemistry often leads to ill-defined surface structures, which complicates the fundamental understanding of their thermoelectric behavior. Single-molecule junctions present an interesting alternative for thermoelectric systems and a wealth of research has been directed towards the study of charge transport in single-molecules and their ensembles.^8–11^ These systems have very well-defined, highly modifiable structures that can provide (i) tunability of the sign of the Seebeck coefficient of the junction, (ii) access to powerful molecular features such as quantum interference (QI), which has been shown to boost junction Seebeck coefficients, and (iii) redox chemistry.^12–25^ A pedagogical account of QI effects in molecules can be found in [*Quantum Transport in Nanostructures and Molecules* C. J. Lambert, IOP Publishing, 2021].^26^ Recent developments in the implementation of molecular-junction techniques and self-assembled monolayers have shown that high quality monolayers can be formed between symmetric^27,28^ or asymmetric electrodes^29^ where the SAM design facilitates assembly of the top electrode.^30,31^ Furthermore, single molecule effects, such as tunability and QI enhancement,^32^ can be translated to large area parallel arrays of molecules, opening the door for the development of new *de novo* strategies for the fabrication of thermoelectric devices from discrete molecules.^33–36^ However, these designs are often hindered by their strong chemical binding between molecules and metallic electrodes, which can restrict their tunability. For example, the thiol–gold bond has a binding energy of more than 1 eV, which promotes mechanical robustness, but at the same time induces mechanical stiffness which controls phonon transport across the interface and cannot be tuned. Rigid-contacts also diminish the efficacy of these designs in thermoelectric applications, where the decoupling of electronic and thermal contributions presents an exciting avenue in the fabrication of systems with high-values for the thermoelectric figure of merit (*ZT*).^37–40^ Within this study, we aimed to overcome this limitation by investigating new strategies towards the formation of thermoelectrically efficient devices containing highly asymmetric electrode-anchoring groups, which realize, for the first time, QI enhancement of the thermoelectric properties of multi-layered films. To achieve this, we aimed to assemble a system containing a highly conductive, and rigid ‘sticky’ linker, that displays strong features of constructive quantum interference (CQI) and bind this to a gold electrode at one-end. The other end was then bound to either a platinum electrode or a graphene-coated platinum electrode, through the use of a ‘slippery’ linker with an extended π-system, whose propensity to π-stack with graphene should facilitate high-conductance, whilst limiting thermal propagation associated with phonon transport (due to its non-covalent interaction). The ability to create stacked multi-component assemblies, whilst preserving CQI, is highly desirable. It has previously been demonstrated that the Seebeck coefficient of two C60 molecules stacked on top of each other is significantly higher than that of a single C60 and we were keen to investigate whether a similar effect could be translated into asymmetric CQI-enhanced multi-component films.^24^ An asymmetric unimolecular system is synthetically challenging and would also be unlikely to form well-ordered molecular films. Here we overcome this challenge through the use of multi-component self-assembly. We fabricated multi-component systems composed of, first, rigid molecular wires based around a central anthracene-core (1–4, Fig. 1(a)) and secondly, a Zn-TPP (zinc-tetraphenylporphyrin) ‘slippery’ linker. The aim being to couple the metal centre of the Zn-TPP to the exposed terminal group of the assembled wire (Fig. 1(b)). These wires differ in the nature of their terminal groups, which contain thiomethyl–thiomethyl (1), thiomethyl–pyridyl (2) and pyridyl–pyridyl (3,4) termini, as well as in their connectivity around the anthracene molecular core *i.e.* a 9,10-substitution pattern around the anthracene core (in 1–3) *vs.* 1,5-substitution (in 4). These molecules were chosen, because they can be assembled to form well-ordered molecular films that demonstrate strong features of CQI at room-temperature.^15,16,33,41^ Solution-based NMR experiments were used to initially evaluate the propensity of anthracenes (1–3) to bind to Zn-TPP. After observing that the dipyridyl species (3) bound with the highest efficiency, we assembled 3 and 4 into thin-film materials and subsequently deposited Zn-TPP on-top. The thermoelectric properties of SAMs of 3 and 4 were then evaluated, experimentally and theoretically, with and without the inclusion of a porphyrin cap, and utilizing different platinum top-contacts. Eight different configurations were assessed, as shown in Fig. 2, in order to evaluate our hypothesis of increased thermoelectric efficiency.

## Results and discussion

### Synthesis

For details relating to the synthesis of molecules 1–3, refer to our recent publications.^33,34^4 was synthesized through the use of a Sonogashira cross-coupling reaction between synthesized 1,5-dibromoanthracene and commercially available 4-ethynylpyridine hydrochloride. Following this, flash-chromatography was carried out using an alumina grade V column to produce molecule 4 in high yield. This was fully characterized through the use of both ^1^H-NMR, ^13^C{^1^H}-NMR spectroscopy, as well as HR-MS (see ESI†).

### Coordination studies

**Fig. 3:**
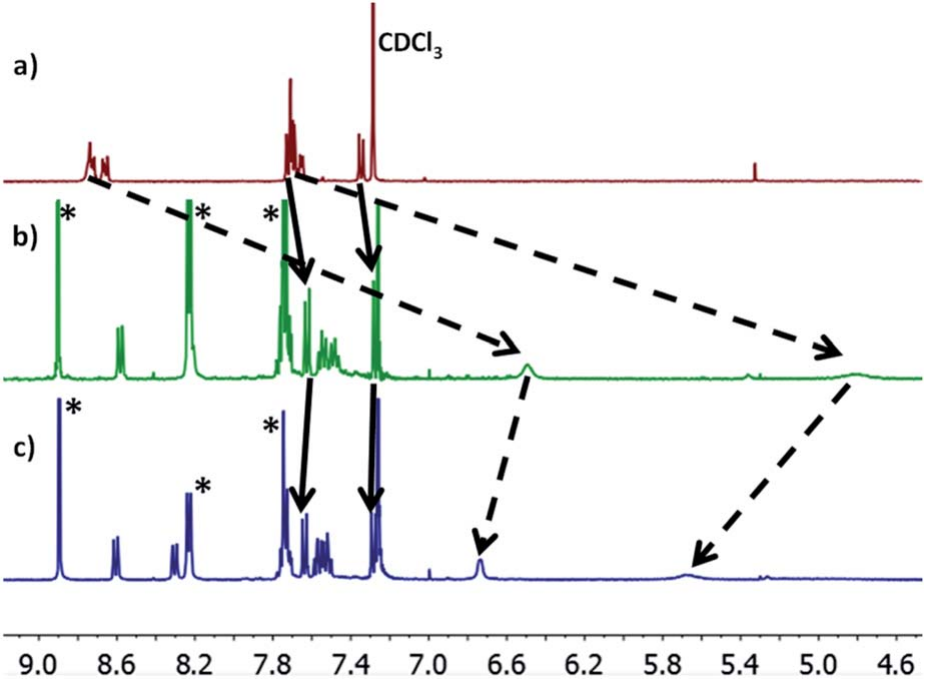
(a) ^1^H-NMR spectra of 2; (b) a 1 : 1 mixture of molecule 2 : Zn-TPP; (c) a 2 : 1 mixture of 2 : Zn-TPP. Movement of peaks associated with thioanisole and pyridine protons are indicated with solid and dashed lines respectively. Porphyrin associated peaks are labelled with (*).

Molecules 1–3 contain three different sets of anchor groups (thiomethyl/thiomethyl, thiomethyl/pyridyl and pyridyl/pyridyl). Prior to self-assembly we used solution-based ^1^H-NMR experiments to evaluate the ability of these groups to bind to Zn-TPP. To achieve this, Zn-TPP was dissolved in CDCl_3_ and then added to either 1 or 2 equivalents of molecules 1–3 to create six different solutions, whose ^1^H-NMR spectra could then be examined to give a qualitative indication of coordination. Focusing first on 1, no change was observed in either the shape or position of the peaks upon combination with Zn-TPP. In contrast, on examining 2, terminated with one thioanisole and one pyridyl unit, the ^1^H-NMR spectrum presented a number of interesting changes upon addition of Zn-TPP. When combined in a 1 : 1 ratio, there was a clear and distinct shift in the peak-positions of all the anthracenyl protons. It can be seen that the protons in the α and β positions, relative to the nitrogen atom were shifted upfield by >2.0 ppm, with both of the peaks displaying a large degree of broadening (generally associated with fluxionality between two states on a faster timescale than the spectrometer can measure). In contrast to this, the protons on the methyl group, as well as those α and β to the thiomethyl moiety exhibited a more modest shift of approximately 0.07 ppm (Fig. 3). This indicates that molecule 2 binds to the porphyrin through its nitrogen atom, with peaks attributable to thioanisole associated protons shifting only slightly, as a result of porphyrin coordination at the other end of the molecule. Further evidence for this asymmetric binding can be seen when examining the protons associated with the anthracene core. For 2, the four interior anthracenyl protons exhibit a poorly defined multiplet centered at 8.66 ppm, which upon addition of 1 equivalent of porphyrin, splits into two doublets of doublets, centered at 8.58 and 8.22 ppm respectively (Fig. 3). These changes are likely caused by an association of the anthracene molecule with strong porphyrin-based ring currents, ‘shielding’ the anthracene protons and causing upfield shifts that are relative to the protons proximity to the bound porphyrin. Subsequently examining the 2 : 1 mixture (Fig. 3c), we observed a slight downfield shift of the anthracenyl protons, though still maintaining lower frequencies than that of molecule 2 when examined on its own. This likely results from molecule 2 coordinating axially on both sides of Zn-TPP, thereby ‘sharing’ any porphyrin associated ring-currents across two molecules rather than one and producing a less-distinct shift. These trends continued through our analysis of 3 with a 1 : 1 ratio of 3 : Zn-TPP generating large upfield shifts for all anthracene associated protons, with the peaks then subsequently moving slightly downfield upon examination of the 2 : 1 mixture. We utilized DOSY (Diffusion-Ordered Spectroscopy) NMR to provide further confirmation that molecules 2 and 3 coordinate to Zn-TPP in solution. When examining the 1 : 1 mixture of 2 : Zn-TPP, all anthracene- and porphyrin-associated protons generated a diffusion coefficient of approximately 4.75 × 10^−6^ m^2^ s^−1^, providing clear evidence for association of the two systems. This behavior was consistent through all of the samples. In the case of 3, multiple distinct species were observed in the DOSY analysis, with sequentially lower diffusion coefficients. This indicated to us the formation of a number of increasingly sized oligomers, similar to those previously reported in other dipyridyl/porphyrin assemblies.^42,43^ Finally, we attempted to obtain evidence of discrete anthracene–porphyrin structures through the use of HR-MS. Only the samples generated from molecule 3 showed a molecular ion peak that could be assigned to a porphyrin–anthracene complex. This may be an indication of increased bond strength but is more likely a result of molecule 3 containing two porphyrin binding groups as opposed to one, making it statistically more likely for a molecular ion to be observed in both cases. Full details relating to these coordination experiments can be found in the ESI (Section 1.4–1.6). With these results in mind, multi-component assembly and thermoelectric characterization were focused on our dipyridyl species (3), as well as a second dipyridyl (4) which has previously been shown to display similar coordinative behavior, with other species (1, 2) acting as reference systems to the measurements.^44,45^

### SAM formation

Self-assembled monolayers (SAMs) of 1–4 were prepared by standard methods on a template-stripped gold substrate (Au^TS^), with details described in the ESI.†^46,47^ The SAMs covered gold was further modified by immersing in a 1 mM Zn-TPP solution for 20 min. During all growth procedures, a gold quartz crystal microbalance (QCM) substrate was grown together with the Au^TS^, and measured by a QCM system to quantify the amount of molecule on the substrate (see ESI,† Section 3.2). QCM indicated predominantly monolayer Zn-TPP growth on SAMs of Au/3 and Au/4 with *ca.* 10% and 20% of the Zn-TPP film comprising multilayers (see Fig. S57 in ESI†). The quality of SAMs was characterized by XPS, AFM imaging and nano scratching methods.^48,49^

### X-ray photoelectron spectroscopy

**Fig. 4:**
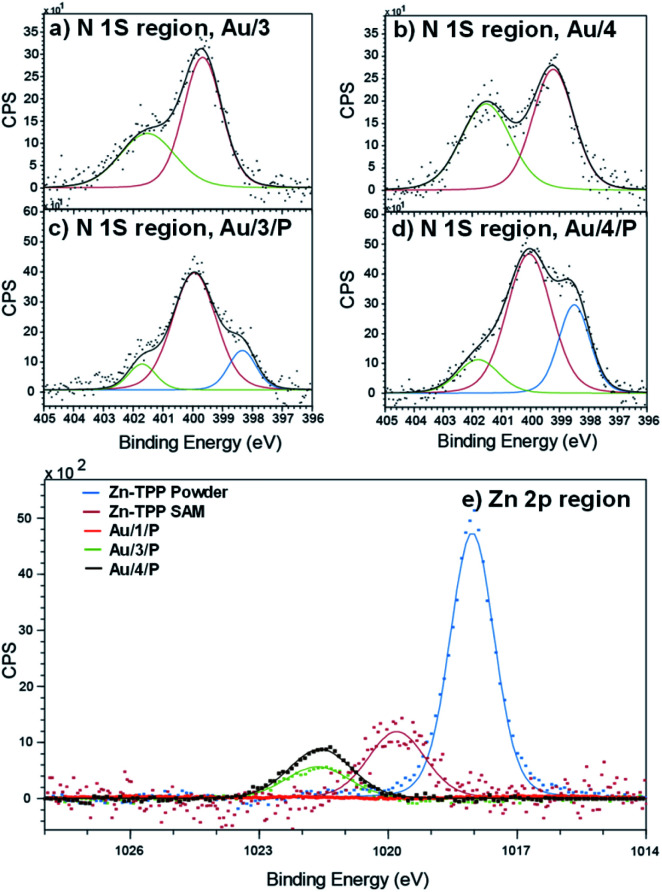
XPS characterization of surface binding for SAM and Zn-TPP layers. Fitted spectra in the N 1s region for (a) Au/3 (b) Au/4, (c) Au/3/P and (d) Au/4/P. The blue curve in (c) and (d) corresponds to the nitrogen atoms present within Zn-TPP. (e) Zn 2p spectra of Au/1/P, Au/3/P and Au/4/P referenced against a Zn-TPP SAM and the Zn-TPP molecular powder. Significant shifts in binding energy support the assignment of Zn-TPP binding to pyridyl groups.

To demonstrate the binding of Zn-TPP to the terminal pyridyls, the assembly and structure of SAMs featuring 1–4, pre- and post-exposure to Zn-TPP, were examined by XPS. A spot size of 350 μm × 700 μm was chosen to avoid artifacts from small multilayer clusters of molecules or effects from underlying Au^TS^ domains. Measurements in the N 1s region for Au/3 and Au/4 are shown in Fig. 4(a) and (b). These spectra revealed identically positioned peaks at 399.7 eV and 401.5 eV corresponding to bound and unbound pyridine groups, respectively, confirming an ordered monolayer.^50–53^ Spectra collected for Au/3/P and Au/4/P (post exposure to Zn-TPP) exhibit an additional peak in the N 1s region located at 398.3 eV and 398.5 eV respectively, corresponding to the nitrogen atoms present within Zn-TPP.^54–56^ A small additional N 1s peak is expected for the N–Zn interaction, however, this is not observed because of the dominating contribution of the N–Au interaction at the same energy.^57^ We note that in all cases the bound N 1s contribution has a smaller peak area than the unbound, particularly for the Zn-TPP capped samples, reflecting the increased attenuation expected for a nitrogen atom located ∼2 nm below the surface layer. XPS of the Zn 2p region was further used to verify Zn-TPP layer attachment. As shown in Fig. 4(e), distinct Zn 2p_3/2_ peaks located at 1021.6 eV were observed for Au/3/P and Au/4/P. In comparison, no such Zn 2p_3/2_ signal was found for Au/1, post exposure to Zn-TPP, confirming that pyridine is required to stabilize the Zn-TPP layer. In the Au/2 system, we note that there is a mixture of both bound and unbound nitrogen and thiomethyl units suggesting that anthracene 2 forms a mixed layer with almost equal probability of either anchor group binding to the Au^TS^ surface. Au/2 is discussed in more detail in ESI Section 4.† In Fig. 4(e) we include data for a Zn-TPP SAM (1019.8 eV) as well as pure Zn-TPP powder (1018.1 eV) to act as a reference. In each case a significant shift in energy is observed, suggesting strong pyridyl coupling to the porphyrin, in agreement with our ^1^H-NMR experiments. Characterization of the thiomethyl groups in Au/1 revealed two sets of doublets, characteristic of a thiomethyl group bound and unbound to gold respectively, as shown in ESI Fig. S66.† Peaks were fitted using two Gaussian–Lorentzian S 2p doublets with a 2 : 1 area ratio and splitting of 1.2 eV.^58^ The bound thiomethyl was evidenced by a doublet at 161.9 eV (S 2p_3/2_) and 163.1 eV (S 2p_1/2_), characteristic of the sulfur binding energy of a gold–thiomethyl bond. Furthermore, an additional sulfur doublet at 162.9 and 164.17 eV is present, typical of an unbound thiomethyl group.

### AFM characterisation

**Fig. 5:**
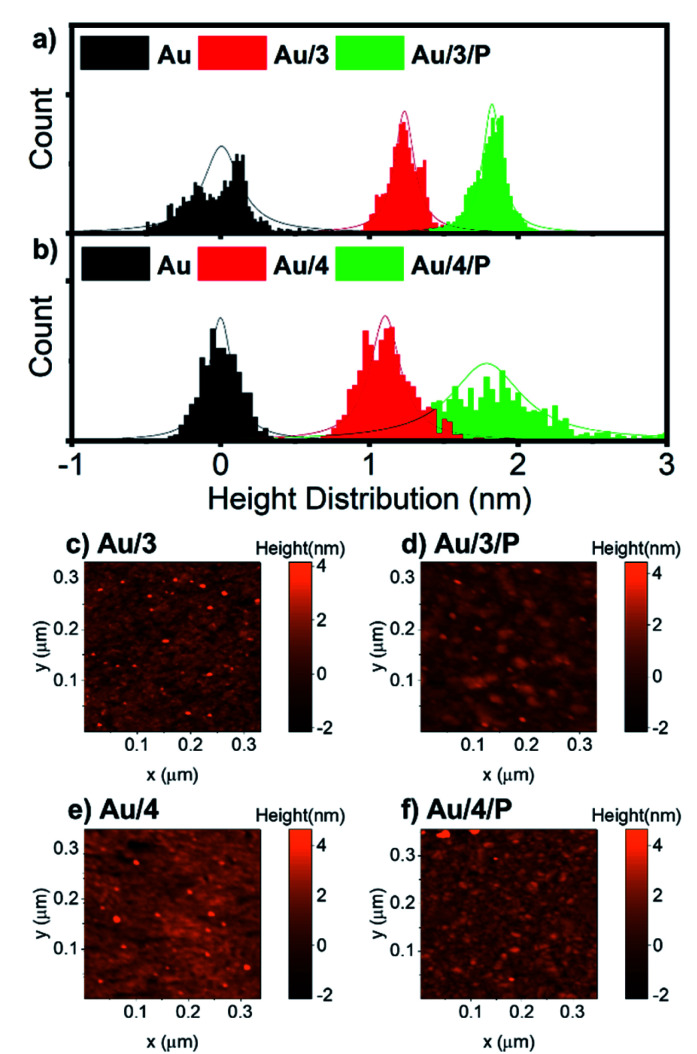
(a) Height distribution relative to Au^TS^ of Au/3 and Au/3/P, and (b) Au/4 and Au/4/P derived from SPM nano-scratching. (c–f) AFM topographical image of Au/3, Au/3/P, Au/4 and Au4/P, scan size 320 nm × 320 nm.

For all Au^TS^ samples, we observed polycrystalline films comprising regions of widely differing single-orientation domain sizes consistent with previous studies of the nature of Au^TS^ substrates.^59–61^ Only Au^TS^ samples with RMS roughness below 0.2 nm were used for SAM growth. The height distributions of Au/3 and Au/3/P, and Au/4 and Au/4/P were obtained from nano-scratching, as shown in Fig. 5(a and b), with the peak-to-peak distance between gold and SAMs indicating the SAMs thickness.^62^ The thickness of Au/3 was measured to be 1.2 nm, which suggests that the molecules are standing upright on the Au surface with an average tilt angle of 47°, by considering that the molecular length of 3 is about 1.9 nm. The thickness of Au/3/P was 1.8 nm, resulting in the height contribution from the Zn-TPP layer being 0.6 nm. This value is comparable with a Zn-TPP molecule lying flat on a Au surface (∼0.45 nm).^63^Au/4 and Au/4/P were found to be comparable with thicknesses of 1.1 and 1.7 nm respectively, resulting in an average tilt angle of 50° and a Zn-TPP layer of 0.6 nm. Small scale (320 nm) topographical images of Au/3, Au/3/P, Au/4 and Au/4/P are shown in Fig. 5(c–f) where the change in film feature resolution is attributed to binding of the planar, slippery Zn-TPP linker. Further examples of SAM growth of 3 and 4 can be found in Fig. S58, of the ESI.† Brighter features in Fig. 5(d) and (f) correspond to multilayer regions of Zn-TPP consistent with the measurements by QCM (Fig. S57†) and correspond to the broader height distribution of Au/4/P of Fig. 5(b). Comparable assembly and AFM characterisation was performed on Au/1 and Au/2, however in these cases, AFM images suggested that there was little interaction between Zn-TPP and the underlying SAM in agreement with our XPS (see ESI, Section 4†). It appears that, in these cases, the Zn-TPP deposition produced clusters of porphyrin aggregates on the surface, rather than coordinative binding to the molecular anchors.

### Electrical and thermoelectrical measurements

**Fig. 6:**
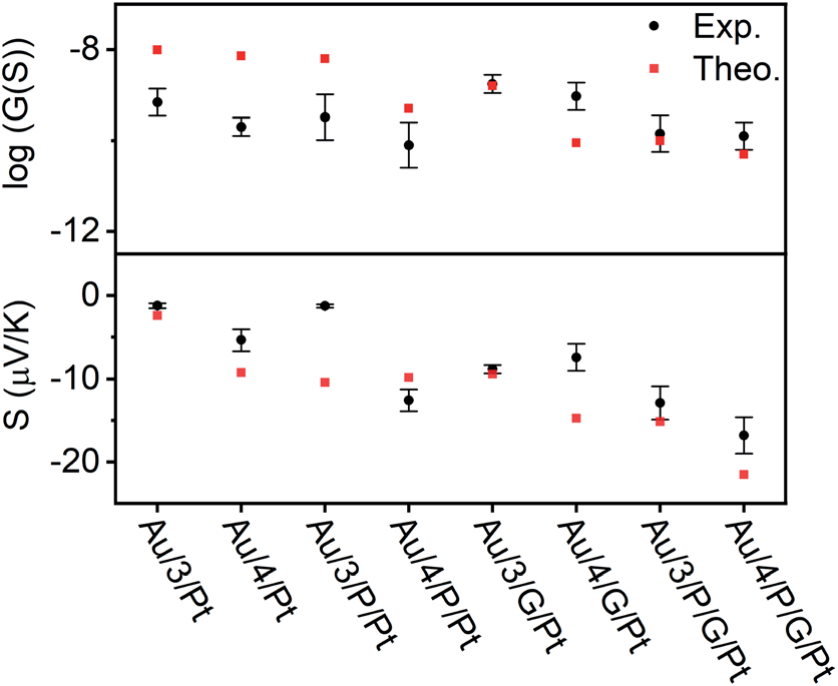
Electrical and thermoelectrical properties of the eight junctions. A comparison between experiment and theory.

A modified conductive probe AFM system with an integrated heater stage, previously reported, was used for molecular conductance and Seebeck characterization of the 8 assembled junctions illustrated in Fig. 2 (Experimental details in ESI Section 3†) and the results of these measurements are shown in Fig. 6.^33,34,64^ In all cases, a Au^TS^ substrate acted as the source, and either a platinum or graphene-coated platinum conductive probe, acted as the drain. The conductance (*G*) ratio between the anthracene SAMs (Au/3 and Au/4) and their equivalent porphyrin-coupled structures (Au/3/P and Au/4/P) was interpreted through the use of the following equation linking the internal and interfacial resistances (*R*) within the junction:
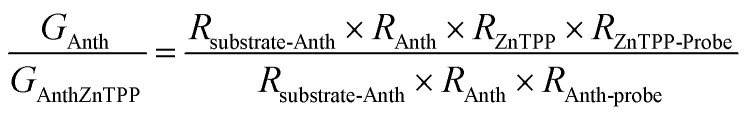


In all cases, this ratio was higher than 1, as expected due to the additional resistance in the junction arising from *R*_ZnTPP_, which has the effect of decreasing the conductance of the uncoupled SAMs with respect to their coupled counterparts. This ratio was expected to be lowered by using a graphene-coated probe instead of a Pt probe, due to the π-stacking that occurs between Zn-TPP and the graphene-coated probe, which decreases the interfacial resistance *R*_ZnTPP-Probe_. Conversely, the ratio increased, which we attributed to the higher binding energy associated with the graphene (see Density functional theory section below). The Seebeck measurement was obtained from a linear fit of thermal voltage, *V*_Th_, *vs.* temperature difference between sample and probe, Δ*T*, at 4 different temperatures (ESI Section 4.4†). It has been previously shown that the probe can be assumed to be at room temperature due to its high thermal conductivity (Si is ∼150 W mK^−1^) and contact with a large thermal reservoir, in this case the probe holder and body of the microscope.^66^ When comparing the uncoupled SAMs with their porphyrin coupled equivalents, this experiment suggested a 50% to 100% enhancement of the Seebeck coefficient can be achieved. Previous studies on comparable AFM systems have shown that through-air conductance of heat from the sample to the probe is negligible and that at least 95% of the temperature difference occurs across the molecules in the junctions.^67,68^ Therefore, the increase in Seebeck coefficient seen in the Zn-TPP capped systems are due to the molecules in the junction and not the increased probe-substrate separation.

### Density functional theory

The transport properties of the eight junctions were further investigated using a combination of density functional theory and quantum transport theory to obtain the transmission coefficient *T*(*E*), describing electrons of energy *E* passing from the source to the drain electrodes.^69^ From this, the room-temperature electrical conductance *G* and Seebeck coefficient *S* were determined using eqn (S5), (S4) and Fig. S49–S56, of the ESI.† Fig. S45(B2 and B4)† demonstrates that a pyridyl-terminated anthracene binds 10 times more strongly to porphyrin (Zn-TPP) than a thioanisole-terminated anthracene (with binding energies of approximately 0.5 and 0.05 eV respectively) in agreement with the physical characterization described above. Fig. 6 (lower panel) shows the computed room-temperature Seebeck coefficients of the 8 different molecular structures given in Fig. 2. Previous comparisons^38^ between experiment and theory revealed that electron transport through poly-aromatic hydrocarbons takes place near the middle of the energy gap between the highest occupied molecular orbital (HOMO) and the lowest unoccupied molecular orbital (LUMO), and indeed we find that the closest agreement between theory and experiment is obtained for a Fermi energy near the mid-gap, as indicated by the vertical dashed lines in Fig. S49–S52.†^41^ As expected from literature studies of single molecules, electron transport through the eight junctions is LUMO dominated (due to the presence of pyridyl anchors), leading to the negative sign of the Seebeck coefficient for the 8 junctions, as shown in the lower panel of Fig. 6. As shown in Fig. 6, both experiment and theory reveal that addition of Zn-TPP tends to decrease the conductance of the Au/3/Pt and Au/4/Pt SAMs whilst increasing the magnitude of their Seebeck coefficients. Furthermore, the conductance's of SAMs formed from 3 are generally higher than those formed from 4, reflecting the higher degree of CQI in the former. Experimentally, the conductance's of Au/3/P/G/Pt and Au/4/P/G/Pt were measured to be close to those of Au/3/P/Pt and Au/4/P/Pt respectively and similarly, the conductance's of Au/3/G/Pt and Au/4/G/Pt were measured to be close to those of Au/3/Pt and Au/4/Pt respectively, revealing that inclusion of the graphene layer had a negligible effect on electrical conductance. In contrast, theory reveals that if the fraction of molecules making contact with the electrodes is unchanged by the inclusion of the graphene layer, then inclusion of the latter would be expected to decrease the conductance. This suggests that the higher binding energy associated with the graphene layer increases the fraction of molecules making contact with the top electrode. The experimental observation that inclusion of graphene barely affects electrical conductance is of interest, because it may present an opportunity for independently tuning thermal conductance by varying the phonon mismatch across the electrode-molecule boundary.

## Conclusions

We have utilized multi-layered self-assembly and asymmetric design to boost the Seebeck coefficients of self-assembled monolayers. Solution-based NMR experiments were used to successfully predict the ability of a series of anthracene-based molecular wires to bind to a porphyrin. Through this binding, SAMs of these molecules were able to stabilize addition of a porphyrin layer to their top face. These SAMs were characterized extensively through the use of AFM and XPS both with and without the inclusion of Zn-TPP, confirming the discrete structure of these systems and demonstrating a clear translation between the binding behavior of the molecules in solution and at the mesoscopic scale on a surface. Fig. 6 demonstrates that when starting from an anthracene-based SAM formed from 3 or 4, subsequent addition of either a porphyrin layer or a graphene layer can act to significantly increase their Seebeck coefficients, and addition of both porphyrin and graphene can lead to further increases, though this is dependent on the underlying SAM structure. Specifically, Fig. 6 demonstrates that a 50% to 100% enhancement of the Seebeck coefficient is achieved when comparing Au/4/Pt to Au/4/P/Pt, whose Seebeck coefficients are −5.4 μV K^−1^ and −12.6 μV K^−1^ respectively. However, as shown in Fig. 6, there is no such enhancement in the measurements when comparing Au/3/Pt to Au/3/P/Pt. We attribute this difference to the structural differences between the SAMs of 3 and 4, as shown by the QCM results. We observe the packing density for SAM 3 is an order of magnitude higher than SAM 4 (Fig. S57†), which would lead to stronger intermolecular interactions in SAM 3. Therefore, this difference in the Seebeck trends may be associated with pi–pi overlap between neighbouring molecules in SAM 3, which would shift the frontier molecular orbital energies relative to the Fermi energy of the electrodes and change the slope of the transmission function at the Fermi energy. For the future, one could envisage tuning the pi–pi overlap by introducing side groups, which reduce the packing density. However, such side groups would also affect the energies of frontier orbitals and therefore such a study may not yield an unambiguous answer. This work also reveals interesting information pertaining to the charge transport properties of these multi-component systems. In the Landauer theory of electron transport, electrons are assumed to pass through a molecule, without undergoing inelastic scattering, *i.e.* their energy is conserved and they are said to remain phase-coherent. In such a theory, the electrons lose coherence by undergoing inelastic scattering in the electrodes. Provided electrons undergo phase-coherent tunnelling as they pass through the molecule, the conductance should decay exponentially with length. On the other hand, as demonstrated previously, this trend ceases when transport becomes incoherent.^70^ For the anthracene molecules studied here, previous studies have shown that electrons passing through these molecules are phase-coherent.^33^ Our experiment and theory show that the effect of adding porphyrin always decreases the conductance, which suggests that 3/P and 4/P should be regarded as single phase-coherent entities. Similarly, the theoretical conductance of *X*/P/G is found to be lower than the theoretical conductance of *X*/P, (where *X* = 3 or 4), because theory assumes that transport takes place *via* phase-coherent tunnelling. In contrast, experiment shows that the effect of moving from Au/*X*/Pt to Au/*X*/G/Pt increases the conductance and from Au/4/P/Pt to Au/4/P/G/Pt yields almost no change in conductance, which suggests that the assumption of phase-coherent tunnelling is not correct, when the graphene (G) is added. Instead, these measurements suggest that the graphene should be regarded as part of the electrode and that inelastic scattering takes place within the graphene as well as the metallic part of the electrode. In other words, the length of the molecular component of the junction is determined by the *X*/P component and does not increases when the graphene is added. Theory predicts and experiment confirms that the Seebeck coefficient of *X*/P/G is higher in magnitude than that of *X*/P. However, the experimental value is slightly lower than the theoretical value, which again suggests that the G introduces inelastic scattering. Beyond introducing decoherence, the graphene may affect the conductance in other ways. For example, modifying the probe's radius of curvature due to the graphene imperfectly spanning any probe-asperities. A significant decrease in the radius of curvature would result in fewer molecules being contacted in the junction, which would cause the conductance of the junction to decrease, as is observed in the case of moving from Au/3/P/Pt to Au/3/P/G/Pt. We would also note that a number of these multi-component systems retain strong features associated with CQI, and finally, our experiments also show that this methodology can be applied to tailor each end of a molecular junction to different electrode materials. The fabrication of these novel ‘sticky’ to ‘slippery’ linker systems presents a significant breakthrough in the field of molecular electronics, overcoming the need for rigid contacts to metallic electrodes, a critical step in the design of future thin film devices. This study could also reasonably be extended to further work in tailoring different ends of a multicomponent system to different materials, as a route towards decoupling electronic and thermal contributions in the generation of thermopower. We are currently undertaking work to synthesize new ‘sticky’ and ‘slippery’ linkers and engineer more efficient devices utilizing this methodology.

## Methods

### Compound synthesis and characterisation

The synthesis of molecules (1–3) has previously been reported.^33,34^ All reactions were performed with the use of standard air-sensitive chemistry and Schlenk line techniques, under an atmosphere of nitrogen. No special precautions were taken to exclude air during any work-up. 1,5-Dibromoanthracene was synthesised through the use of an adapted literature procedure.^70^ All other reagents are commercially available and were used as received from suppliers, without further purification. Solvents used in reactions were collected from towers sparged with nitrogen and dried with 3 Å molecular sieves, apart from DIPA, which was distilled onto activated 3 Å molecular sieves under nitrogen. ^1^H- and ^13^C{^1^H}-NMR spectra were recorded on a Bruker Avance 400 MHz spectrometer and referenced to the residual solvent peaks of either CDCl_3_ at 7.26 and 77.2 ppm or CDCl_2_ at 5.32 or 54.0 ppm, respectively. ^1^H-NMR spectra were fully assigned using 2D correlation spectroscopy. Coupling constants are measured in Hz. Mass spectrometry analyses were conducted by Dr Lisa Haigh of the Mass Spectrometry Service, Imperial College London.

#### Preparation of molecule 4

1,5-Dibromoanthracene (0.20 g, 0.60 mmol), 4-ethynylpyridine hydrochloride (0.33 g, 2.38 mmol) and CuI (0.01 g, 0.06 mmol) were dissolved in toluene (100 mL) and DIPA (20 mL). The solution was degassed for 15 minutes and Pd(P-^*t*^Bu_3_)_2_ was added. The solution was then stirred at 50 °C for >16 hours. The solvent was removed *in vacuo* to give a black solid that was purified by flash chromatography on an alumina V column, eluting with 1 : 1 hexane : DCM to give the final product as a yellow solid (0.17 g, 0.45 mmol, 75%).

^1^H-NMR (CD_2_Cl_2_, 298 K, 400 MHz): *δ*_H_ = 9.02 (s, 2H, *H*11), 8.68 (dd, ^3^*J*_H–H_ = 4.4, ^4^*J*_H–H_ = 1.6 Hz, 4H *H*1), 8.21 (dd, ^3^*J*_H–H_ = 8.4, ^4^*J*_H–H_ = 0.8 Hz, 2H, *H*9), 7.90 (dd, ^3^*J*_H–H_ = 8.0, ^4^*J*_H–H_ = 1.2 Hz, 2H, *H*7), 7.61–7.55 (m, 6H, *H*2, *H*8) ppm; ^13^C{^1^H}-NMR (CDCl_3_, 298 K, 100 MHz): *δ*_C_ = 150.1 (Ar–C–H), 131.9 (Ar–C–H), 131.8 (Ar–C–C), 131.6 (Ar–C–C), 131.3 (Ar–C–C), 130.7 (Ar–C–H), 125.9 (Ar–C–H), 125.8 (Ar–C–H), 125.5 (Ar–C–H), 120.0 (Ar–C–H), 92.2 (–C≡C–), 92.2 (–C≡C–) ppm; MS ES+: calcd for C_28_H_17_N_2_ [M + H^+^] calcd 381.1392; found 381.1378.

### Coordination experiments

To evaluate the coordinative behaviour of the synthesised anthracene derivatives, we evaluated NMR data produced from both 1 : 1 and 2 : 1 ratios of anthracene : porphyrin. For each experiment 10 mg of anthracene was dissolved in 20 mL of DCM. Either 1 or 0.5 equivalents of Zn-TPP were added, to form 6 different samples, the solutions were then stirred for 1 hour before the solvent was removed *in vacuo* to give a purple solid that was analysed. Characterisation data of molecules 1–3 is included for the sake of comparison (see ESI Section 1.3–1.6† for results of ^1^H-NMR, ^13^C{^1^H}-NMR, HR-MS, ^1^H–^1^H COSY-NMR and DOSY-NMR analysis).

### DFT and transport calculations

Using the density functional code SIESTA the optimum geometries of isolated molecules were obtained by relaxing the molecules until all forces on the atoms were less than 0.01 eV Å^−1^.^71,72^ A double-zeta plus polarization orbital basis set, norm-conserving pseudopotentials, an energy cut-off of 250 Rydbergs defining the real space grid were used and the local density approximation (LDA) was chosen as the exchange correlation functional. We also computed results using GGA and found that the resulting transmission functions were comparable with those obtained using LDA.^73–75^ This was also applied to find the optimum geometries of multiple assembled components (Fig. S41†), and to derive isosurfaces for the FMOs of these molecules (Fig. S42†). To calculate the optimum binding distance between any two components, we used DFT and the counterpoise method, which removes basis set superposition errors (BSSE). The binding distance *d* is defined as the distance between compound 1 and compound 2. Here, compound 1 is defined as entity A and compound 2 as entity B. The ground state energy of the total system is calculated using SIESTA and is denoted *E*^AB^_AB_. The energy of each entity is then calculated in a fixed basis, which is achieved using ghost atoms in SIESTA. Hence, the energy of the individual 1 in the presence of the fixed basis is defined as *E*^AB^_A_ and for the gold as *E*^AB^_B_. The binding energy is then calculated using the following equation: binding energy = *E*^AB^_AB_ − *E*^AB^_A_ − *E*^AB^_B_ (see ESI Section 2.3† for full details and results). The theoretical tilt angle for these molecules was calculated through an analysis of the experimental derived values for film thickness (ESI Section 2.4†). Transmission coefficient curves *T*(*E*) *were* obtained using the Gollum transport code and were calculated for the eight different junction configurations, based on the tilt angles in Table S2† (different curves of the same colour correspond to different title angles and the yellow line is the average), as shown in the right panels of Fig. S49–S52.†^69^ Following this the Seebeck coefficient (*S*) of these molecules could also be calculated and the full details and results relating to these calculations are shown in Section 2.6 of the ESI.†

### Au preparation

Template stripped gold (Au^TS^) was prepared by a modified method of Whitesides and Pinkhassik.^76,77^ A Si wafer (5 mm x 5 mm) was cleaned using an ultrasonic bath with acetone, methanol and isopropanol (IPA), then cleaned with oxygen plasma for 5 minutes. The cleaned wafer was glued onto the top surface of a thermal evaporated gold chip (100 nm thickness) with Epotek 353rd epoxy adhesive. The adhesive was cured for 40 minutes at 150 °C, then cooled down to room temperature. The Si contact with gold without epoxy and adhesive was carefully removed using a sharp blade and leaving an atomically flat Au surface. The prepared gold was scanned by AFM for 3–5 random spots for quality tests. For all cases, only the substrates with roughness below 0.2 nm were used for SAMs growth.

### SAMs growth

#### Growth of anthracene-based SAMs

1 mM solution of molecule (1–4) was prepared in toluene (>99.5%, Sigma Aldrich), with 10 minutes nitrogen bubbling for oxygenation. The prepared Au^TS^ was immersed into the solution, and incubated for 24 hours under nitrogen atmosphere, over which time the molecules spontaneously formed an organic thin film on the Au surface due to the specialized anchor and the intermolecular interaction. The SAMs modified Au was rinsed with toluene and IPA several times to wash-off the physisorped molecules. The sample, after rinsing, was blown with nitrogen for drying and incubated in vacuum oven (10^−2^ mbar) overnight at 35 °C for solvent evaporation.

### Zn-TPP deposition

The SAMs modified Au substrates were immersed in 100 μM Zn-TPP solutions dissolved in toluene for Zn-TPP deposition. Among different deposition time, we found that 20 minutes of deposition gives a Zn-TPP layer with thickness relatively comparable with a single Zn-TPP lying on a flat surface. SAM growth was monitored by QCM where the same growth procedure was applied to a QCM substrate and the resonance frequency of the substrate before and after SAMs growth was compared to determine the amount of molecule absorbed through use of the Sauerbrey equation (see ESI Section 3.2†).^78^

### Electric and thermoelectric characterization

A modified AFM system was used for electric and thermoelectric characterization. The electrical transport properties of the SAMs were characterized by a cAFM system. The cAFM setup is based on a Multi-mode 8 AFM system (Bruker Nano Surfaces). The bottom gold substrate was used as the source, and a Pt/Cr coated probe (Multi75 E, BugetSensors) was used as the drain. The force between probe and molecule was controlled at 2 nN, as this force is strong enough for the probe to penetrate through the water layer on the sample surface but not too strong so as to destroy the molecular thin film. The triangular shape AC bias was added between the source and drain by a voltage generator (Aglient 33500B), the source to drain current was acquired by a current pre-amplifier (SR570, Stanford Research Systems) providing current-to-voltage conversion. The *I*–*V* characteristics were obtained by Nanoscope 8 controller simultaneously collecting drive bias and current with subsequent correlation of these values at each time point. The Seebeck coefficients of SAMs were obtained by a ThEFM, modified from the cAFM system used for electrical transport measurement. A Peltier stage driven by a voltage generator (Agilent 33500B, with auxiliary amplifier to provide sufficient drive current) and was used for heating up and cooling the sample, therefore providing a temperature difference between sample and probe. The probe used was a commercially available Pt coated probe coated with additional layers of 5 nm Cr and 30 nm Au to enlarge the contact area for voltage stability. The graphene was coated on the top of the probe with same method as described in the cAFM section. The sample temperature was measured by a Type T thermal couple, and the probe temperature can be assumed to be equal to ambient temperature as is in the case of low thermal conductivity sample's (organic film) as shown elsewhere.^33^ The thermal voltage between sample and probe was amplified by a high impedance differential pre-amplifier (SR560, Stanford Research Systems), and recorded by a computer.

## Author contributions

N. J. L., B. J. R., C. J. L. and A. I. conceived the research. T. L. R. B. synthesised the molecules and performed their characterisation and spectroscopic measurements. M. A., A. A. and A. I. carried out the theoretical simulations. S. A.-Y., X. W. and B. J. R. performed the self-assembly of the molecules and carried out their thermoelectronic characterisation. S. A.-Y. and S. P. J. performed XPS measurements. All co-authors assisted in writing the manuscript. L. A. W, T. A., S. P. J., L. F. C., A. I., C. J. L., B. J. R. and N. J. L. supervised the research and provided essential contributions to interpreting the results and drafting the manuscript.

## Conflicts of interest

There are no conflicts to declare.

## Supplementary Material

SC-013-D2SC00078D-s001
