# Supplementary material for: Multi-component self-assembled molecular-electronic films: towards new high-performance thermoelectric systems
Source: Chem Sci. 2022 Apr 15;13(18):5176–85. doi: 10.1039/d2sc00078d (PMC9093172; doi:10.1039/d2sc00078d)
Supplement: SC-013-D2SC00078D-s001 [file SC-013-D2SC00078D-s001.pdf]

Electronic Supplementary Material (ESI) for Chemical Science.
This journal is © The Royal Society of Chemistry 2022

Multi-Component Self-Assembled Molecular-Electronic Films: Towards New High-Performance Thermoelectric Systems

Troy L. R. Bennett,[a] Majed Alshammari,[b,c] Sophie Au-Yong,[b] Ahmad Almutlg,[b,d] Xintai Wang,[b,e] Luke A. Wilkinson,[f] Tim Albrecht,[g] Samuel P. Jarvis,[b] Lesley F. Cohen,[e] Ali Ismael,[b,h] Colin J. Lambert,[b] Benjamin J. Robinson[b] and Nicholas J. Long[a]

[a]Department of Chemistry, Imperial College London, MSRH, White City, London, W12 0BZ, UK. [b]Physics Department, Lancaster University, Lancaster, LA1 4YB, UK. [c]Department of Physics, College of Science, Jouf University, Skaka, Saudi Arabia. [d]Department of Mathematics, College of Science, Qassim University, Almethnab, Saudi Arabia. [e]The Blackett Laboratory, Imperial College London, South Kensington Campus, London, SW7 2AZ, UK. [f]Department of Chemistry, University of York, Heslington, York, YO10 5DD, UK. [g]Department of Chemistry, Birmingham University, Edgbaston, Birmingham, B15 2TT, UK. [h]Department of Physics, College of Education for Pure Science, Tikrit University, Tikrit, Iraq.

Contents

1. Compound Synthesis and Characterization 2
1.1 Materials and Methods 2
1.2 Instrumentation 2
1.3 Synthesis 2
1.4 Coordination of Anthracenes (1-3) to Zinc-Tetraphenylporphyrin (Zn-TPP) 5
1.5 NMR Comparisons 24
1.6 Diffusion-Ordered NMR Spectroscopy (DOSY) Experiments 27
2. DFT and Transport Calculations 31
2.1 Optimised DFT Structures of Isolated Molecular-scale Structures 31
2.2 Frontier orbitals 33
2.3 Binding energies 34
2.3.1 Binding energy of two components 34
2.3.2 Binding energy of molecules on Au 36
2.4 The tilt angle (θ) 38
2.5 Transport calculations 38
2.6 Seebeck coefficient 41
3. Formation and Thermoelectric Characterisation of SAMs 43
3.1 Au preparation 43
3.2 SAMs growth 43
3.3 SAM Topography 44
3.4 Electric and thermoelectric characterization 47
4. X-Ray Photoelectron Spectroscopy 53
4.1 XPS Characterisation of Au/1, Au/1/P, Au/2 and Au/2/P 53
5. References 56

1. Compound Synthesis and Characterization

1.1 Materials and Methods

The synthesis of molecules (**1-3**) has previously been reported.[1,2] All reactions were performed with the use of standard air-sensitive chemistry and Schlenk line techniques, under an atmosphere of nitrogen. No special precautions were taken to exclude air during any work-ups. 1,5-Dibromoanthracene was synthesised through the use of an adapted literature procedure.[3] All other reagents are commercially available and were used as received from suppliers, without further purification. Solvents used in reactions were collected from towers sparged with nitrogen and dried with 3 Å molecular sieves, apart from DIPA, which was distilled onto activated 3 Å molecular sieves under nitrogen.

1.2 Instrumentation

^{1}H- and ^{13}C{^{1}H}-NMR spectra were recorded on a Bruker Avance 400 MHz spectrometer and referenced to the residual solvent peaks of either $CDCl_3$ at 7.26 and 77.2 ppm or $CDCl_2$ at 5.32 or 54.0 ppm, respectively. ^{1}H-NMR spectra were fully assigned using 2D correlation spectroscopy. Coupling constants are measured in Hz. Mass spectrometry analyses were conducted by Dr. Lisa Haigh of the Mass Spectrometry Service, Imperial College London.

1.3 Synthesis

1,5-Di(4-(ethynyl)pyridine)anthracene (**4**)[4]

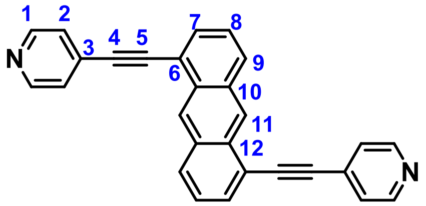

1,5-Dibromoanthracene (0.20 g, 0.60 mmol), 4-ethynylpyridine hydrochloride (0.33 g, 2.38 mmol) and CuI (0.01 g, 0.06 mmol) were dissolved in toluene (100 mL) and DIPA (20 mL). The solution was degassed for 15 minutes and Pd(P-^{t}Bu$_3$)$_2$ was added. The solution was then stirred at 50°C for >16 hours. The solvent was removed in vacuo to give a black solid that was purified by flash chromatography on an alumina V column, eluting with 1:1 hexane:DCM to give the final product as a yellow solid (0.17 g, 0.45 mmol, 75%).

^{1}H-NMR (CD_2Cl_2, 298 K, 400 MHz): δ_H = 9.02 (s, 2H, *H11*), 8.68 (dd, $^3J_{H-H}$ = 4.4, $^4J_{H-H}$ = 1.6 Hz, 4H *H1*), 8.21 (dd, $^3J_{H-H}$ = 8.4, $^4J_{H-H}$ = 0.8 Hz, 2H, *H9*), 7.90 (dd, $^3J_{H-H}$ = 8.0, $^4J_{H-H}$ = 1.2 Hz, 2H, *H7*), 7.61-7.55 (m, 6H, *H2*, *H8*) ppm; **^{13}C{^{1}H}-NMR** ($CDCl_3$, 298 K, 100 MHz): δ_C = 150.1 (Ar-C-H), 131.9 (Ar-C-H), 131.8 (Ar-C-C), 131.6 (Ar-C-C), 131.3 (Ar-C-C), 130.7 (Ar-C-H), 125.9 (Ar-C-H), 125.8 (Ar-C-H), 125.5 (Ar-C-H), 120.0 (Ar-C-H), 92.2 (-C≡C-), 92.2 (-C≡C-) ppm; **MS** ES+: calcd. for $C_{28}H_{17}N_2$ [M+H$^+$] calcd. 381.1392; found. 381.1378.

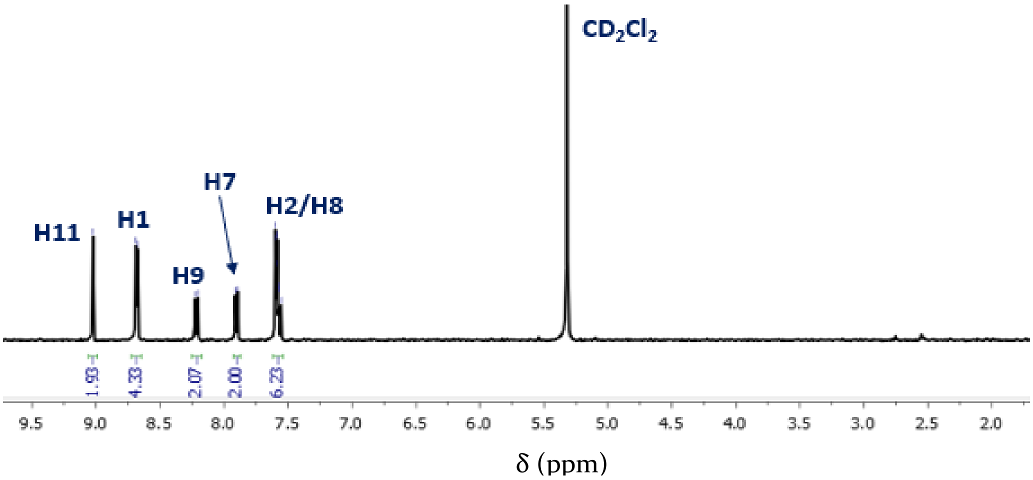

Figure S1: The ^{1}H NMR spectrum of **4** in CD_2Cl_2.

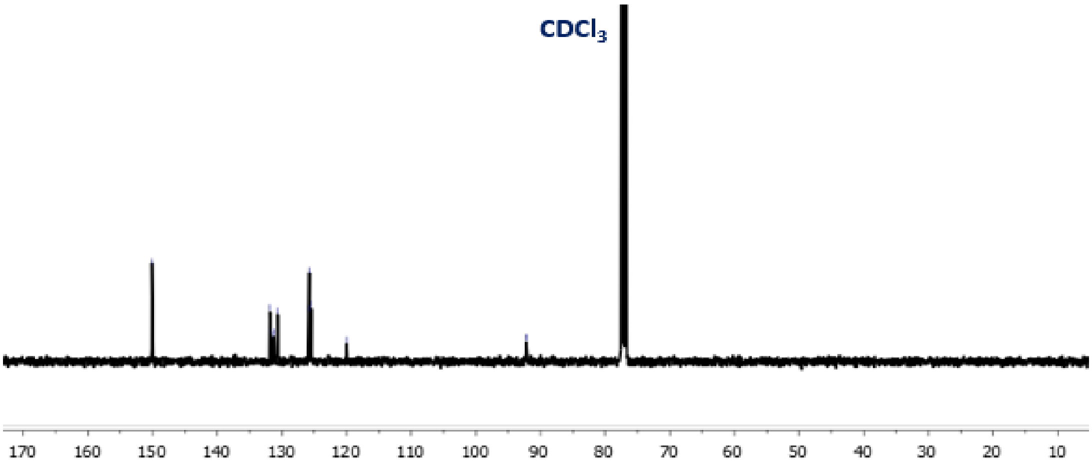

Figure S2: The $^{13}C\{^{1}H\}$ NMR spectrum of **4** in $CDCl_3$.

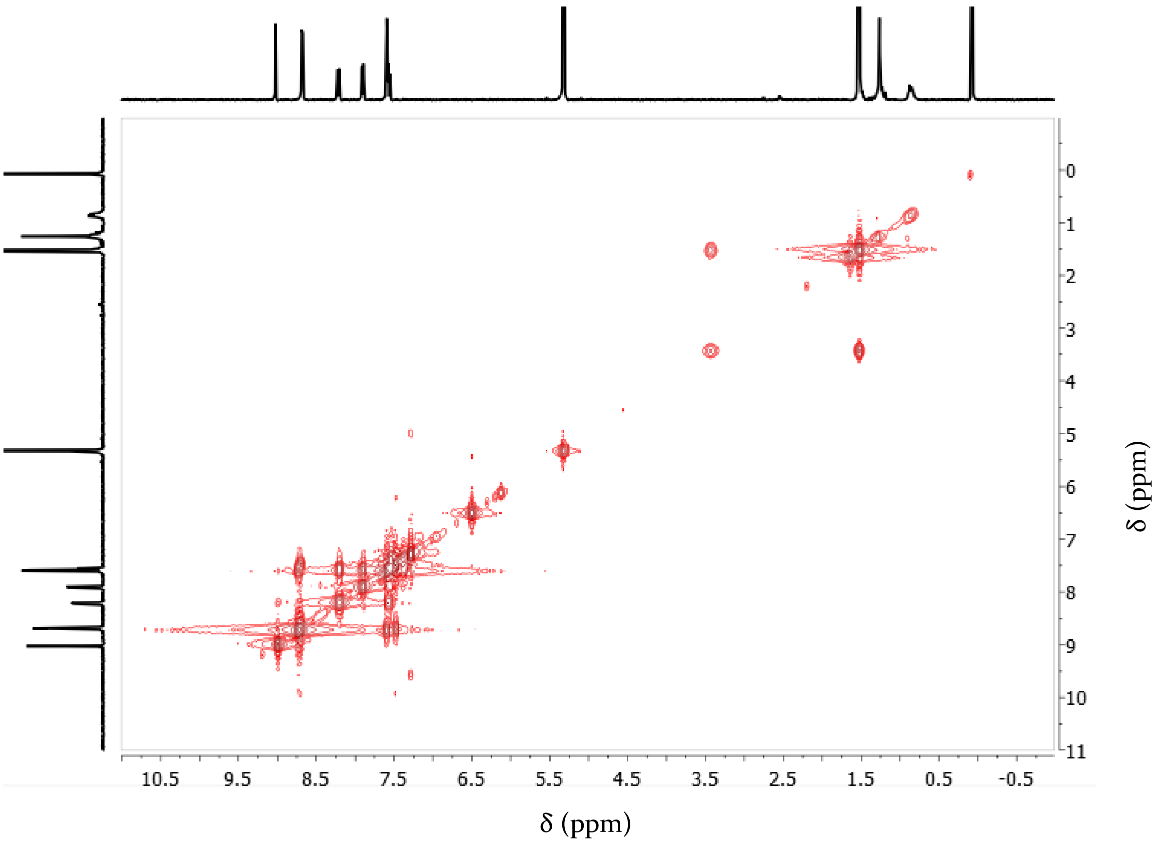

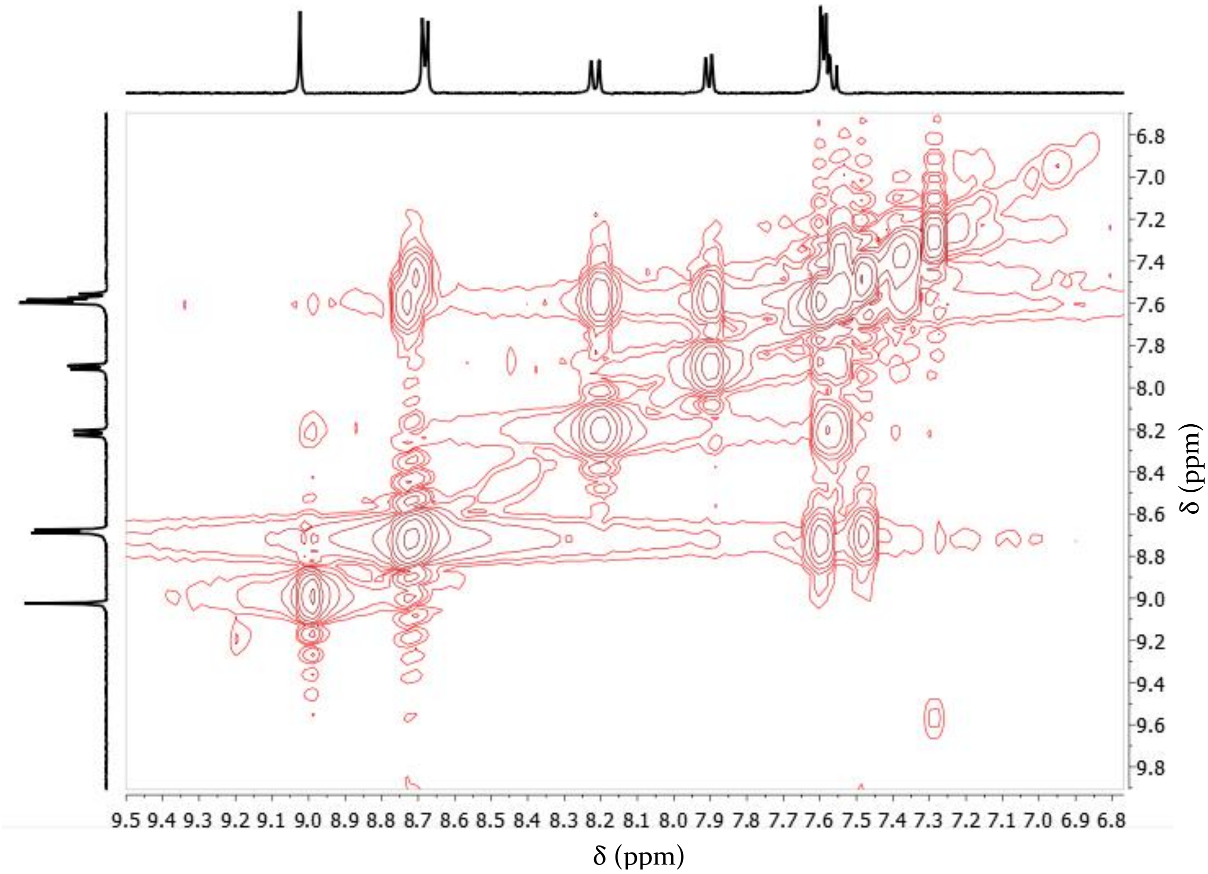

Figure S3: The ¹H-¹H COSY-NMR spectrum of **4** in $CDCl_3$. Full spectrum (top) and a zoomed region (bottom).

1.4 Coordination of Anthracenes (1-3) to Zinc-Tetraphenylporphyrin (Zn-TPP)

General Procedure: To evaluate the coordinative behaviour of the synthesised anthracene derivatives, we evaluated NMR data produced from both 1:1 and 2:1 ratios of anthracene:porphyrin. For each experiment 10 mg of anthracene was dissolved in 20 mL of DCM. Either 1 or 0.5 equivalents of **Zn-TPP** were added, to form 6 different samples, the solutions were then stirred for 1 hour before the solvent was removed *in vacuo* to give a purple solid that was analysed. Characterisation data of molecules **1-3** is included for the sake of comparison. (Proposed molecular structures included within this section are provided as a guide for the reader rather than a definitive molecular identity).

Molecule 1[1]

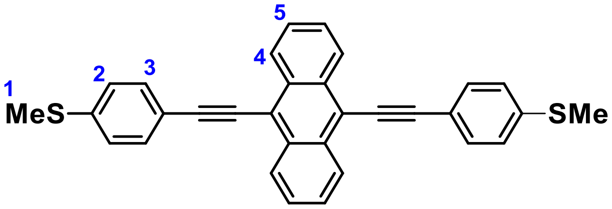

^{1}H-NMR ($CDCl_3$, 298 K, 400 MHz): δ_H = 8.69 (dd, $^3J_{H-H}$ = 6.8, $^4J_{H-H}$ = 3.2 Hz, 4H, *H4*), 7.70 (d, $^3J_{H-H}$ = 8.4 Hz, 4H, *H3*), 7.67 (dd, $^3J_{H-H}$ = 6.8, $^4J_{H-H}$ = 3.2 Hz, 4H, *H5*), 7.32 (d, $^3J_{H-H}$ = 8.4 Hz, 4H, *H2*), 2.55 (s, 6H, *H1*) ppm; **^{13}C{^{1}H}-NMR** ($CDCl_3$, 298 K, 100 MHz): δ_C = 140.2 (Ar-C-C), 132.2 (Ar-C-C), 132.1 (Ar-C-H), 127.4 (Ar-C-H), 126.9 (Ar-C-H), 126.2 (Ar-C-H), 119.8 (Ar-C-C), 118.6 (Ar-C-C), 102.5 (-C≡C-), 86.9 (-C≡C-), 15.6 (S-CH_3) ppm; **MS** ES+: calcd. for $C_{32}H_{22}S_2$ [M]+ 469.1079; found. 469.1077.

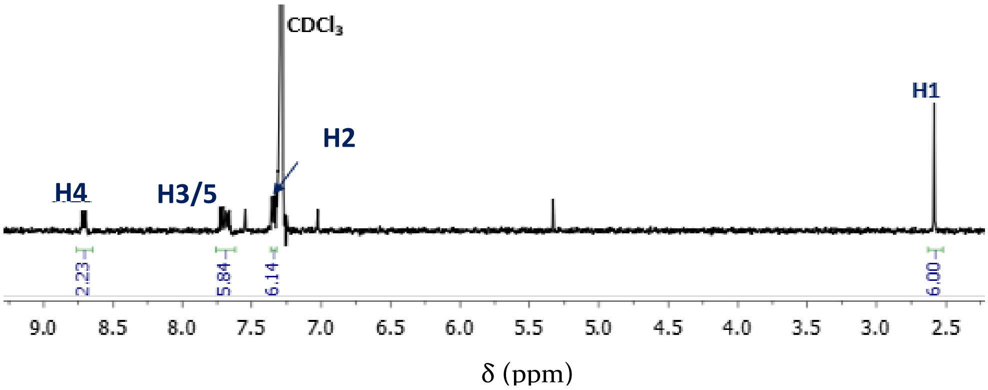

Figure S4: The ^{1}H NMR spectrum of **1** in $CDCl_3$.

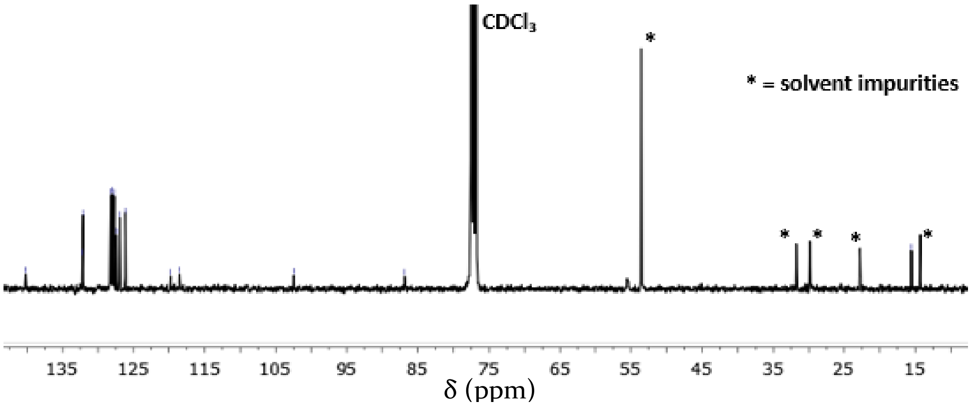

Figure S5: The ^{13}C{^{1}H} NMR spectrum of **1** in $CDCl_3$.

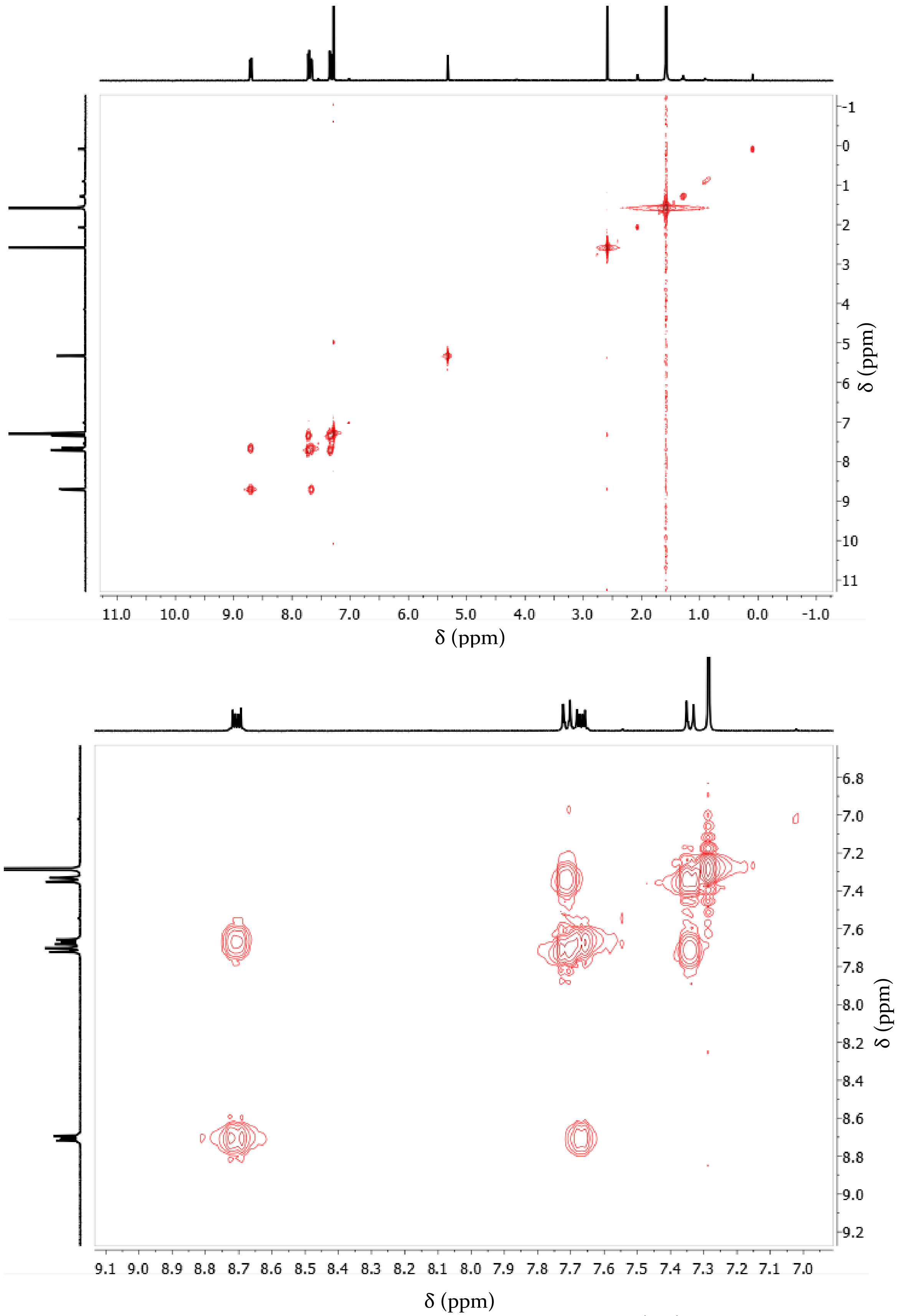

Figure S6: The ^{1}H-^{1}H COSY-NMR spectrum of **1** in $CDCl_3$. Full spectrum (top) and a zoomed region (bottom).

Molecule 1:Zn-TPP (1:1 ratio)

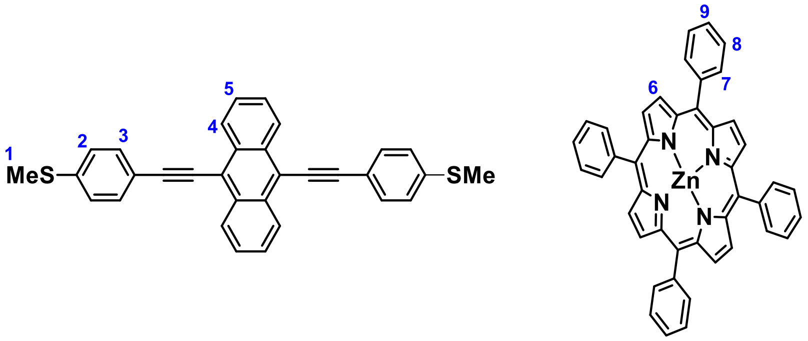

^{1}H-NMR ($CDCl_3$, 298 K, 400 MHz): δ = 8.95 (s, 8H, *H6*), 8.70-8.64 (m, 4H, *H4*), 8.24-8.19 (m, 8H, *H7*), 7.80-7.71 (m, 12H, *H8*, *H9*), 7.68 (dd, $^{3}J_{H\text{-}H}$ = 6.4, $^{4}J_{H\text{-}H}$ = 2.0 Hz, 4H, *H3*), 7.66-7.62 (m, 4H, *H5*), 7.30 (dd, $^{3}J_{H\text{-}H}$ = 6.4, $^{4}J_{H\text{-}H}$ = 2.0 Hz, 4H, *H2*), 2.54 (s, 6H, *H1*) ppm; **^{13}C{^{1}H}-NMR** ($CDCl_3$, 298 K, 100 MHz): δ = 150.4 (Ar-C-H), 143.0 (Ar-C-C), 140.1 (Ar-C-C), 134.6 (Ar-C-H), 132.2 (Ar-C-C), 132.1 (Ar-C-H), 132.1 (Ar-C-H), 127.6 (Ar-C-C), 127.4 (Ar-C-H), 126.9 (Ar-C-H), 126.7 (Ar-C-H), 126.2 (Ar-C-H), 121.3 (Ar-C-C), 119.8 (Ar-C-C), 118.6 (Ar-C-C), 102.5 (-C≡C-), 86.9 (-C≡C-), 15.6 (S-CH_3) ppm.

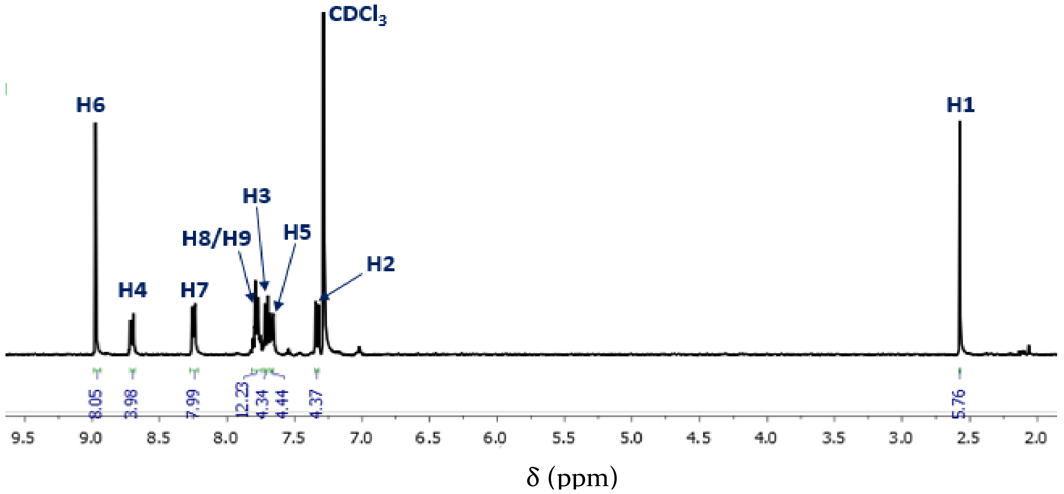

Figure S7: The ^{1}H NMR spectrum of a 1:1 ratio of **1:Zn-TPP**, in $CDCl_3$.

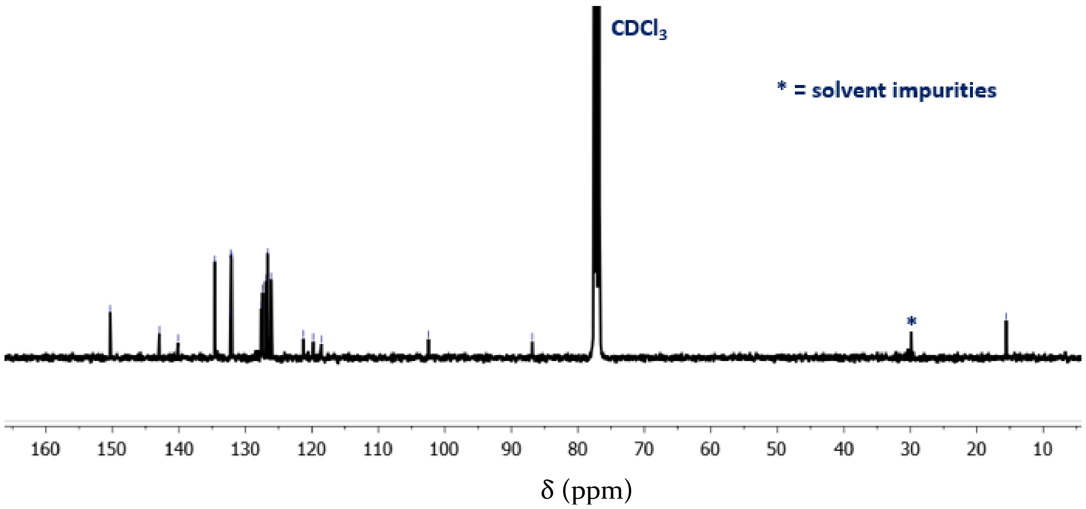

Figure S8: The ^{13}C{^{1}H} NMR spectrum of a 1:1 ratio of **1:Zn-TPP**, in $CDCl_3$.

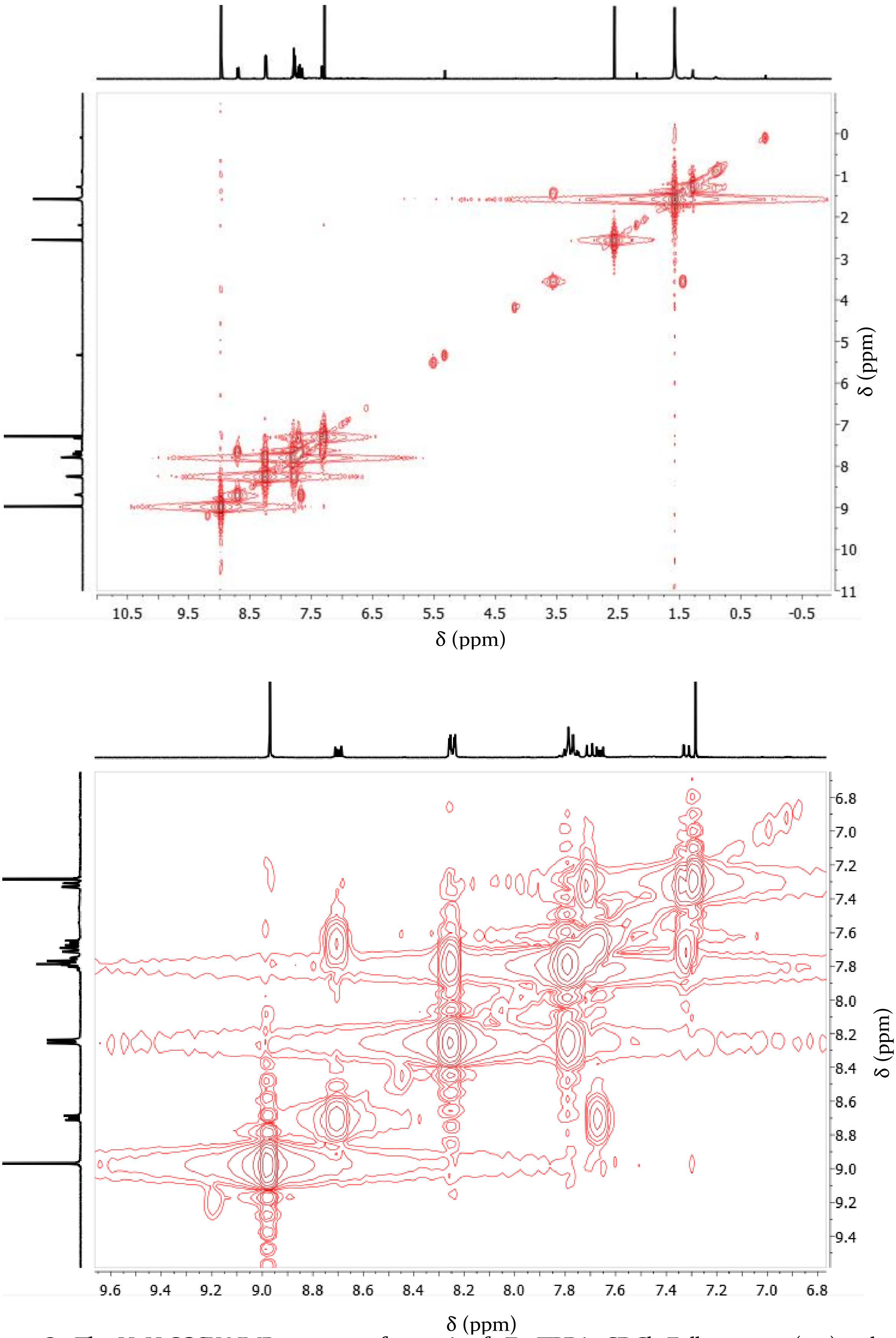

Figure S9: The ^{1}H-^{1}H COSY-NMR spectrum of a 1:1 ratio of **1:Zn-TPP** in $CDCl_3$. Full spectrum (top) and a zoomed region (bottom).

Molecule 1:Zn-TPP (2:1 ratio)

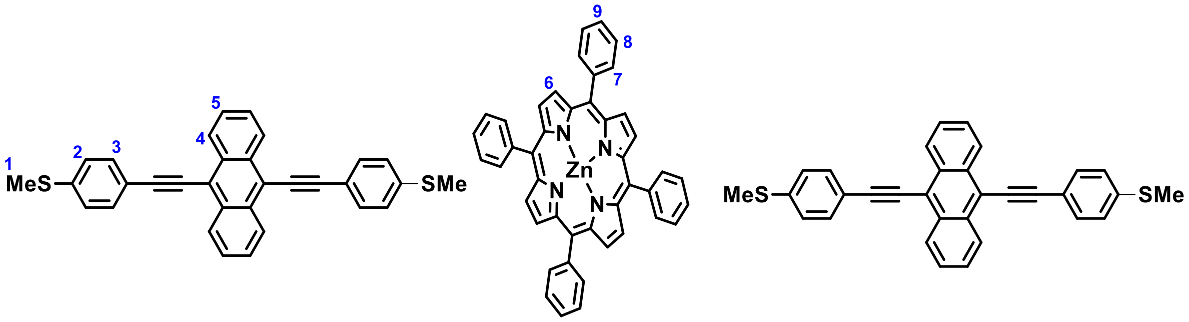

^{1}H-NMR ($CDCl_3$, 298 K, 400 MHz): δ = 8.95 (s, 8H, *H6*), 8.70-8.64 (m, 8H, *H4*), 8.24-8.19 (m, 8H, *H7*), 7.80-7.71 (m, 12H, *H8, H9*), 7.68 (dd, $^3J_{H-H}$ = 6.4, $^4J_{H-H}$ = 2.0 Hz, 8H, *H3*), 7.66-7.62 (m, 8H, *H5*), 7.30 (dd, $^3J_{H-H}$ = 6.4, $^4J_{H-H}$ = 2.0 Hz, 8H, *H2*), 2.54 (s, 12H, *H1*) ppm; **$^{13}C\{^1H\}$-NMR** ($CDCl_3$, 298 K, 100 MHz): δ = 150.4 (Ar-C-H), 143.0 (Ar-C-C), 140.1 (Ar-C-C), 134.6 (Ar-C-H), 132.2 (Ar-C-C), 132.1 (Ar-C-H), 132.1 (Ar-C-H), 127.6 (Ar-C-C), 127.4 (Ar-C-H), 126.9 (Ar-C-H), 126.7 (Ar-C-H), 126.2 (Ar-C-H), 121.3 (Ar-C-C), 119.8 (Ar-C-C), 118.6 (Ar-C-C), 102.5 (-C≡C-), 86.9 (-C≡C-), 15.6 (S-CH_3) ppm.

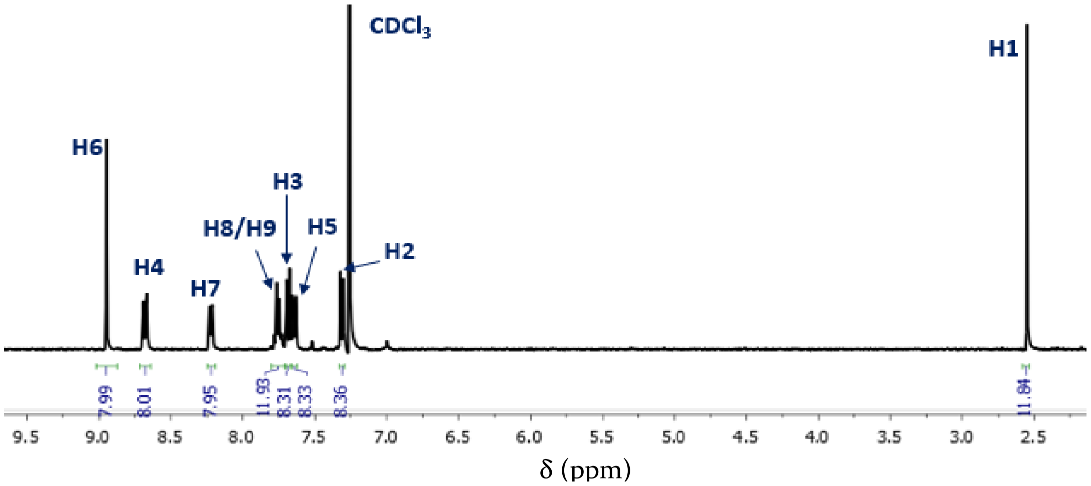

Figure S10: The ^{1}H NMR spectrum of a 2:1 ratio of **1:Zn-TPP**, in $CDCl_3$.

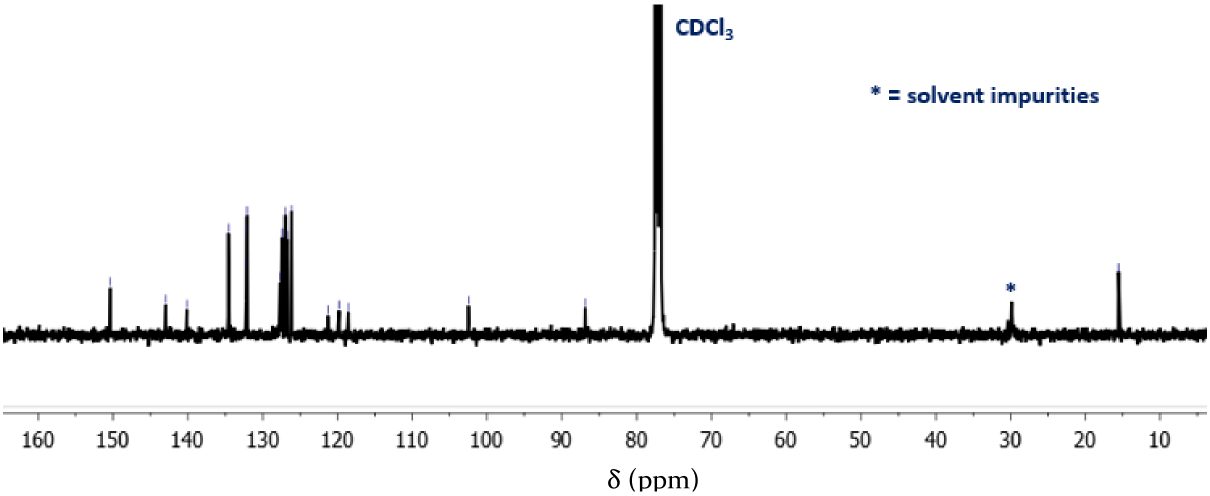

Figure S11: The $^{13}C\{^1H\}$ NMR spectrum of a 2:1 ratio of **1:Zn-TPP**, in $CDCl_3$.

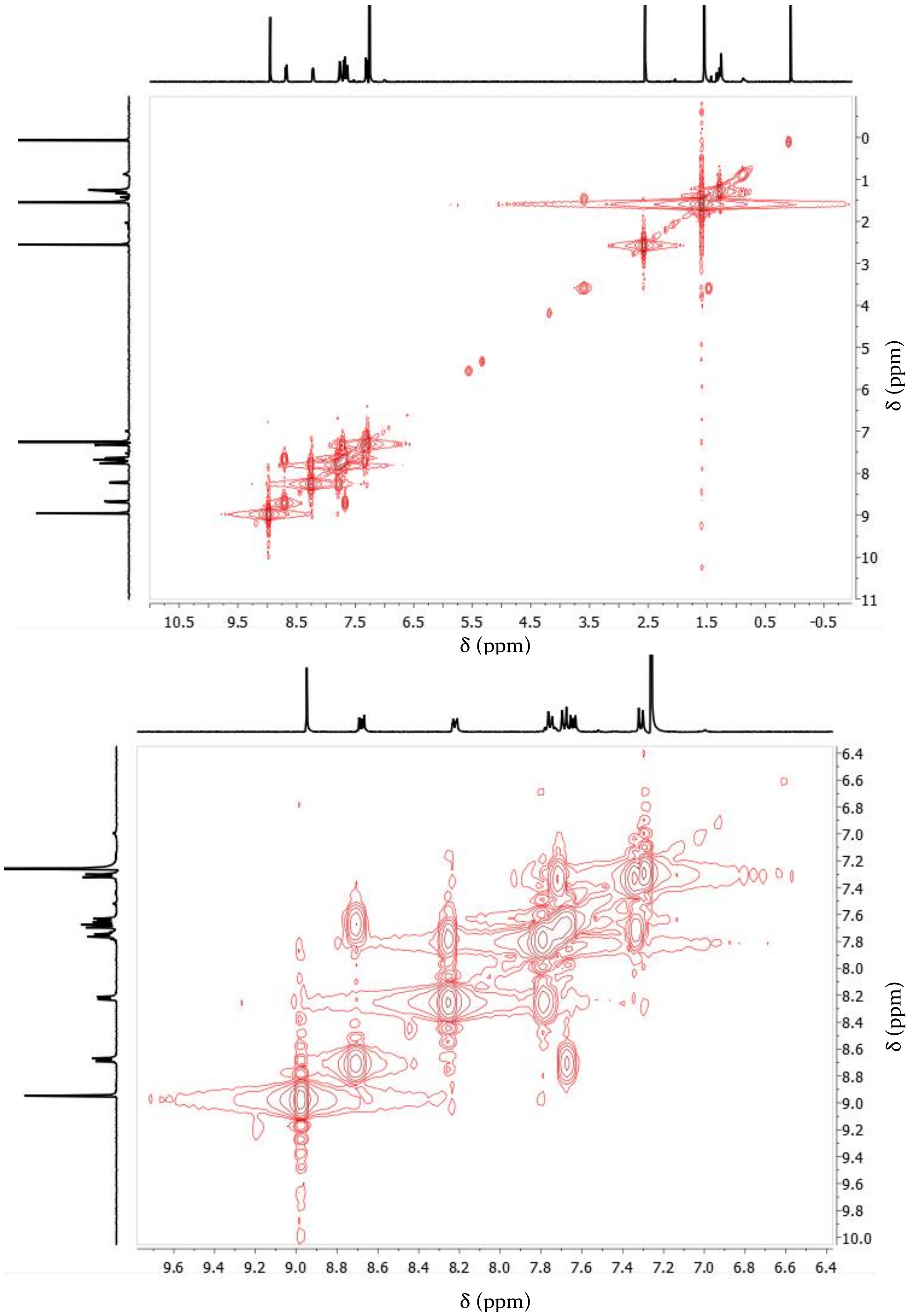

Figure S12: The ^{1}H-^{1}H COSY-NMR spectrum of a 2:1 ratio of **1:Zn-TPP** in $CDCl_3$. Full spectrum (top) and a zoomed region (bottom).

Molecule 2[1]

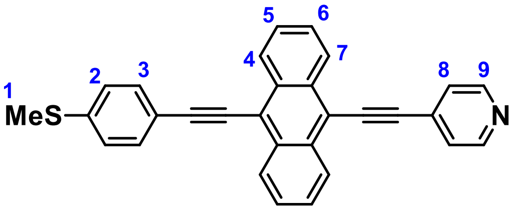

^{1}H-NMR ($CDCl_3$, 298 K, 400 MHz): δ = 8.75-8.65 (m, 6H, *H4, H7, H9*), 7.75-7.67 (m, 6H, *H5, H6, H8*), 7.64 (dd, $^3J_{H-H}$ = 4.4, $^4J_{H-H}$ = 1.6 Hz, 2H, *H3*), 7.34 (dd, $^3J_{H-H}$ = 4.4, $^4J_{H-H}$ = 1.6 Hz, 2H, *H2*), 2.56 (s, 3H, *H1*) ppm; **^{13}C{^{1}H}-NMR** ($CDCl_3$, 298 K, 100 MHz): δ = 150.1 (Ar-C-H), 140.5 (Ar-C-C), 132.5 (Ar-C-C), 132.1 (Ar-C-H), 132.0 (Ar-C-C), 131.6 (Ar-C-C), 127.6 (Ar-C-H), 127.5 (Ar-C-H), 127.0 (Ar-C-H), 127.0 (Ar-C-H), 126.1 (Ar-C-H), 125.6 (Ar-C-H), 120.2 (Ar-C-C), 119.5 (Ar-C-C), 116.7 (Ar-C-C), 103.2 (-C≡C-), 99.3 (-C≡C-), 91.2 (-C≡C-), 86.6 (-C≡C-), 15.5 (S-CH_3) ppm; **MS** ES+: calcd. for $C_{30}H_{19}NS$[M]+ 426.1311; found. 426.1310.

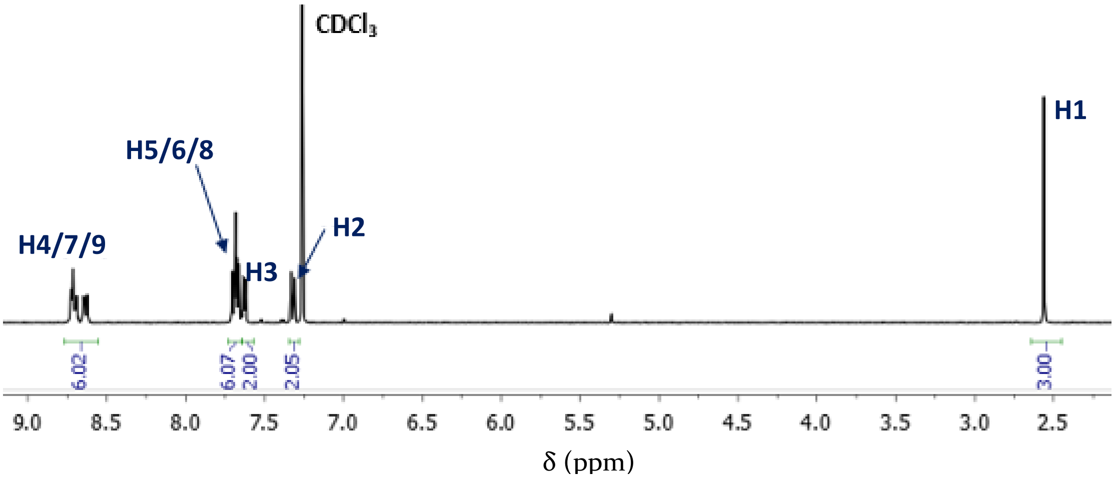

Figure S13: The ^{1}H NMR spectrum of **2** in $CDCl_3$.

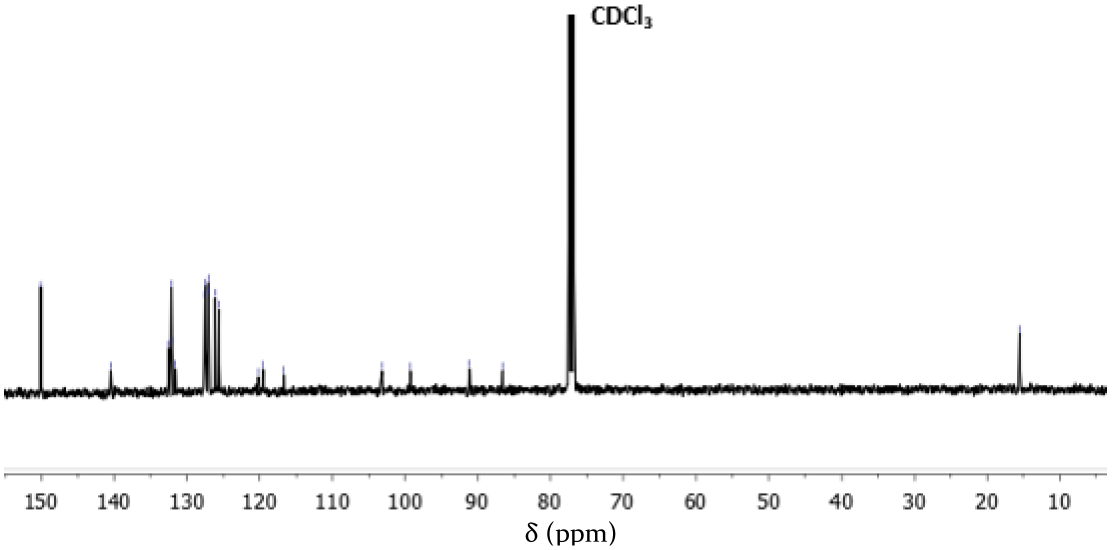

Figure S14: The ^{13}C{^{1}H} NMR spectrum of **2** in $CDCl_3$.

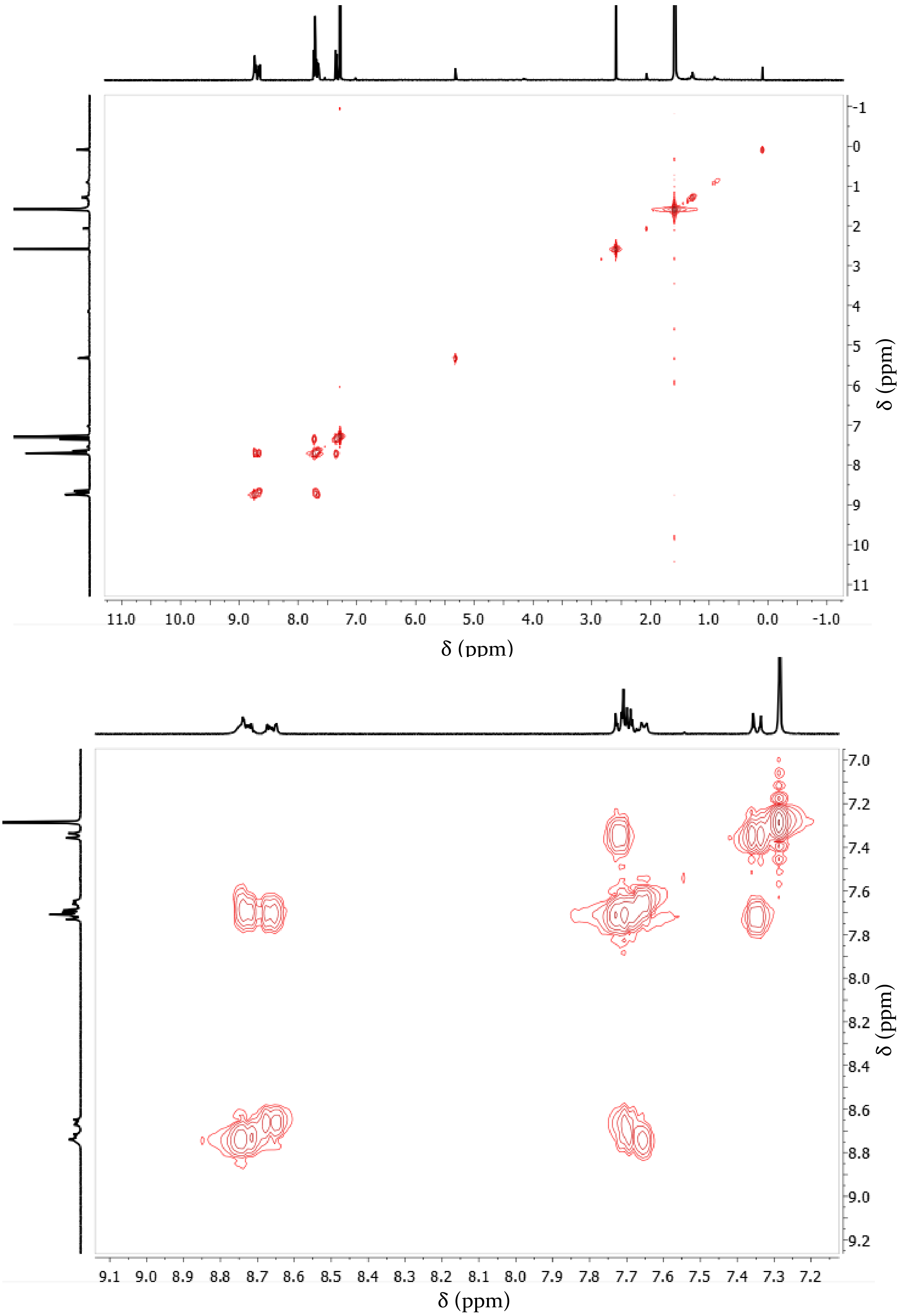

Figure S15: The ^{1}H-^{1}H COSY-NMR spectrum of **2** in $CDCl_3$. Full spectrum (top) and a zoomed region (bottom).

Molecule 2:Zn-TPP (1:1 ratio)

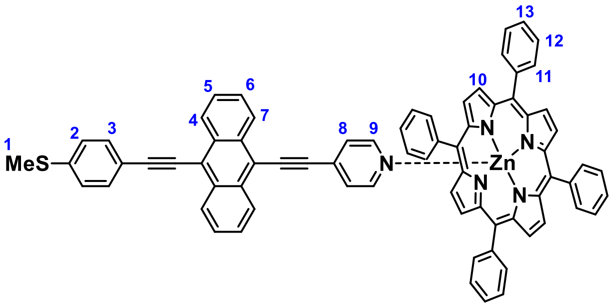

^{1}H-NMR ($CDCl_3$, 298 K, 400 MHz): δ = 8.90 (s, 8H, *H10*), 8.58 (d, $^3J_{H-H}$ = 8.8 Hz, 2H, *H4*), 8.25-8.20 (m, 10H, *H7*, *H11*), 7.79-7.70 (m, 12H, *H12*, *H13*), 7.62 (d, $^3J_{H-H}$ = 8.4 Hz, 2H, *H3*), 7.54 (pseudo t, $^3J_{H-H}$ = 7.2 Hz, 2H, *H5*), 7.48 (pseudo t, $^3J_{H-H}$ = 7.2 Hz, 2H, *H6*), 7.27 (d, $^3J_{H-H}$ = 8.4 Hz, 2H, *H2*), 6.50 (br s, 2H, *H9*), 4.81 (br s, 2H, *H8*), 2.47 (s, 3H, *H1*) ppm; **^{13}C{^{1}H}-NMR** ($CDCl_3$, 298 K, 100 MHz): δ = 150.2 (Ar-C-H), 143.9 (Ar-C-H), 143.6 (Ar-C-C), 140.5 (Ar-C-C), 134.8 (Ar-C-H), 132.2 (Ar-C-C), 132.0 (Ar-C-H), 131.9 (Ar-C-H), 131.7 (Ar-C-H), 131.4 (Ar-C-C), 128.2 (Ar-C-C), 127.3 (Ar-C-C), 127.3 (Ar-C-H), 126.8 (Ar-C-H), 126.5 (Ar-C-H), 126.4 (Ar-C-H), 126.0 (Ar-C-H), 124.2 (Ar-C-H), 120.8 (Ar-C-C), 120.4 (Ar-C-C), 119.3 (Ar-C-C), 115.7 (Ar-C-C), 103.3 (-C≡C-), 97.7 (-C≡C-), 92.2 (-C≡C-), 86.4 (-C≡C-), 15.4 ($S-CH_3$) ppm.

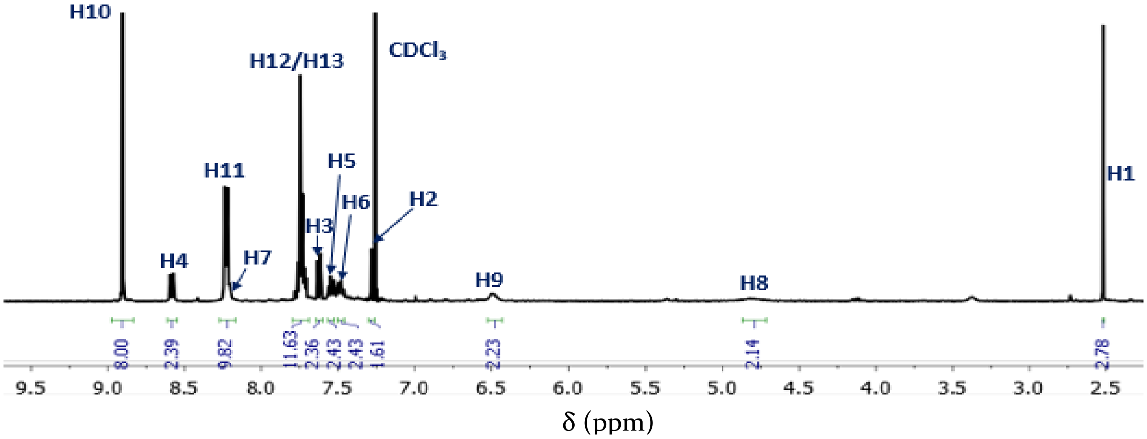

Figure S16: The ^{1}H NMR spectrum of a 1:1 ratio of **2**:**Zn-TPP**, in $CDCl_3$.

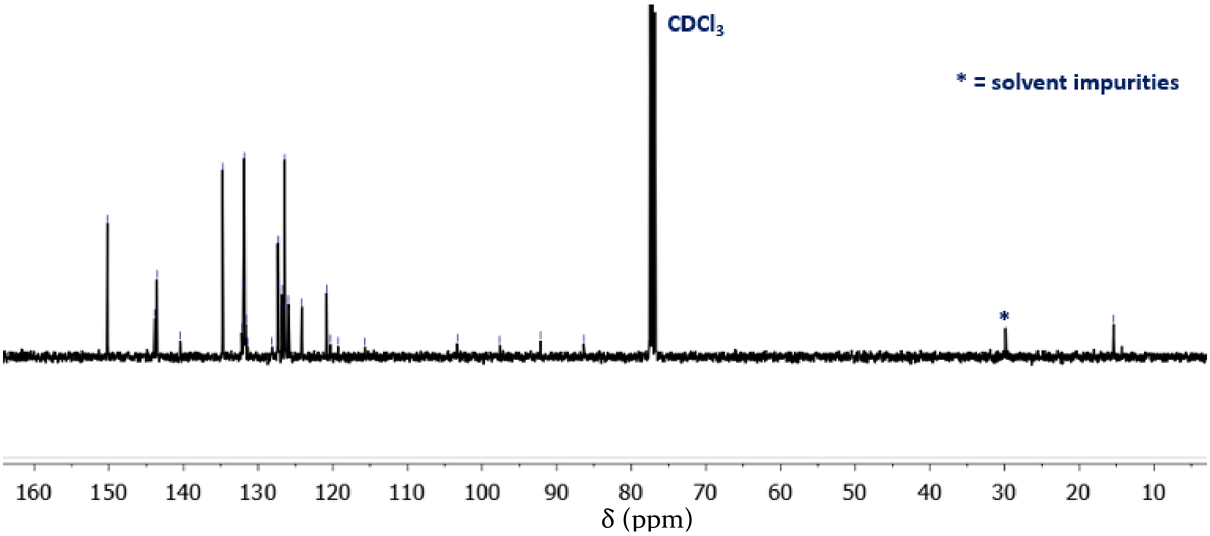

Figure S17: The ^{13}C{^{1}H} NMR spectrum of a 1:1 ratio of **2**:**Zn-TPP**, in $CDCl_3$.

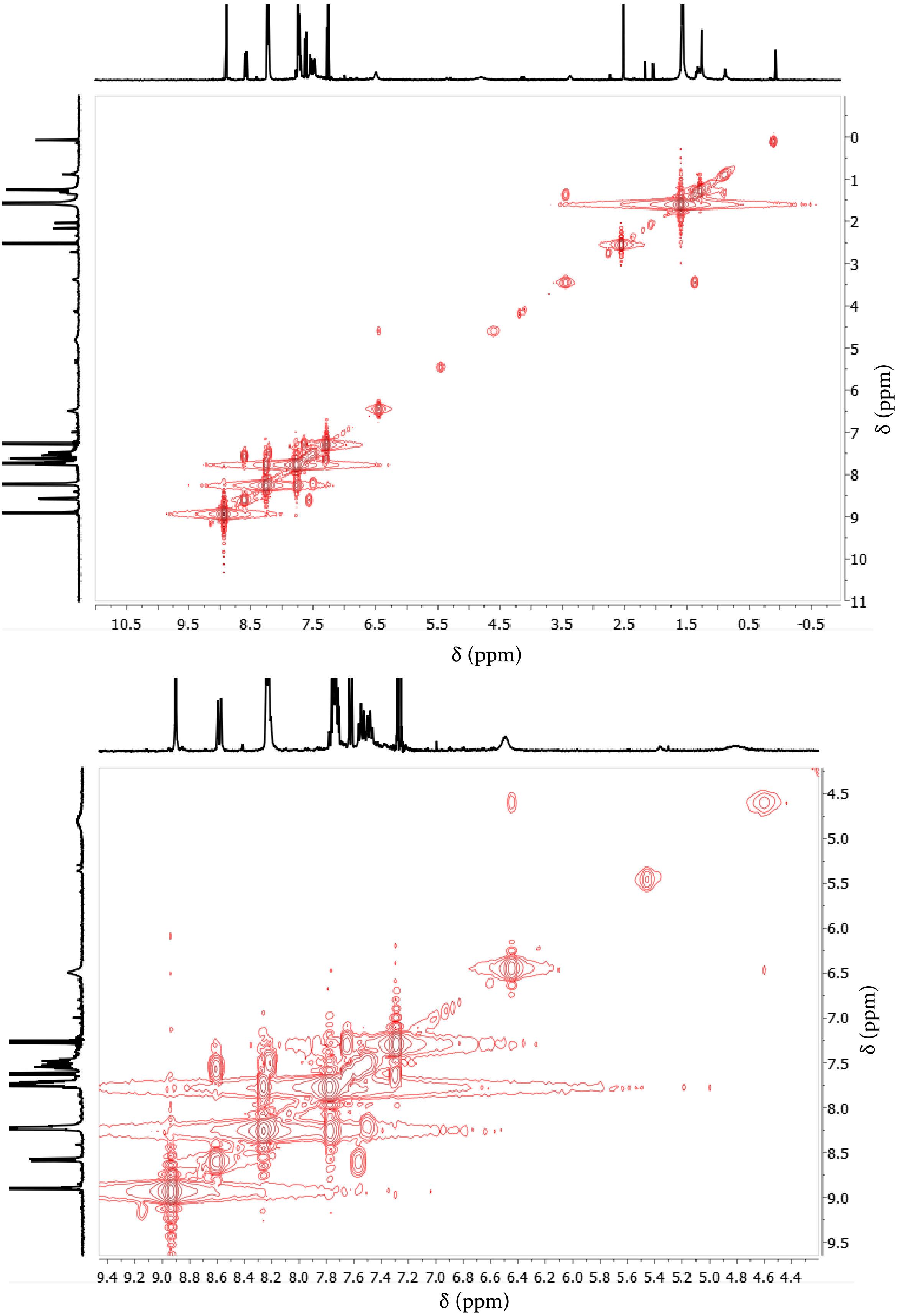

Figure S18: The ^{1}H-^{1}H COSY-NMR spectrum of a 1:1 ratio of **2:Zn-TPP** in $CDCl_3$. Full spectrum (top) and a zoomed region (bottom).

Molecule 2:Zn-TPP (2:1 ratio)

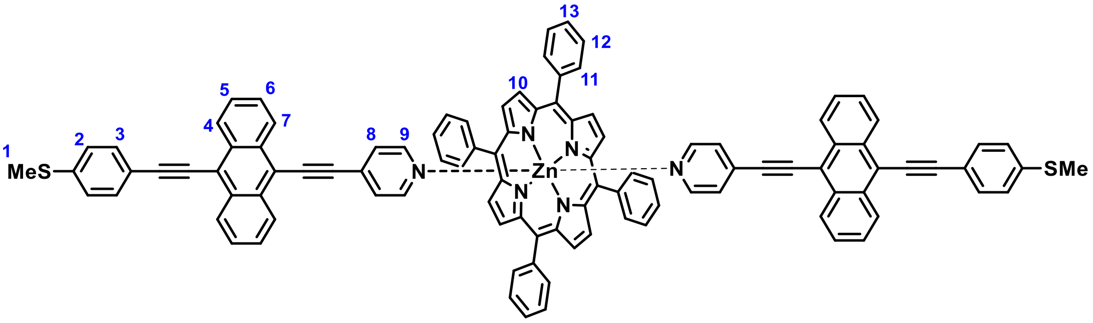

^{1}H-NMR ($CDCl_3$, 298 K, 400 MHz): δ = 8.90 (s, 8H, *H10*), 8.60 (d, $^3J_{H\text{-}H}$ = 8.8 Hz, 4H, *H4*), 8.30 (d, $^3J_{H\text{-}H}$ = 8.4 Hz, 4H, *H7*), 8.23 (dd, $^3J_{H\text{-}H}$ = 7.2, $^4J_{H\text{-}H}$ = 2.0 Hz, 8H, *H11*), 7.80-7.70 (m, 12H, *H12*, *H13*), 7.63 (d, $^3J_{H\text{-}H}$ = 8.4 Hz, 4H, *H3*), 7.57 (pseudo t, $^3J_{H\text{-}H}$ = 7.2 Hz, 4H, *H5*), 7.51 (pseudo t, $^3J_{H\text{-}H}$ = 7.2 Hz, 4H, *H6*), 7.26 (d, $^3J_{H\text{-}H}$ = 8.4 Hz, 4H, *H2*), 6.73 (br s, 4H, *H9*), 5.67 (br s, 4H, *H8*), 2.51 (s, 6H, *H1*) ppm; **^{13}C{^{1}H}-NMR** ($CDCl_3$, 298 K, 100 MHz): δ = 150.2 (Ar-C-H), 146.1 (Ar-C-H), 143.6 (Ar-C-C), 140.5 (Ar-C-C), 134.8 (Ar-C-H), 132.3 (Ar-C-C), 132.0 (Ar-C-H), 131.8 (Ar-C-H), 131.5 (Ar-C-C), 128.2 (Ar-C-C), 127.4 (Ar-C-C), 127.4 (Ar-C-H), 127.3 (Ar-C-H), 126.9 (Ar-C-H), 126.6 (Ar-C-H), 126.4 (Ar-C-H), 126.0 (Ar-C-H), 124.7 (Ar-C-H), 120.8 (Ar-C-C), 120.3 (Ar-C-C), 119.4 (Ar-C-C), 116.1 (Ar-C-C), 103.3 (-C≡C-), 98.2 (-C≡C-), 91.9 (-C≡C-), 86.5 (-C≡C-), 15.4 (S-CH_3) ppm.

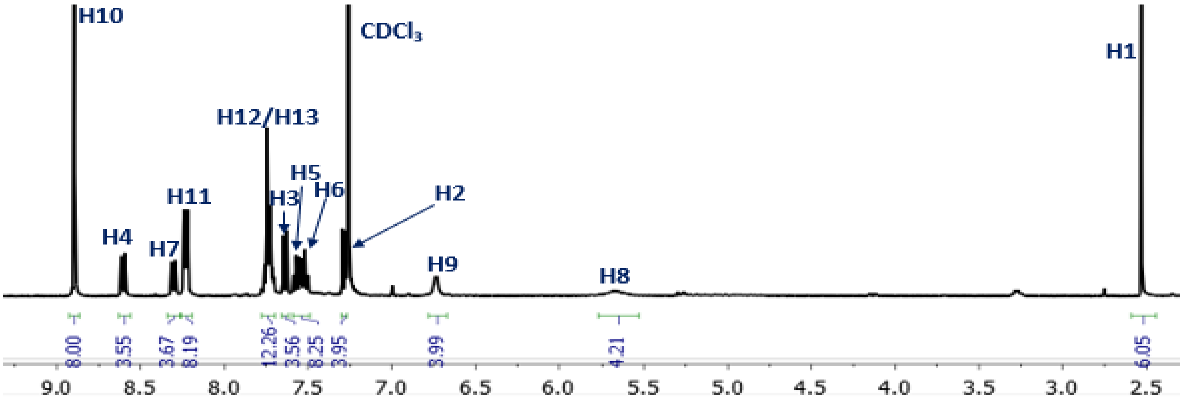

Figure S19: The ^{1}H NMR spectrum of a 2:1 ratio of **2**:**Zn-TPP**, in $CDCl_3$.

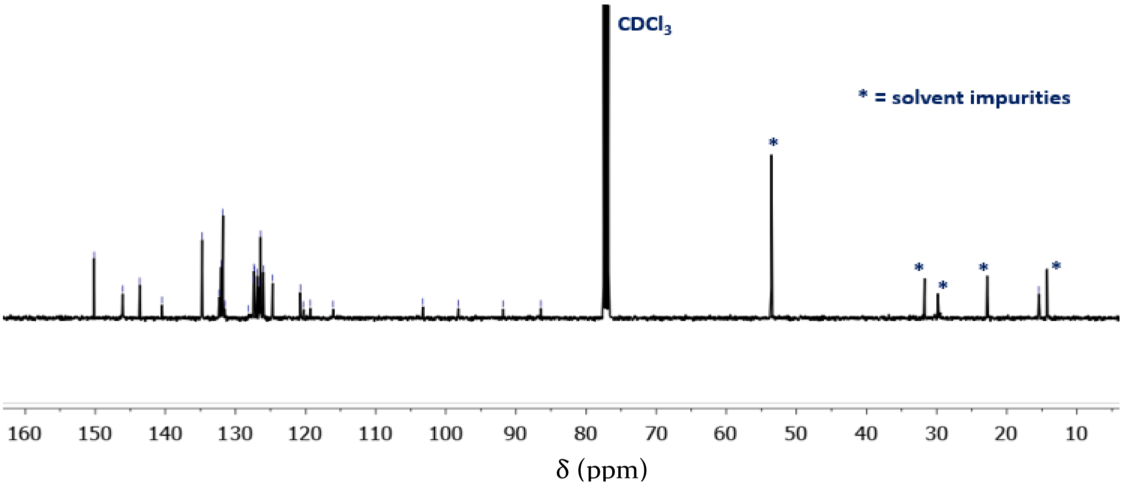

Figure S20: The ^{13}C{^{1}H} NMR spectrum of a 2:1 ratio of **2**:**Zn-TPP**, in $CDCl_3$.

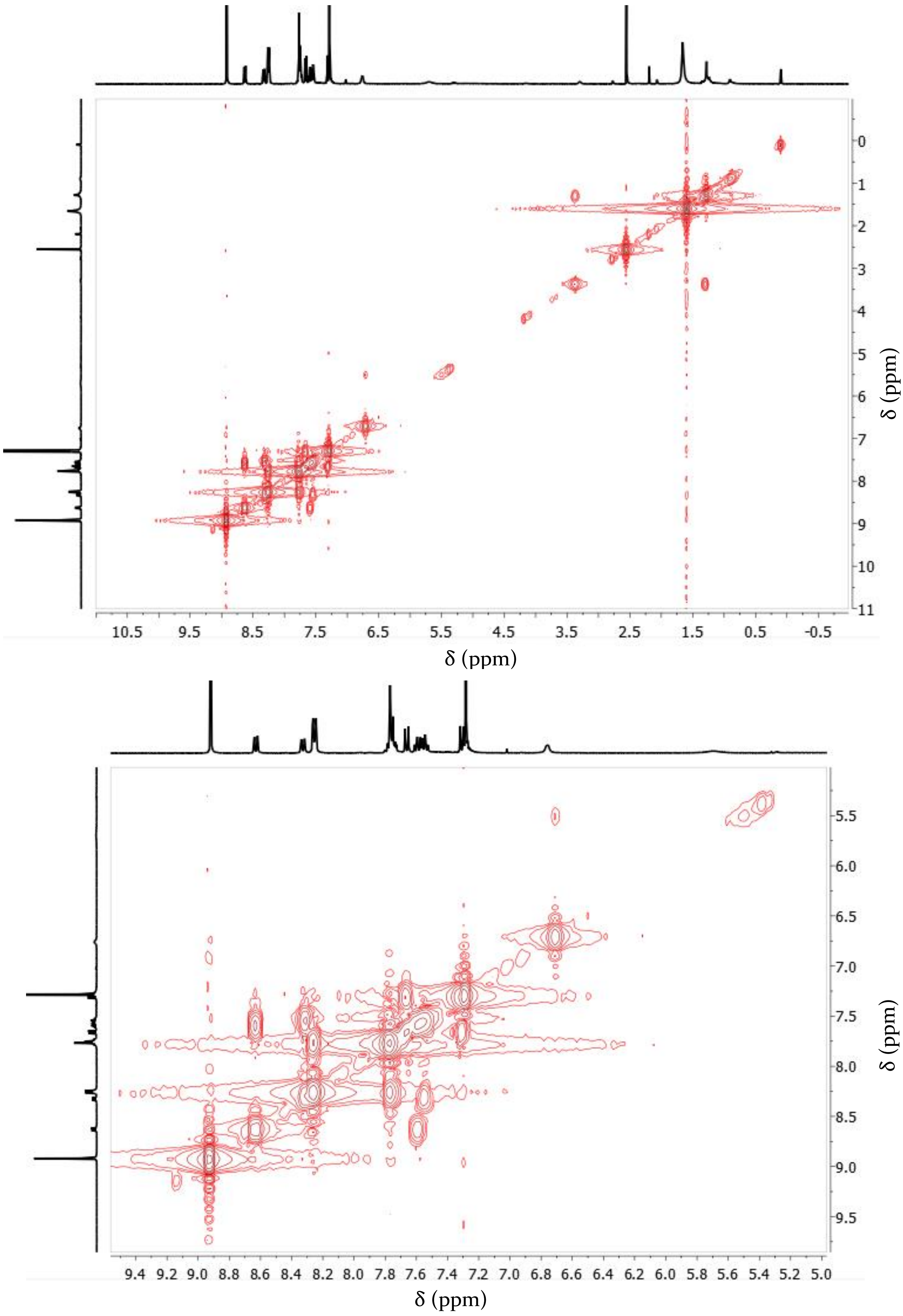

Figure S21: The ¹H-¹H COSY-NMR spectrum of a 2:1 ratio of **2:Zn-TPP** in $CDCl_3$. Full spectrum (top) and a zoomed region (bottom).

Molecule 3[1]

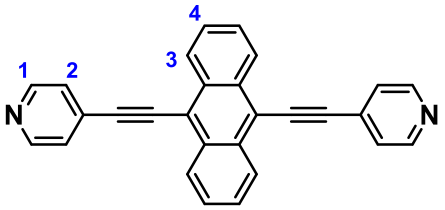

^{1}H-NMR ($CDCl_3$, 298 K, 400 MHz): δ = 8.73 (d, $^3J_{H-H}$ = 5.6 Hz, 4H, *H1*), 8.69-8.63 (m, 4H, *H3*), 7.74-7.68 (m, 4H, *H4*), 7.63 (d, $^3J_{H-H}$ = 5.6 Hz, 4H, *H2*) ppm; **^{13}C{^{1}H}-NMR** ($CDCl_3$, 298 K, 100 MHz): δ = 150.2 (Ar-C-H), 132.4 (Ar-C-C), 131.4 (Ar-C-C), 127.6 (Ar-C-H), 127.2 (Ar-C-H), 125.7 (Ar-C-H), 118.3 (Ar-C-C), 99.8 (-C≡C-), 90.9 (-C≡C-) ppm; **MS** ES+: calcd. for $C_{28}H_{16}N_2$ [M]+ 381.1388; found. 381.1392.

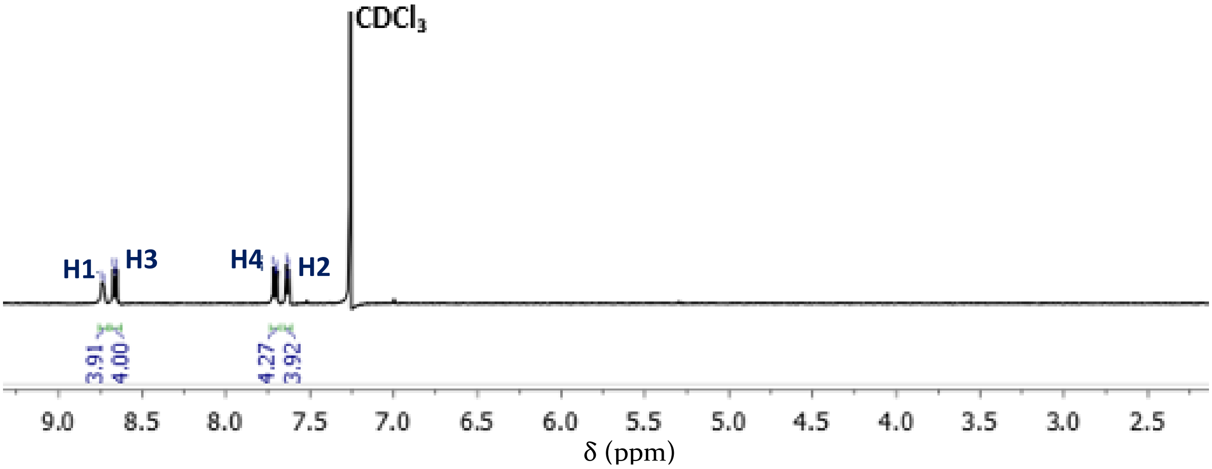

Figure S22: The ^{1}H NMR spectrum of **3** in $CDCl_3$.

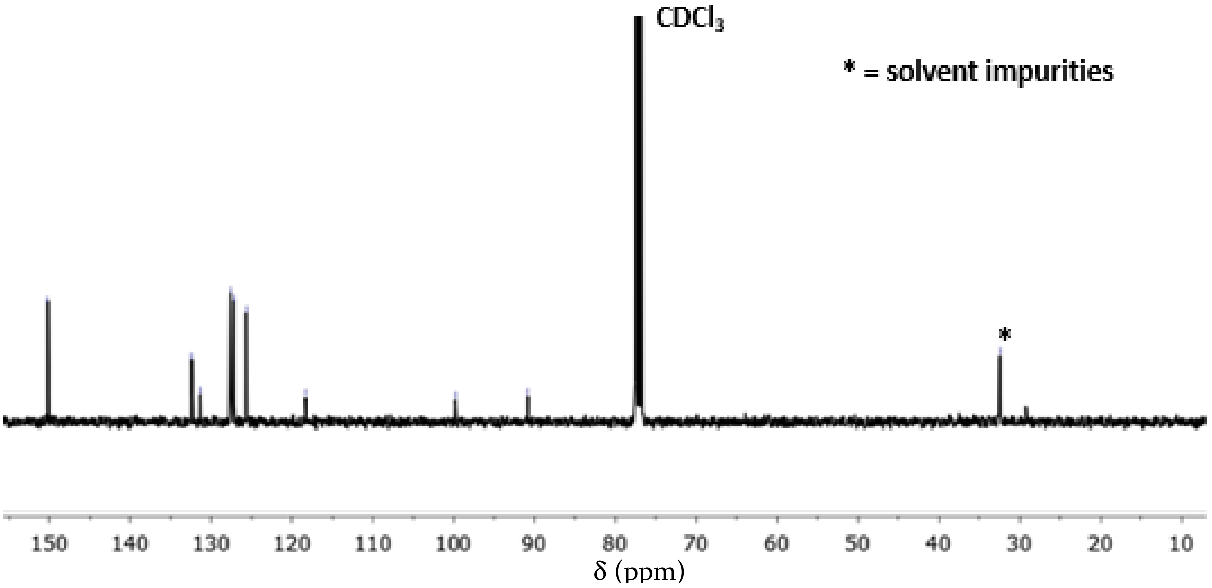

Figure S23: The ^{13}C{^{1}H} NMR spectrum of **3** in $CDCl_3$.

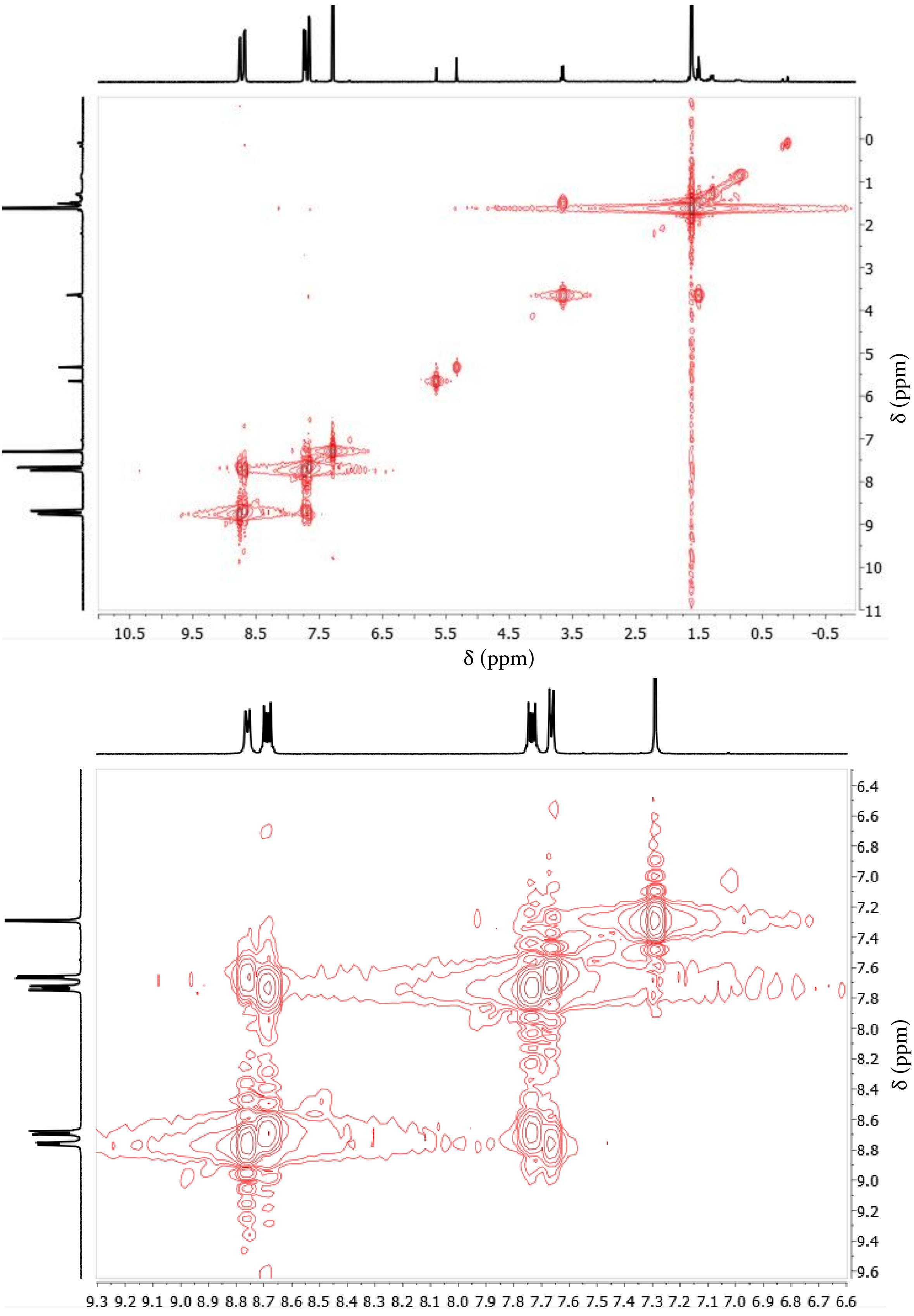

Figure S24: The ^{1}H-^{1}H COSY-NMR spectrum of **3** in $CDCl_3$. Full spectrum (top) and a zoomed region (bottom).

Molecule 3:Zn-TPP (1:1 ratio)

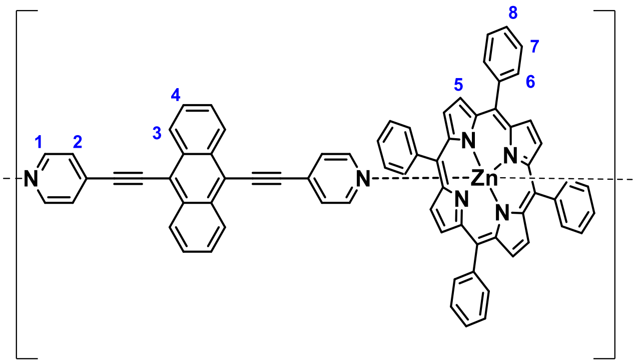

^{1}H-NMR ($CDCl_3$, 298 K, 400 MHz): δ = 8.88 (s, 8H, *H5*), 8.30-8.25 (m, 4H, *H3*), 8.21 (dd, $^3J_{H-H}$ = 7.2, $^4J_{H-H}$ = 1.2 Hz, 8H, *H6*), 7.78-7.68 (m, 12H, *H7*, *H8*), 7.50-7.44 (m, 4H, *H4*), 6.77 (br s, 4H, *H1*), 6.25 (br s, 4H, *H2*) ppm; **^{13}C{^{1}H}-NMR** ($CDCl_3$, 298 K, 100 MHz): δ =150.2 (Ar-C-H), 147.5, 143.6 (Ar-C-C), 141.9 (Ar-C-C), 134.8 (Ar-C-H), 132.1 (Ar-C-H), 131.8 (Ar-C-H), 131.2 (Ar-C-C), 127.3 (Ar-C-C), 126.9 (Ar-C-H), 126.4 (Ar-C-H), 125.0 (Ar-C-H), 120.8 (Ar-C-C), 118.0 (Ar-C-C), 99.2 (-C≡C-), 91.0 (-C≡C-) ppm; **MS** APCI: calcd. for $C_{72}H_{45}ZnN_6$ [M+H$^+$] 1057.2992; found 1057.3007.

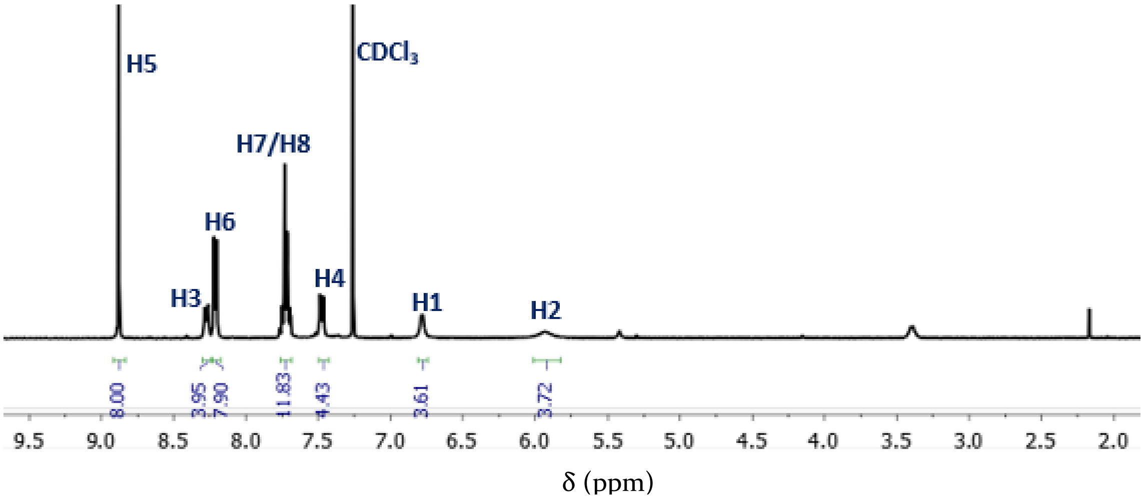

Figure S25: The ^{1}H NMR spectrum of a 1:1 ratio of **3:Zn-TPP**, in $CDCl_3$.

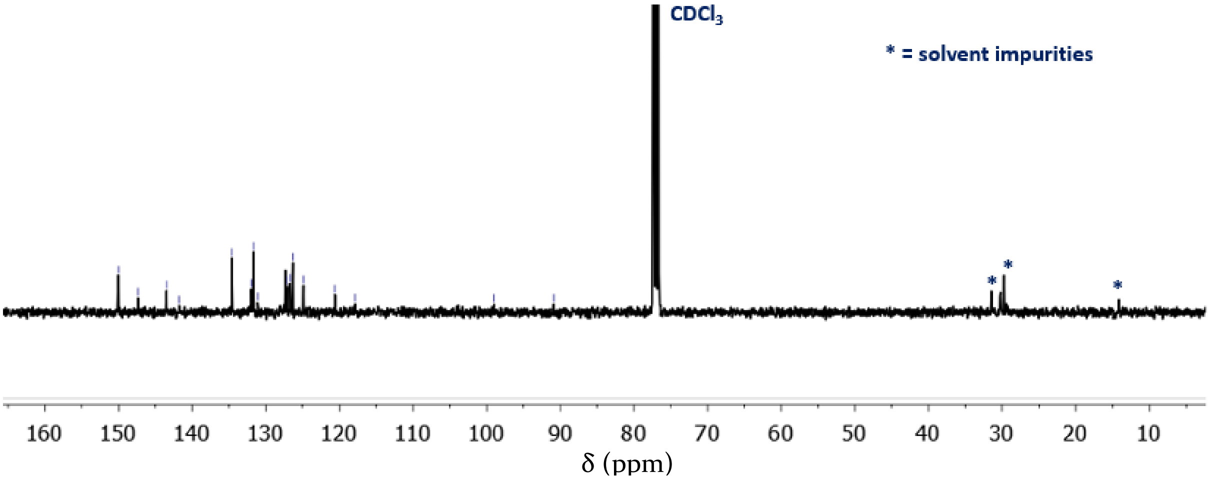

Figure S26: The ^{13}C{^{1}H} NMR spectrum of a 1:1 ratio of **3:Zn-TPP**, in $CDCl_3$.

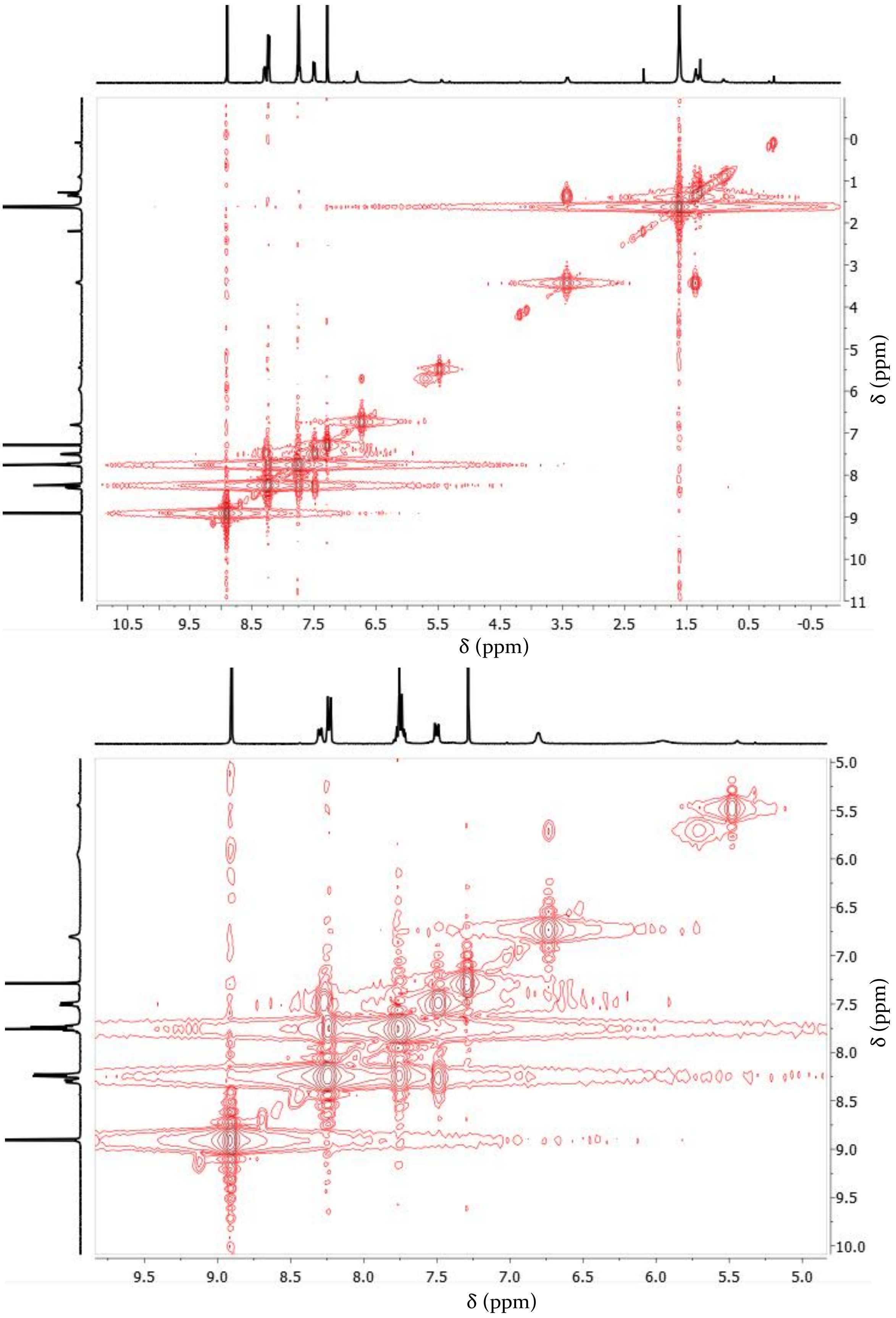

Figure S27: The ^{1}H-^{1}H COSY-NMR spectrum of a 1:1 ratio of **3:Zn-TPP** in $CDCl_3$. Full spectrum (top) and a zoomed region (bottom).

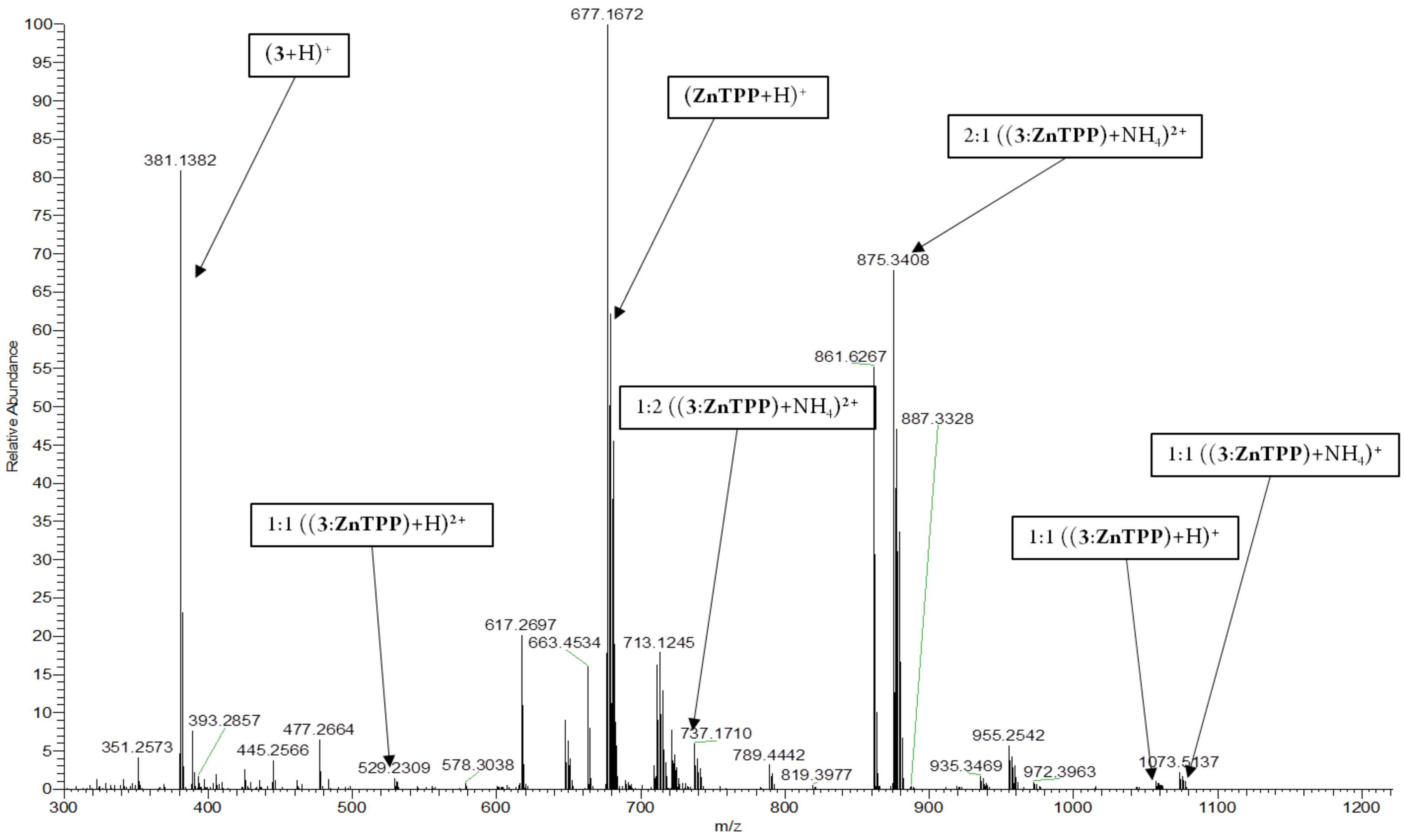

Figure S28: High resolution mass spectrometry of a 1:1 mixture of **3:Zn-TPP**, annotated to denote a range of identifiable peaks.

Molecule 3:Zn-TPP (2:1 ratio)

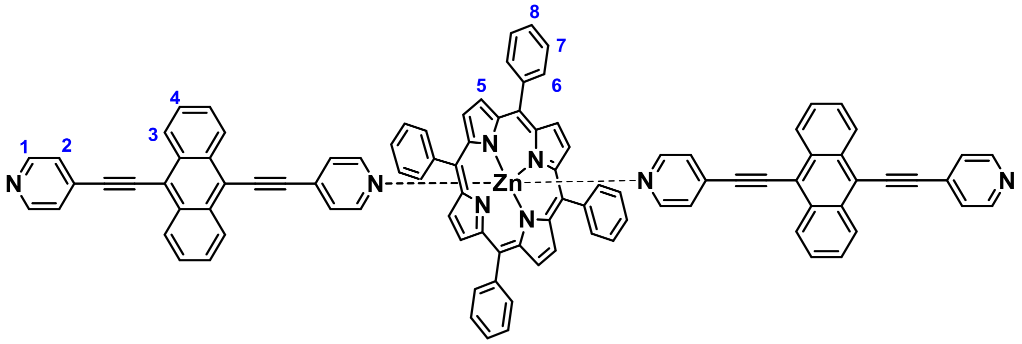

^{1}H-NMR ($CDCl_3$, 298 K, 400 MHz): δ = 8.88 (s, 8H, *H5*), 8.46-8.40 (m, 8H, *H3*), 8.22 (dd, $^{3}J_{H-H}$ = 7.2, 1.2 Hz, 8H, *H6*), 7.79-7.68 (m, 12H, *H7*, *H8*), 7.60-7.54 (m, 8H, *H4*), 7.14 (d, $^{3}J_{H-H}$ = 5.2 Hz, 8H, *H1*), 7.10 (br s, 8H, *H2*) ppm; **^{13}C{^{1}H}-NMR** ($CDCl_3$, 298 K, 100 MHz): δ = 150.2 (Ar-C-H), 148.2 (Ar-C-C), 143.6 (Ar-C-C), 134.8 (Ar-C-H), 132.2 (Ar-C-H), 131.8 (Ar-C-H), 131.3 (Ar-C-C), 127.5 (Ar-C-H), 127.3 (Ar-C-C), 126.9 (Ar-C-H), 126.4 (Ar-C-H), 125.2 (Ar-C-H), 120.8 (Ar-C-C), 118.1 (Ar-C-C), 99.3 (-C≡C-), 91.0 (-C≡C-) ppm; **MS** ES+: calcd. for $C_{100}H_{61}N_8Zn$ [M+H$^+$] 1439.4280; found 1439.4449.

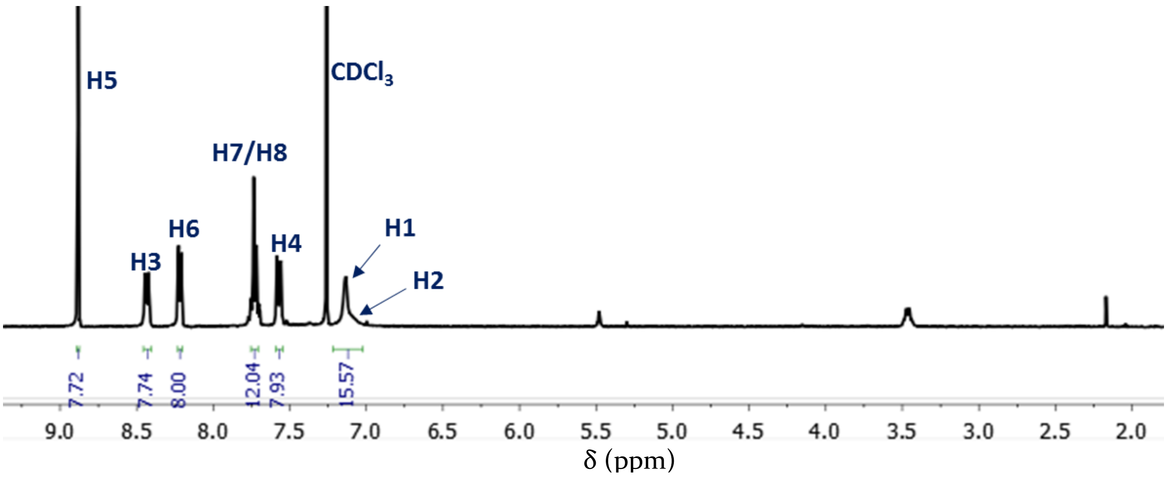

Figure S29: The ^{1}H NMR spectrum of a 2:1 ratio of **3**:**Zn-TPP**, in $CDCl_3$.

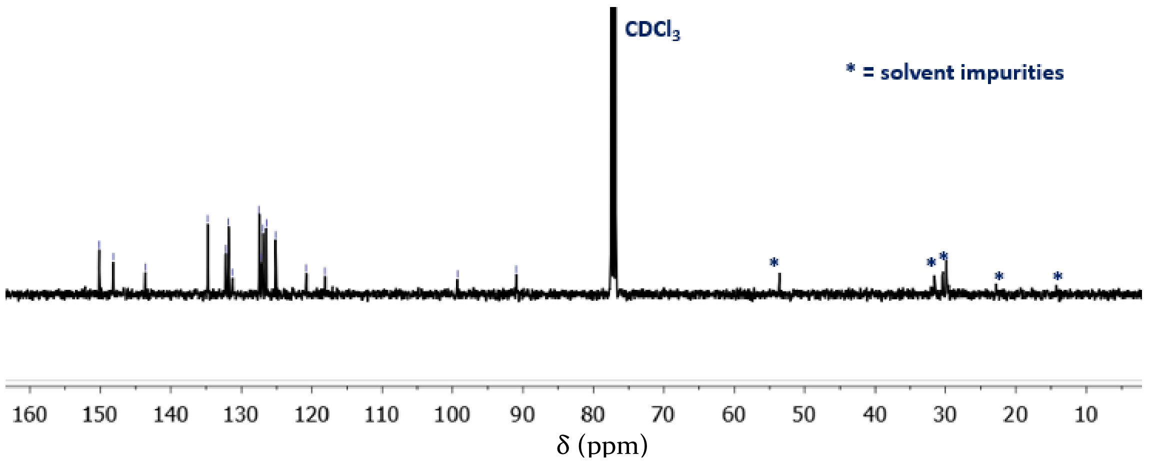

Figure S30: The ^{13}C{^{1}H} NMR spectrum of a 2:1 ratio of **3**:**Zn-TPP**, in $CDCl_3$.

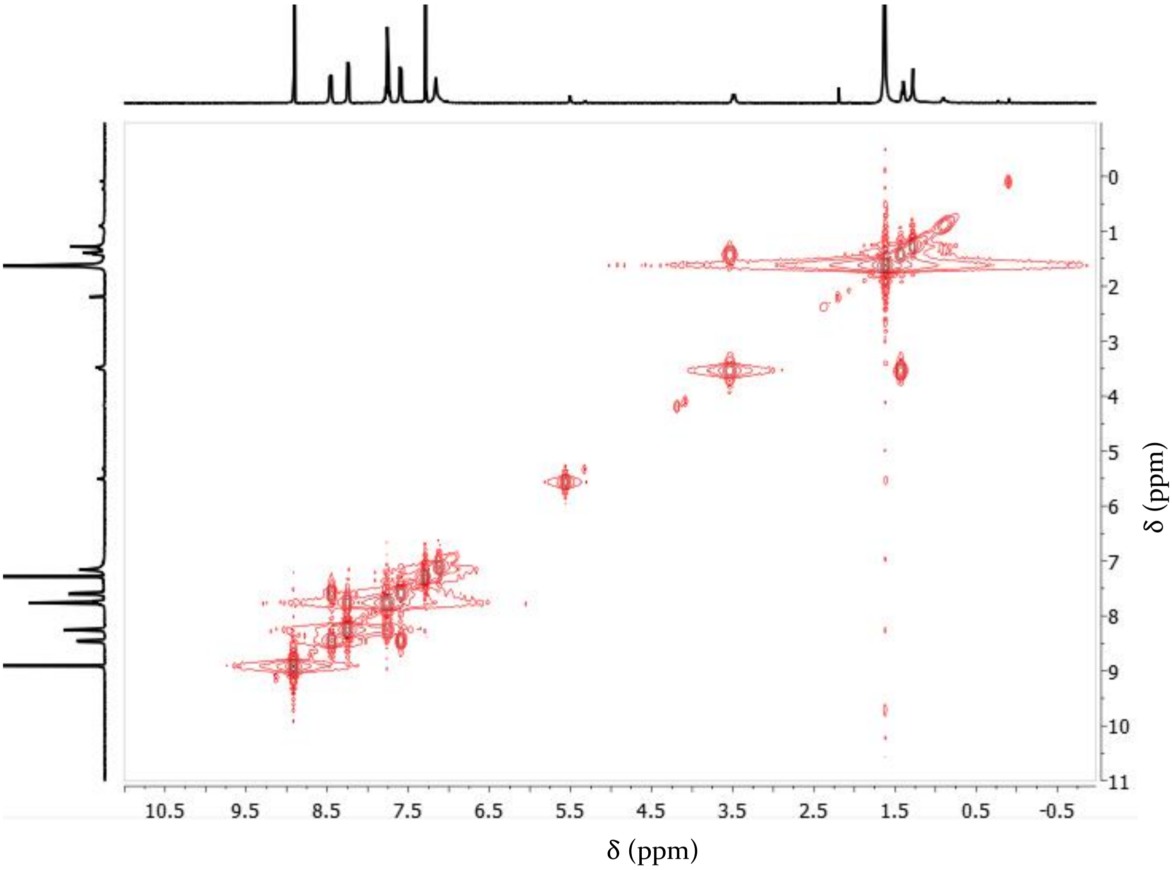

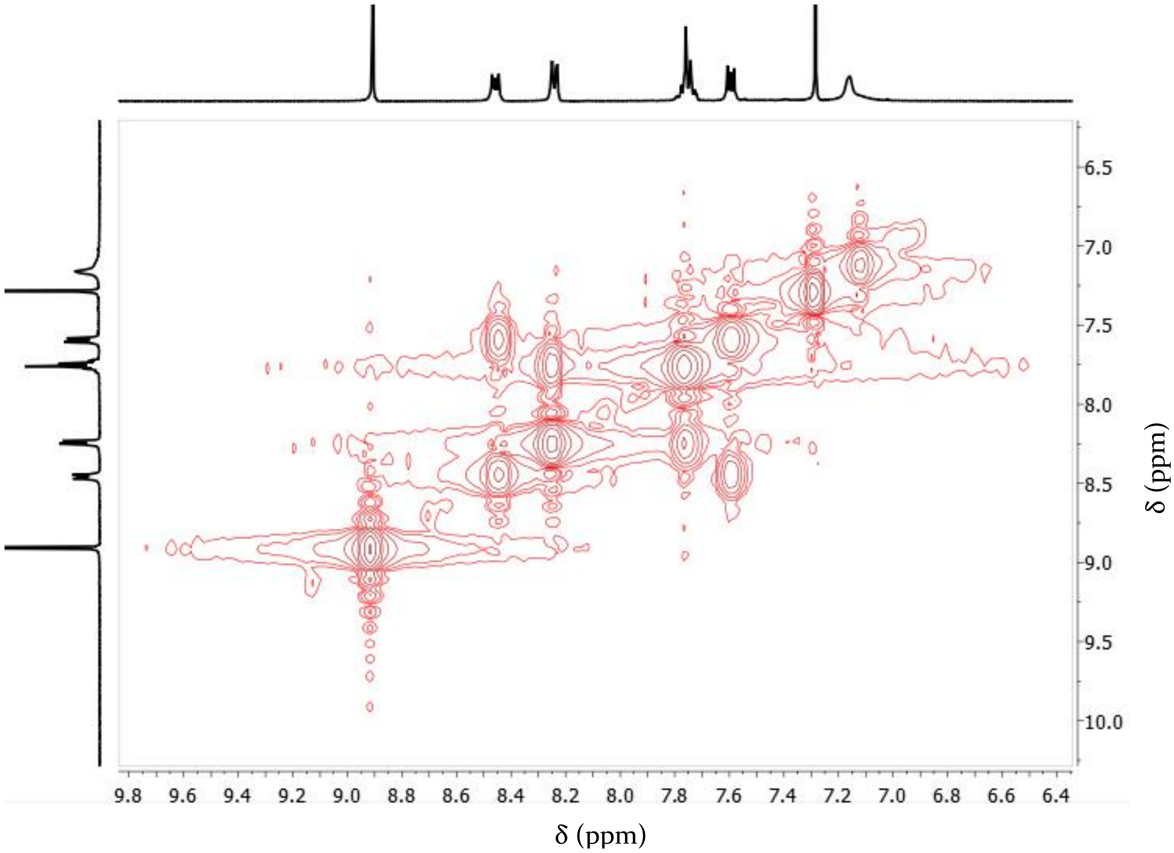

Figure S31: The ^{1}H-^{1}H COSY-NMR spectrum of a 2:1 ratio of **3:Zn-TPP** in $CDCl_3$. Full spectrum (top) and a zoomed region (bottom).

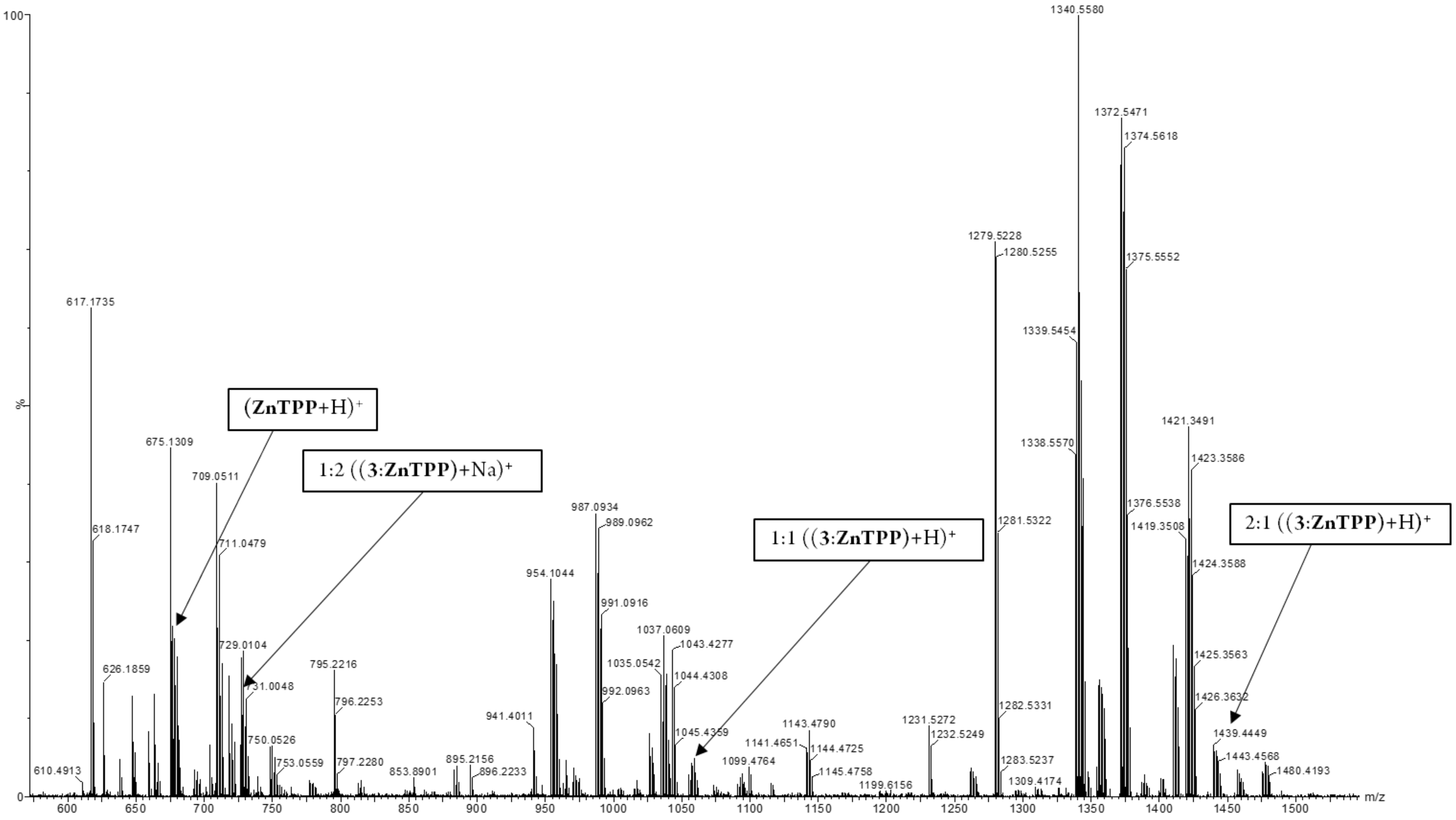

Figure S32: High resolution mass spectrometry of a 2:1 mixture of **3:Zn-TPP**.

1.5 NMR Comparisons

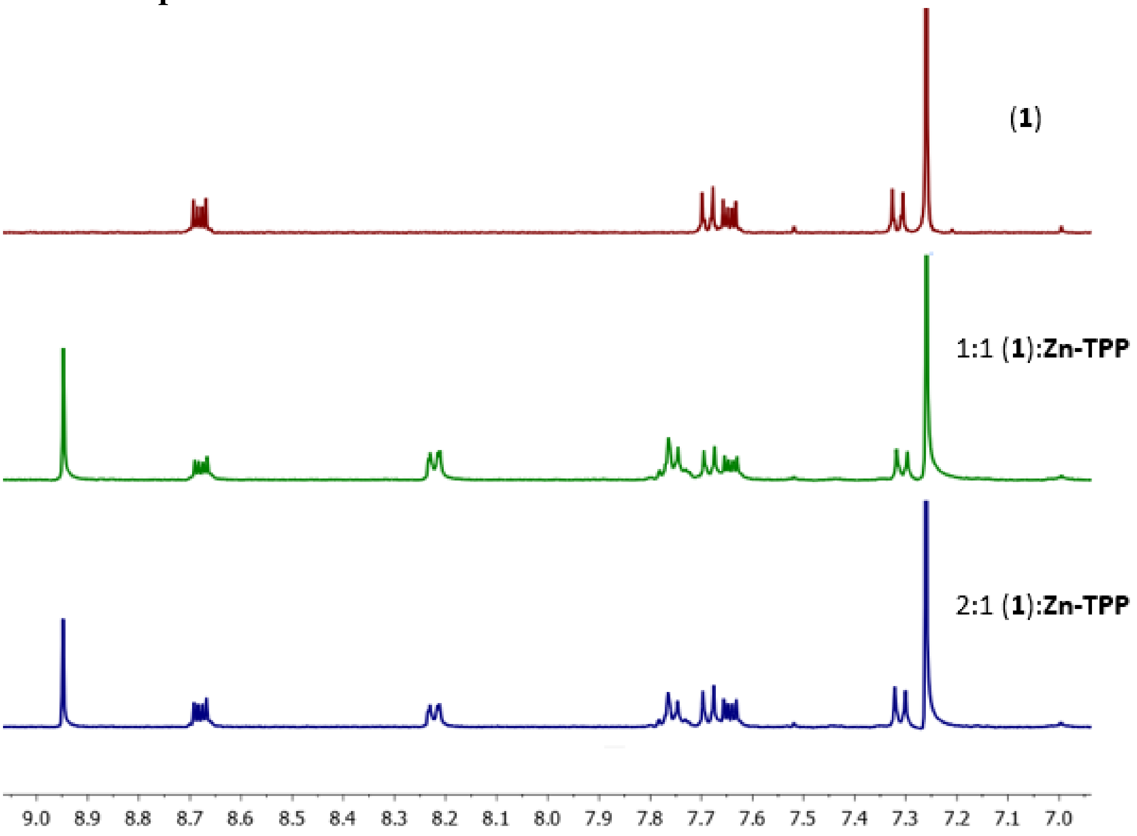

Figure S33: Stacked NMR spectra to illustrate changes in peak position and shape upon addition of **Zn-TPP** to solutions of molecule **1**.

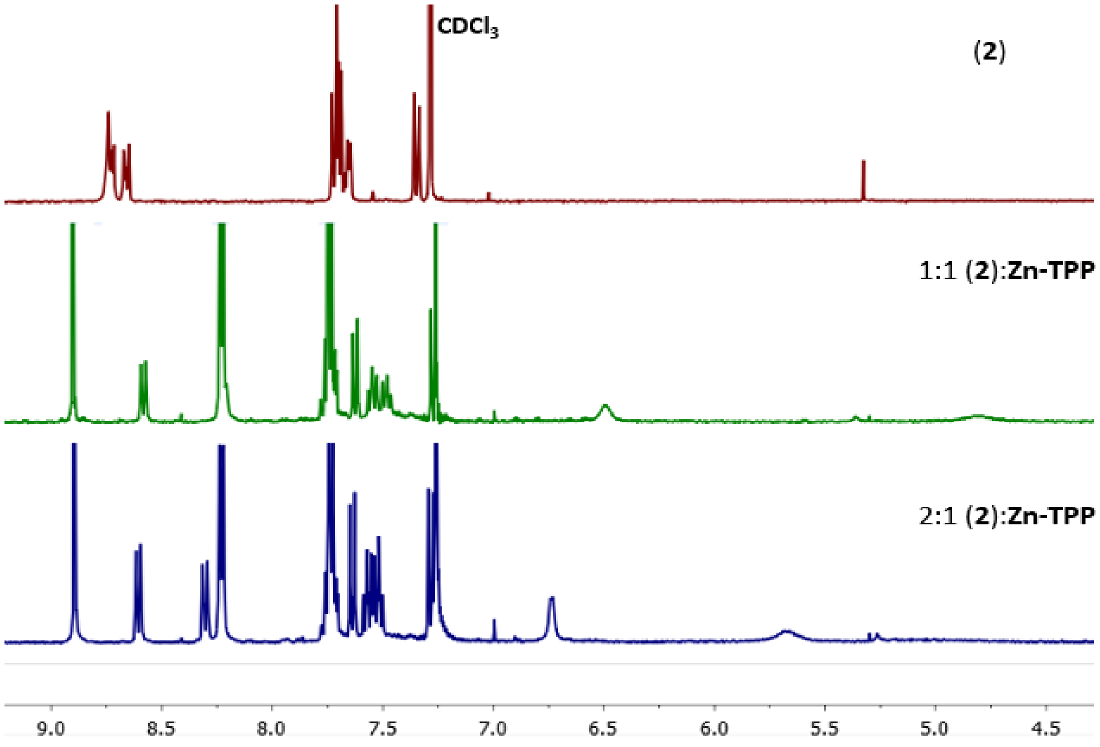

Figure S34: Stacked NMR spectra to illustrate changes in peak position and shape upon addition of **Zn-TPP** to solutions of molecule **2**.

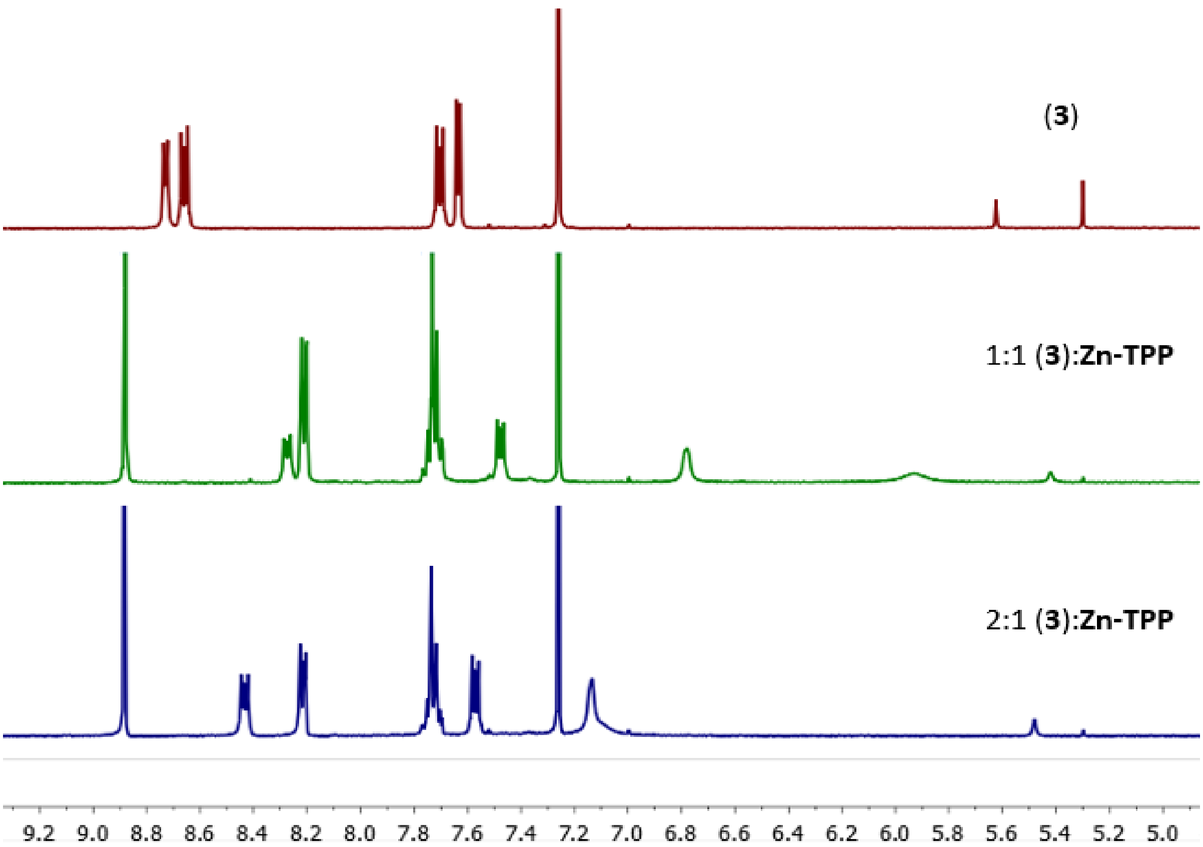

Figure S35: Stacked NMR spectra to illustrate changes in peak position and shape upon addition of **Zn-TPP** to solutions of molecule **3**.

1.6 Diffusion-Ordered NMR Spectroscopy (DOSY) Experiments

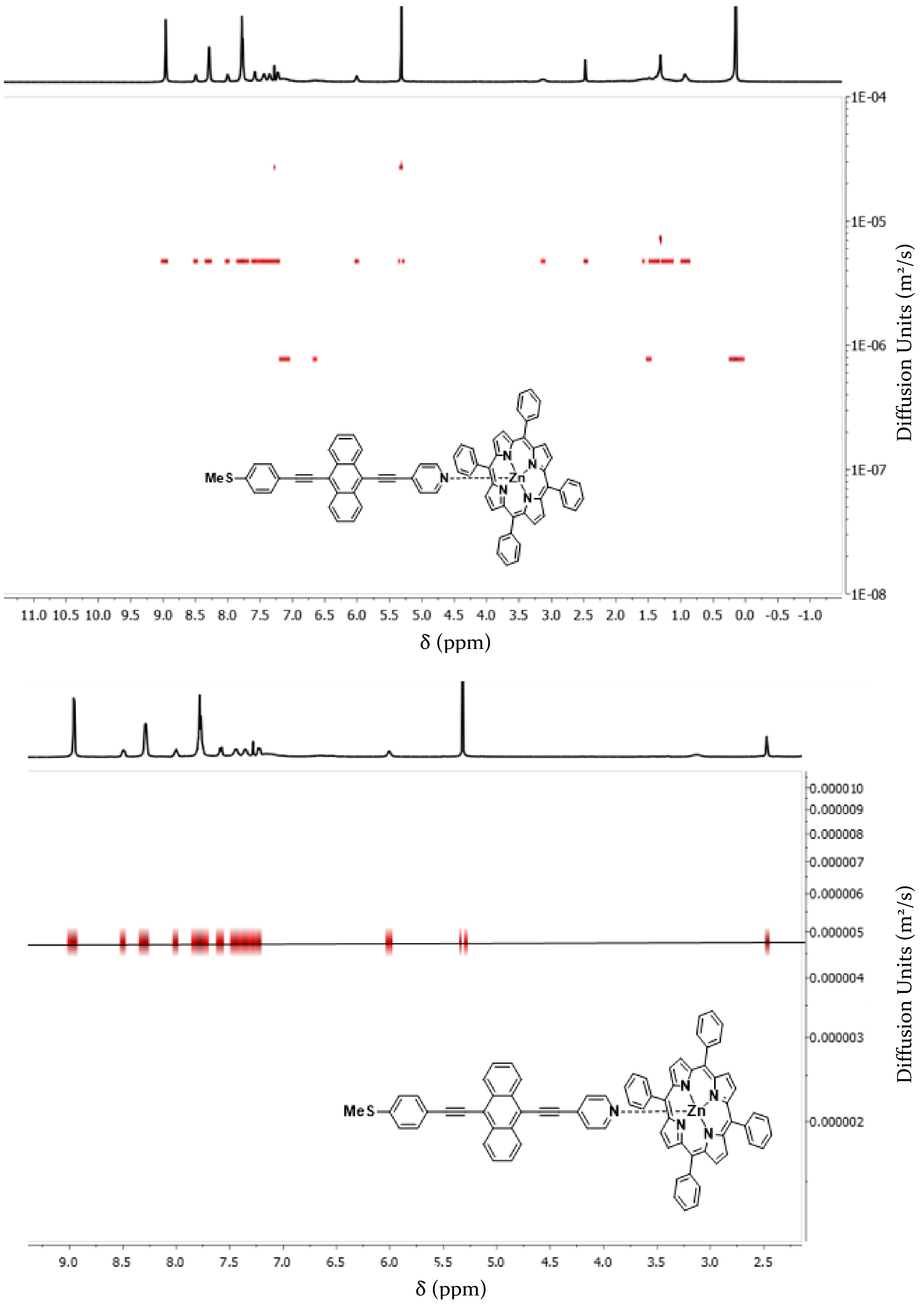

Figure S36: The DOSY-NMR spectrum of a 1:1 ratio of **2**:**Zn-TPP**, in $CDCl_3$. Full spectrum (top) and an expanded region (bottom).

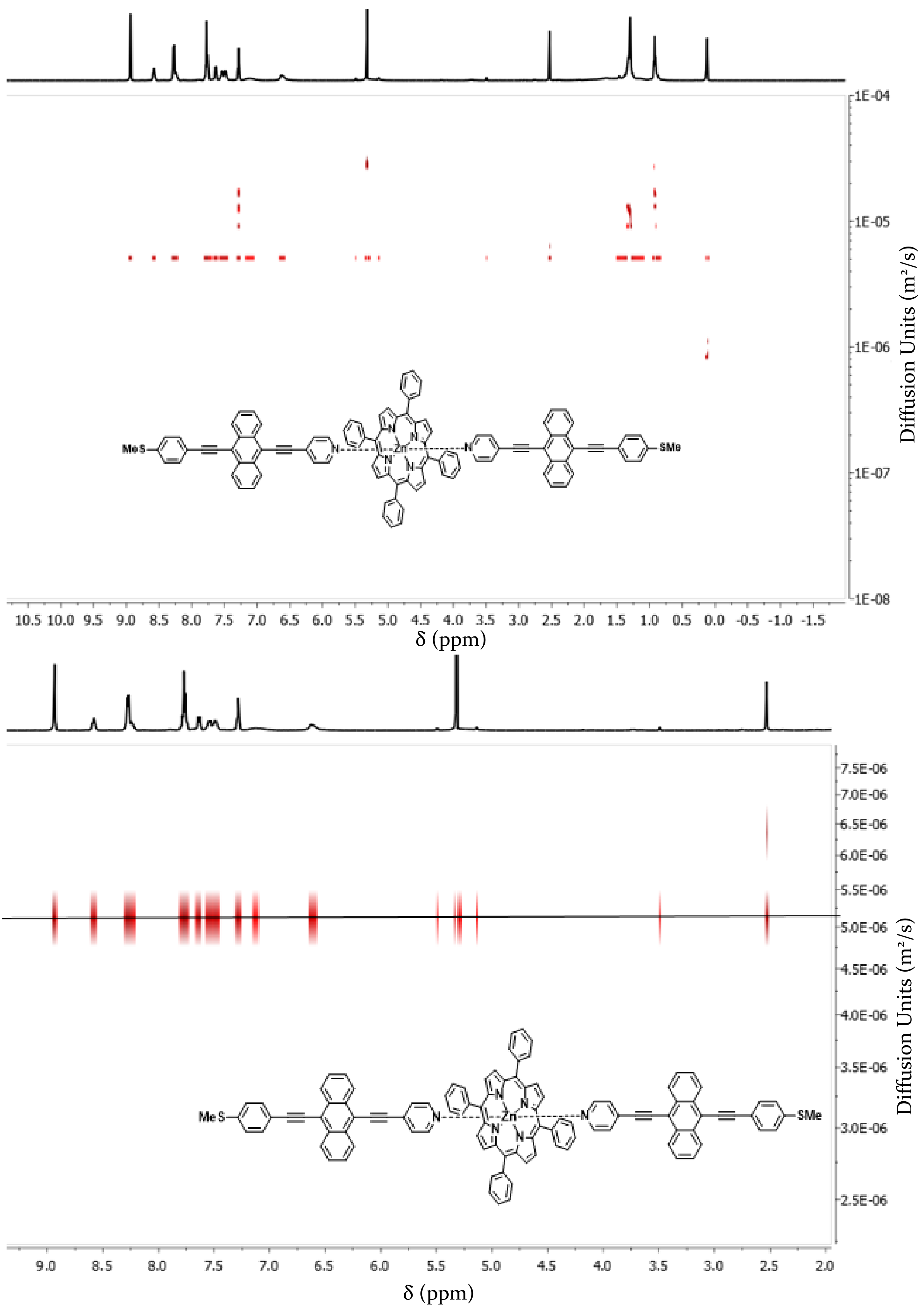

Figure S37: The DOSY-NMR spectrum of a 2:1 ratio of **2**:**Zn-TPP**, in $CDCl_3$. Full spectrum (top) and an expanded region (bottom).

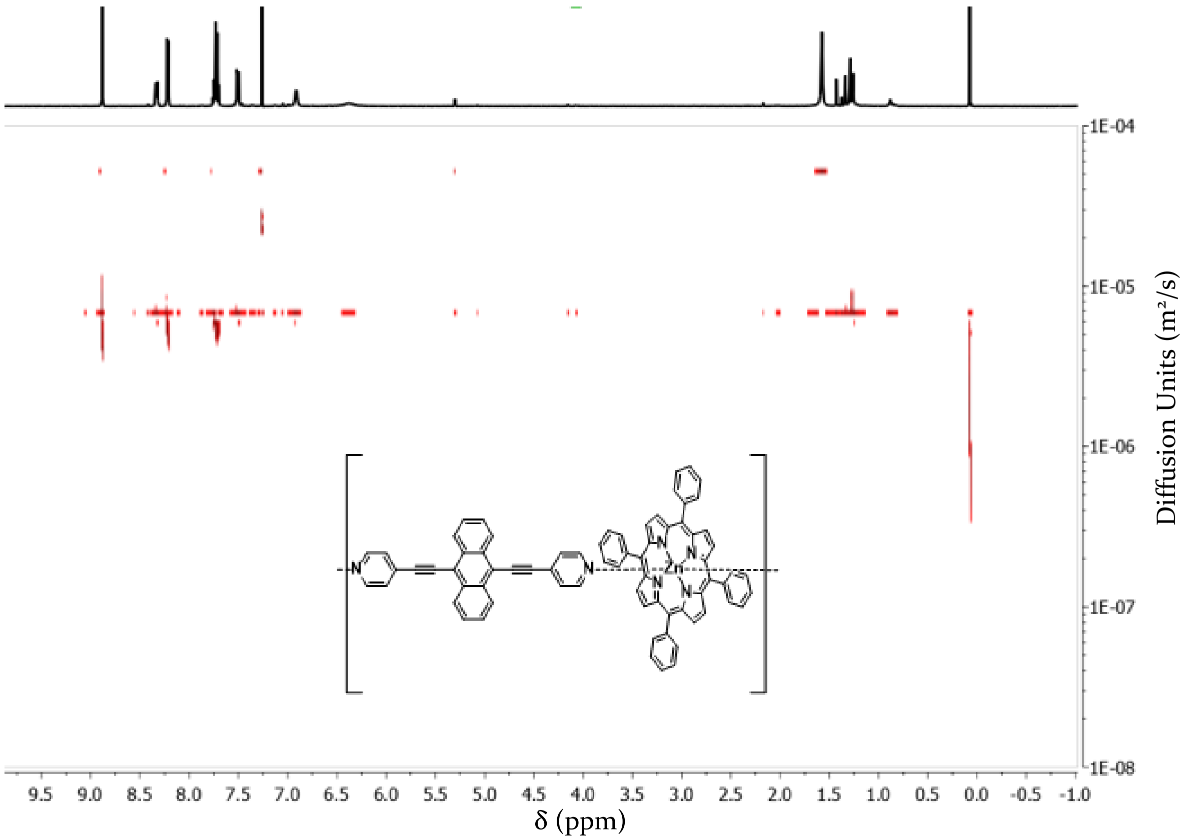

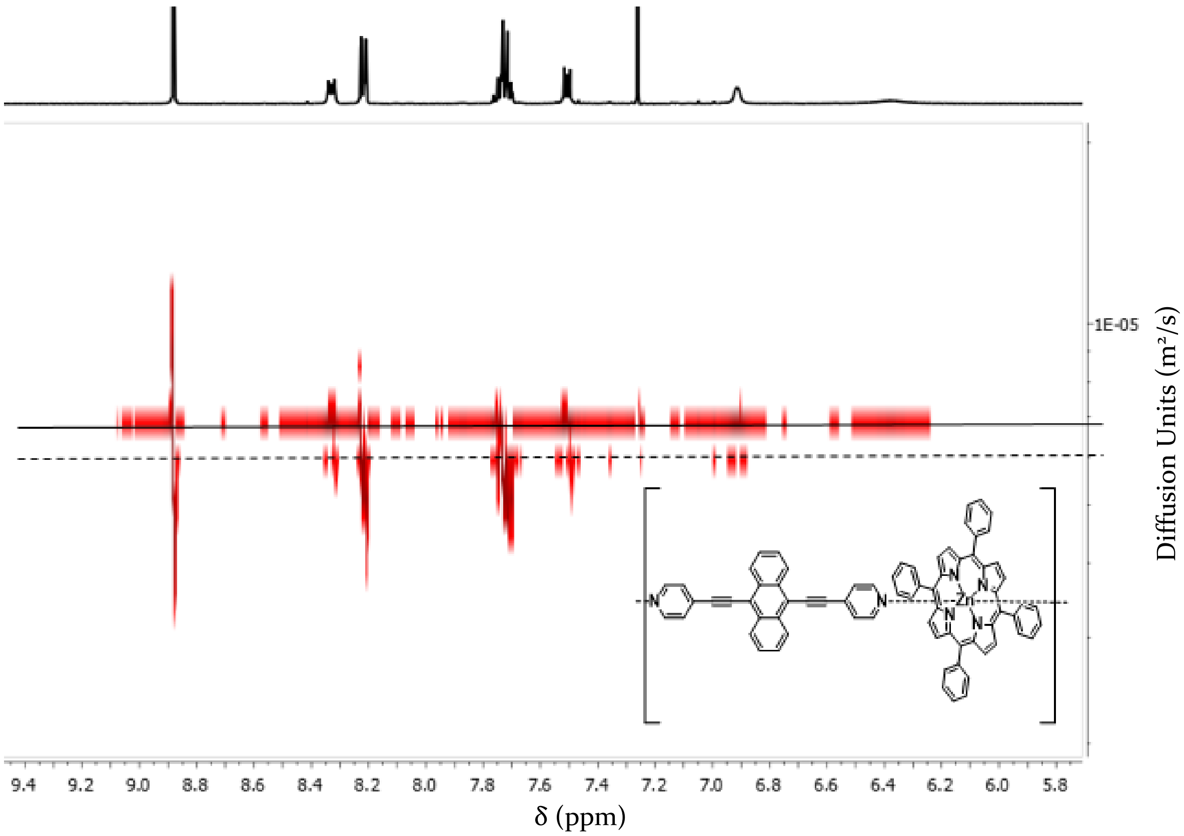

Figure S38: The DOSY-NMR spectrum of a 1:1 ratio of **3:Zn-TPP**, in $CDCl_3$. Full spectrum (top) and an expanded region (bottom).

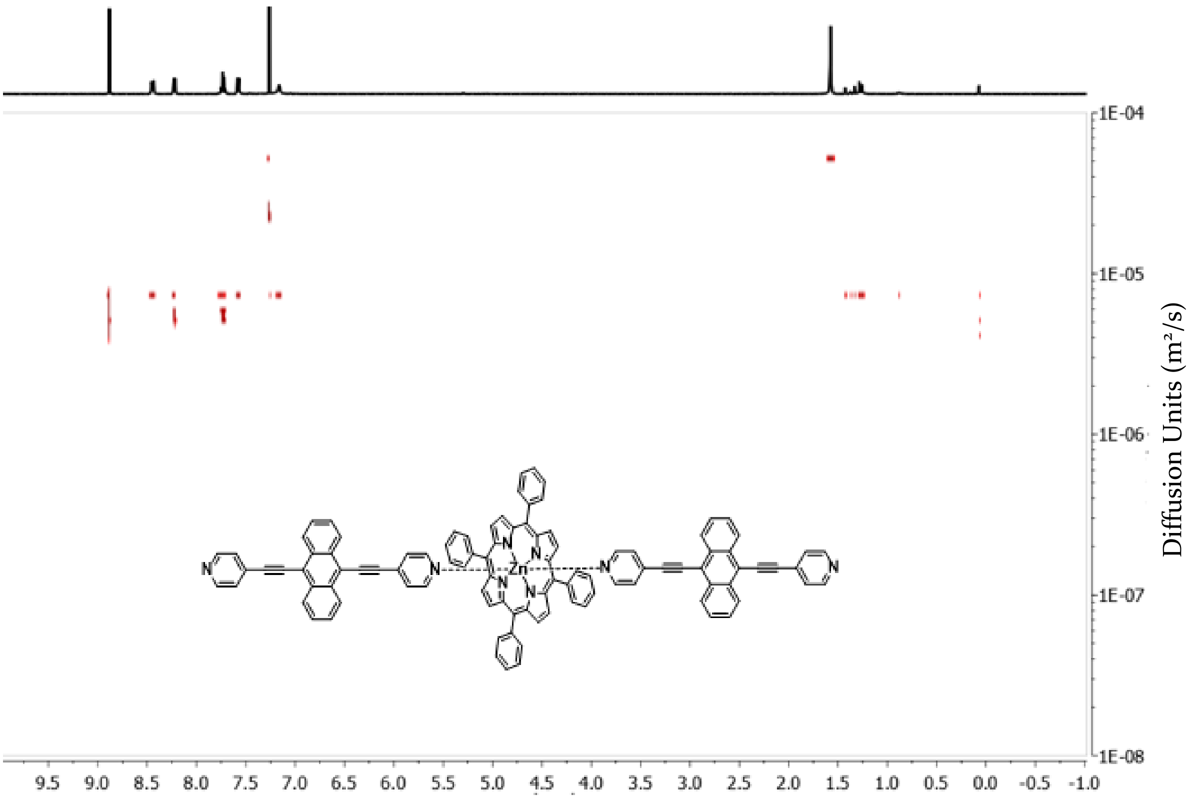

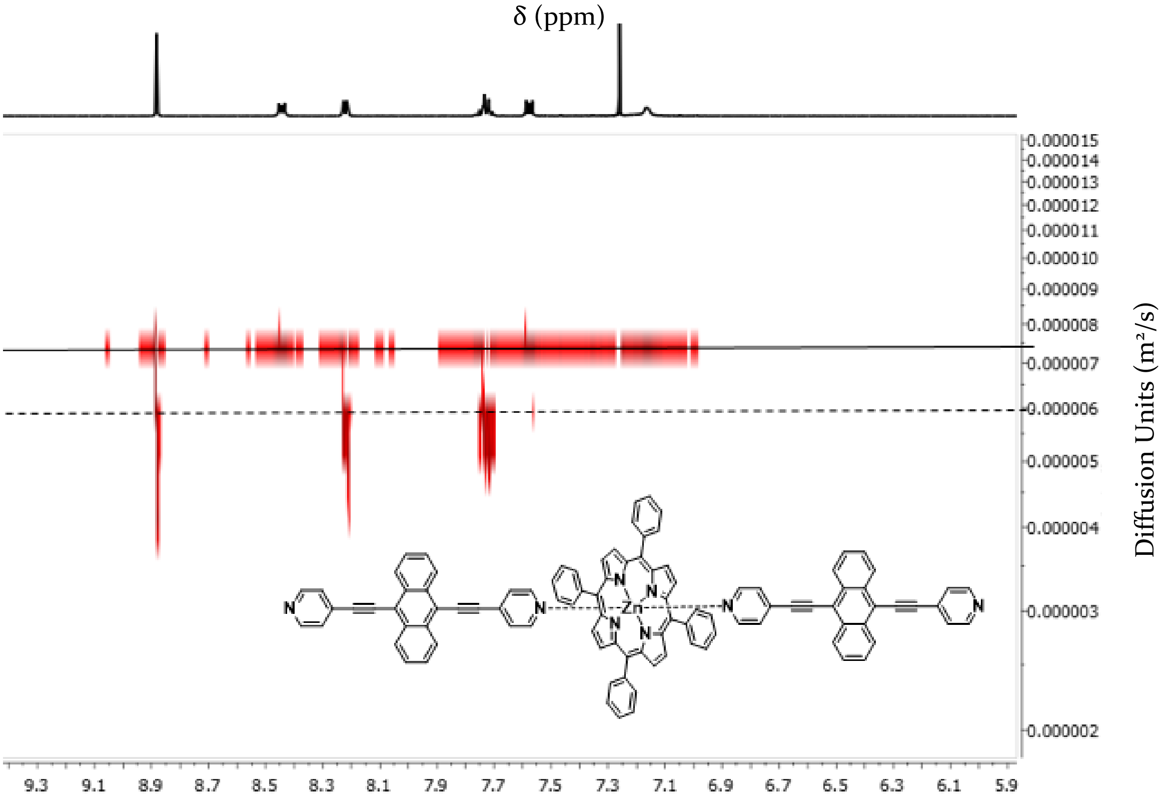

Figure S39: The DOSY-NMR spectrum of a 2:1 ratio of **3:Zn-TPP**, in $CDCl_3$. Full spectrum (top) and an expanded region (bottom).

2. DFT and Transport Calculations

In this section, geometries, electronic structures and transport properties of all junctions are presented. Since the main aim is to examine the change in transport properties when porphyrin and/or graphene are inserted, and since Au and Pt have similar work functions, the top contact will be chosen to be Au.

2.1 Optimised DFT Structures of Isolated Molecular-scale Structures

Using the density functional code SIESTA the optimum geometries of the isolated molecules were obtained by relaxing the molecules until all forces on the atoms were less than 0.01 eV / Å.[4,5] A double-zeta plus polarization orbital basis set, norm-conserving pseudopotentials, an energy cut-off of 250 Rydbergs defining the real space grid were used and the local density approximation (LDA) was chosen as the exchange correlation functional. We also computed results using GGA and found that the resulting transmission functions were comparable with those obtained using LDA. [6–8] The basic building blocks I-IV of this study are shown in Fig. S40:

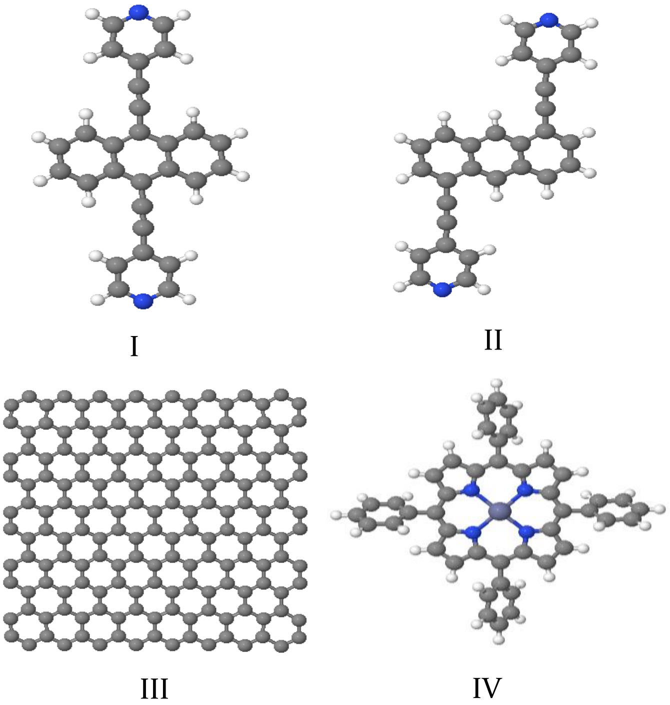

Figure S40: Simulated structures of I: Molecule **3** II: Molecule **4**, III: Graphene sheet, IV: **Zn-TPP**.

The following 8 molecular structures were assembled by combining the above components and then allowing the system to become fully relaxed;

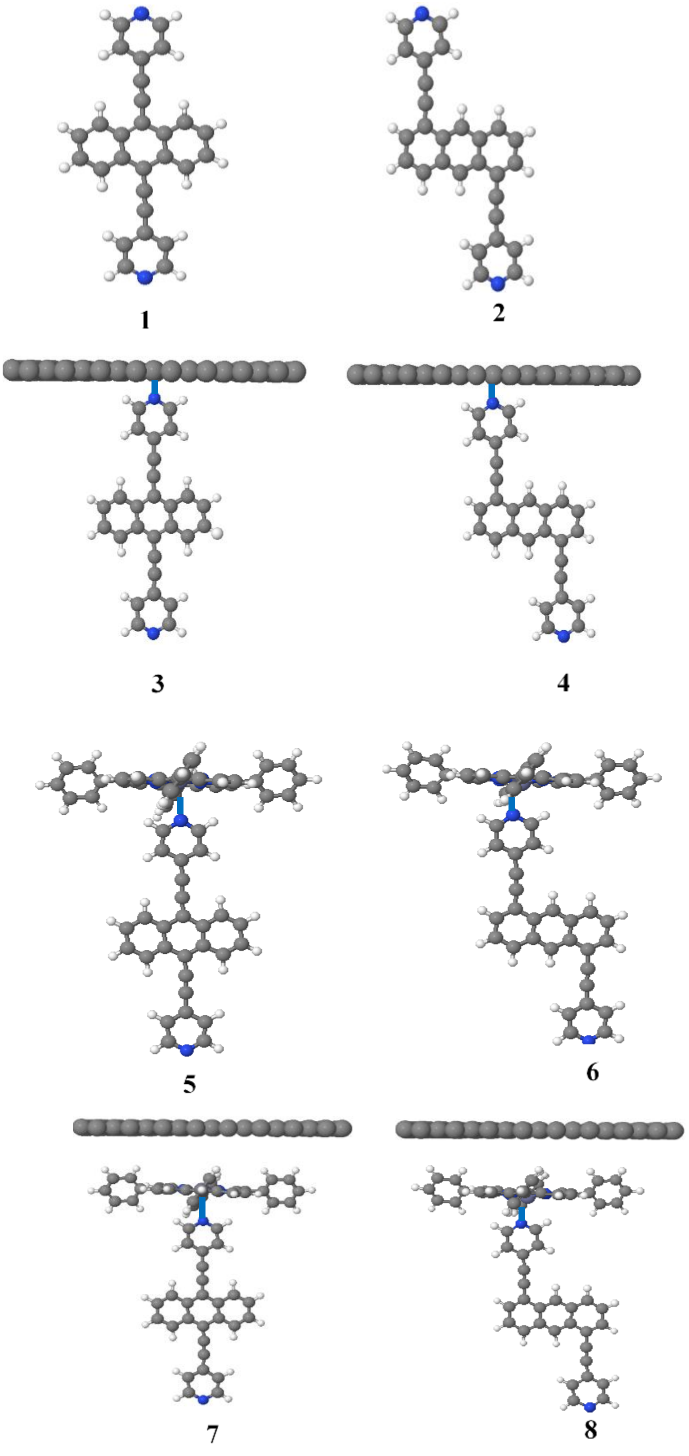

Figure S41: Fully relaxed isolated molecules. 1: molecule **3**, 2: molecule **4**, 3: Molecule **3** and a graphene sheet, 4: Molecule **4** and a graphene sheet, 5: Molecule **3** and **Zn-TPP**, 6: Molecule **4** and **Zn-TPP**, 7: Molecule **3**, **Zn-TPP** and a graphene sheet, 8: Molecule **4**, **Zn-TPP** and a graphene sheet. Key: C=grey, H= white, light blue=Zn, dark blue= N.

2.2 Frontier orbitals

The plots below show isosurfaces of the HOMO, LUMO, HOMO-1 and LUMO+1 of isolated molecules **3** and **4**.

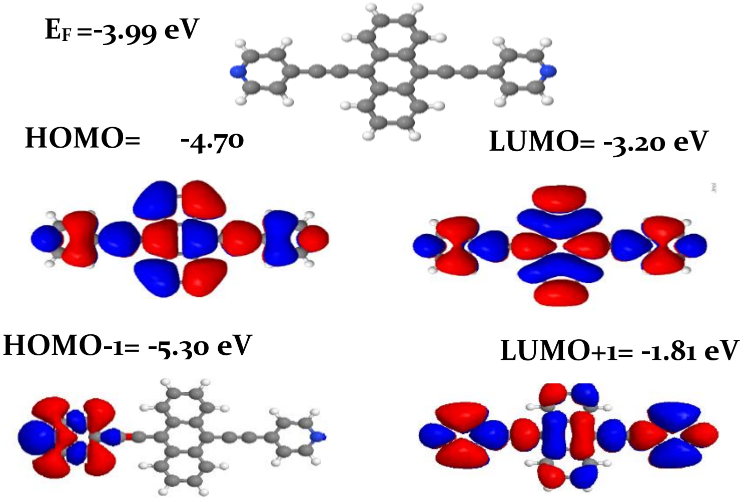

Figure S42: Wave function for **3**. Top panel: fully optimised geometry of **3**. Lower panel: HOMO, LUMO, HOMO-1, LUMO+1 of molecule **3**, along with their energies.

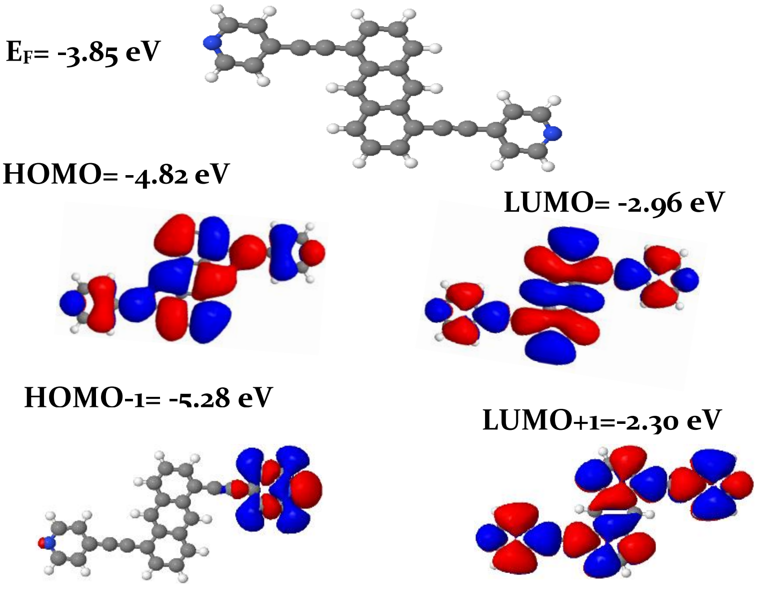

Figure S43: Wave function for **4**. Top panel: fully optimised geometry of **4**. Lower panel: HOMO, LUMO, HOMO-1, LUMO+1 of molecule **4** along with their energies.

2.3 Binding energies

To calculate the optimum binding distance between any two components, we used DFT and the counterpoise method, which removes basis set superposition errors (BSSE). The binding distance d is defined as the distance between compound 1 and compound 2. Here, compound 1 is defined as entity A and compound 2 as entity B. The ground state energy of the total system is calculated using SIESTA and is denoted $\boldsymbol{E_{AB}^{AB}}$. The energy of each entity is then calculated in a fixed basis, which is achieved using ghost atoms in SIESTA. Hence, the energy of the individual 1 in the presence of the fixed basis is defined as $\boldsymbol{E_{A}^{AB}}$ and for the gold as $\boldsymbol{E_{B}^{AB}}$**.** The binding energy is then calculated using the following equation:

$$\boldsymbol{Binding\ Energy = E_{AB}^{AB} - E_{A}^{AB} - E_{B}^{AB}} \qquad \textbf{(S1)}$$

2.3.1 Binding energy of two components

In this section, four binding energies are calculated. B1 to find the optimum distance between the anthracene and graphene sheet, B2 to find the optimum distance between the anthracene and porphyrin (**Zn-TPP**, Zn-N), B3 between the anthracene porphyrin (**Zn-TPP**) molecule, and graphene sheet. B4 between the anthracene and porphyrin (**Zn-TPP**, Zn-SMe), Figure S44 illustrates the four structures.

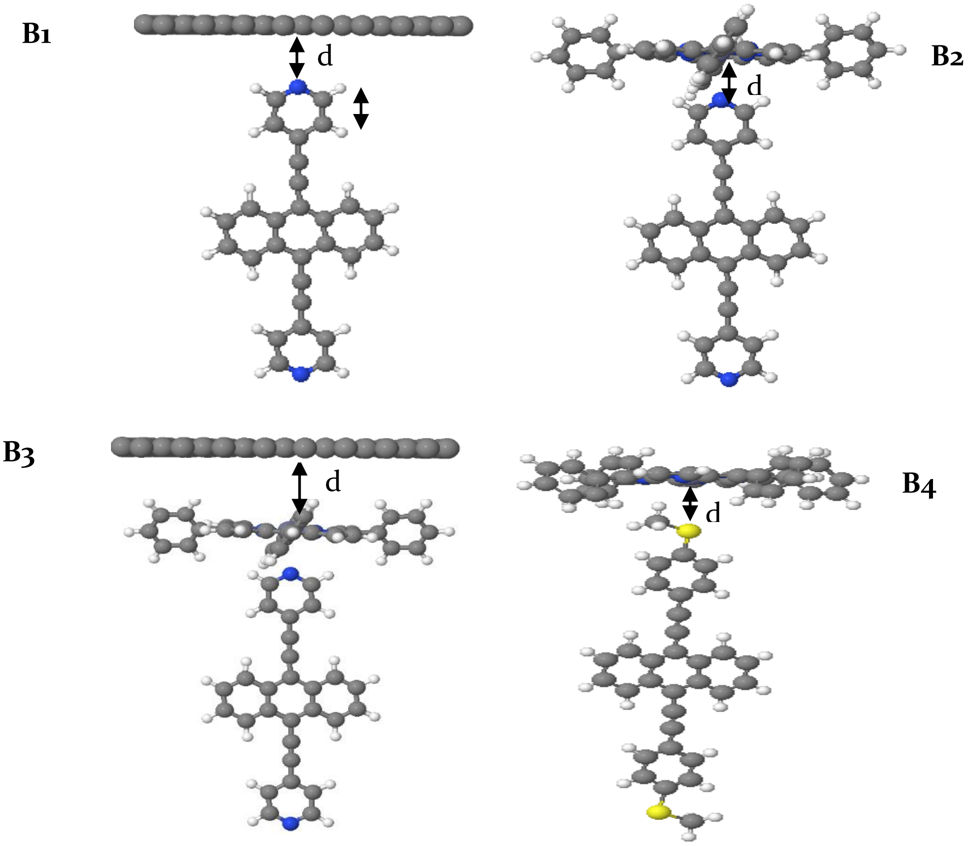

Figure S44: B1 represents molecule **3** binding to a graphene sheet.,B2 represents molecule **3** binding to **Zn-TPP**, B3 represents molecule **3** binding to **Zn-TPP**, bound to a graphene sheet and B4 represents molecule **1** binding to **Zn-TPP**.

Figure S45 (B1) shows that a value of d = 3.0 Å gives the optimum distance between the anthracene and graphene sheet, with a binding energy of approximately 0.15 eV. For (B2) the anthracene and porphyrin (**Zn-TPP**), d = 2.3 Å (Zn-N), with a binding energy of approximately 0.5 eV. After binding this molecule to a graphene sheet, the optimum distance for (B3) was found to be d = 4.0 Å (Zn- graphene sheet) with a binding energy of approximately 0.15 eV.

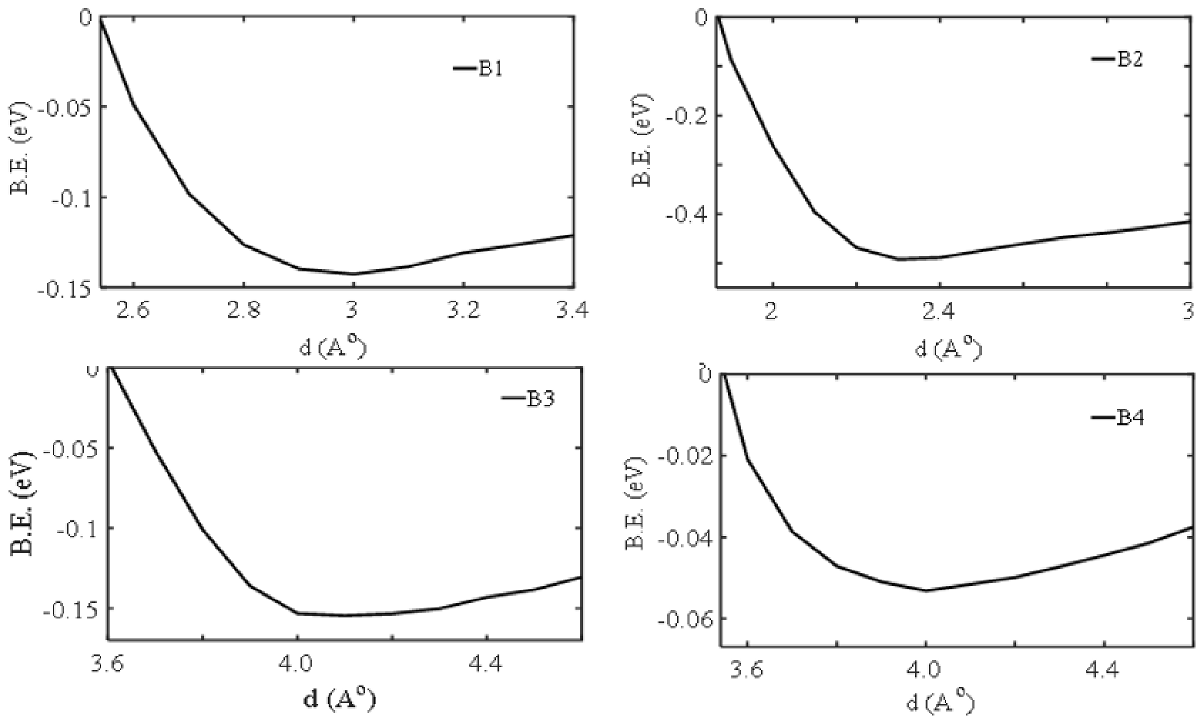

Figure S45: Binding energy plots of B1 (top-left), B2 (top-right), B3 (bottom-left) and B4 (bottom-right). For B1: d = 3.0 Å and B.E= 0.15 eV gives the optimum distance between molecule **3** and a graphene sheet. For B2, molecule **3** and a graphene sheet, d = 2.3 Å (Zn-N) and B.E= 0.5 eV. For B3, molecule **3** binding to porphyrin (**Zn-TPP**) and bound to a graphene sheet, d = 4.0 Å (Zn- graphene sheet) and B.E= 0.15 eV. For B4, molecule **1** binding to porphyrin (**Zn-TPP**) d = 4.0 Å (Zn-SMe) and B.E= 0.05 eV.

2.3.2 Binding energy of molecules on Au

After obtaining the optimum structures of the molecules of study, shown in Fig. S45, the next step was to find the optimum distance between the Au electrode and the molecules.

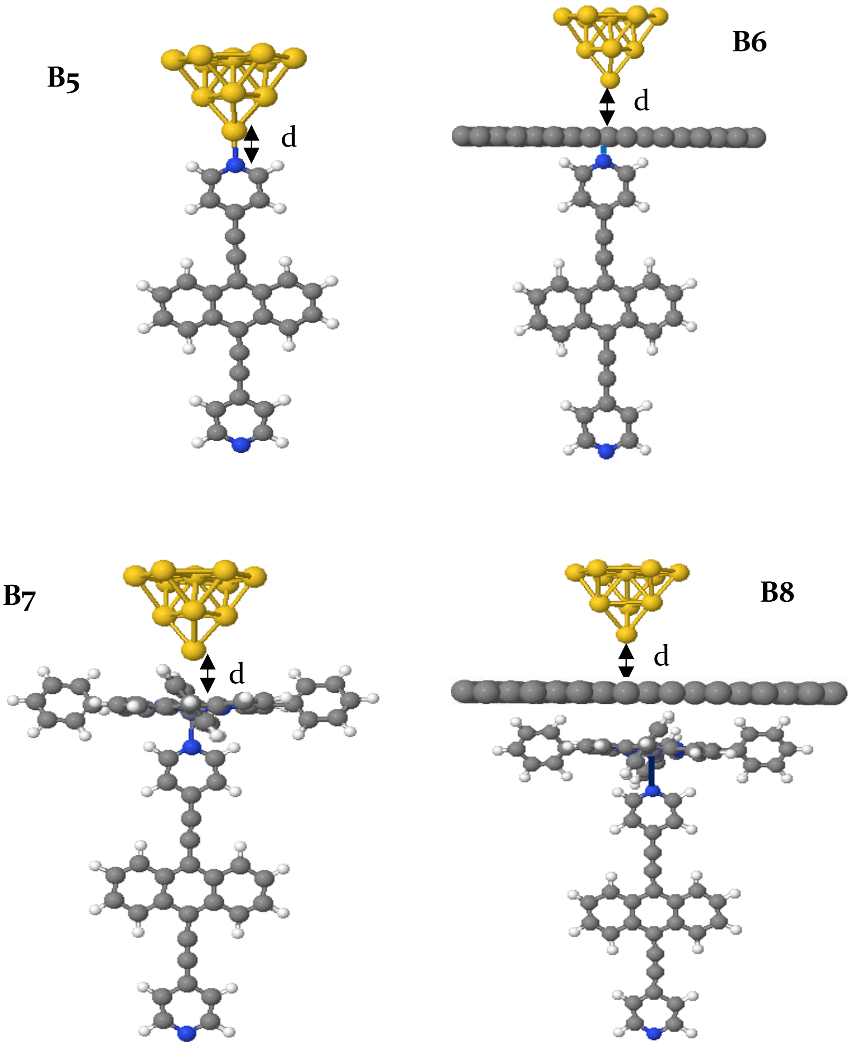

Figure S46: Molecule configuration at the Au lead interface. B5 molecule **3**, B6 molecule **3** and a graphene sheet, B7 molecule **3** and **Zn-TPP**, B8 molecule 3, **Zn-TPP** and a graphene sheet.

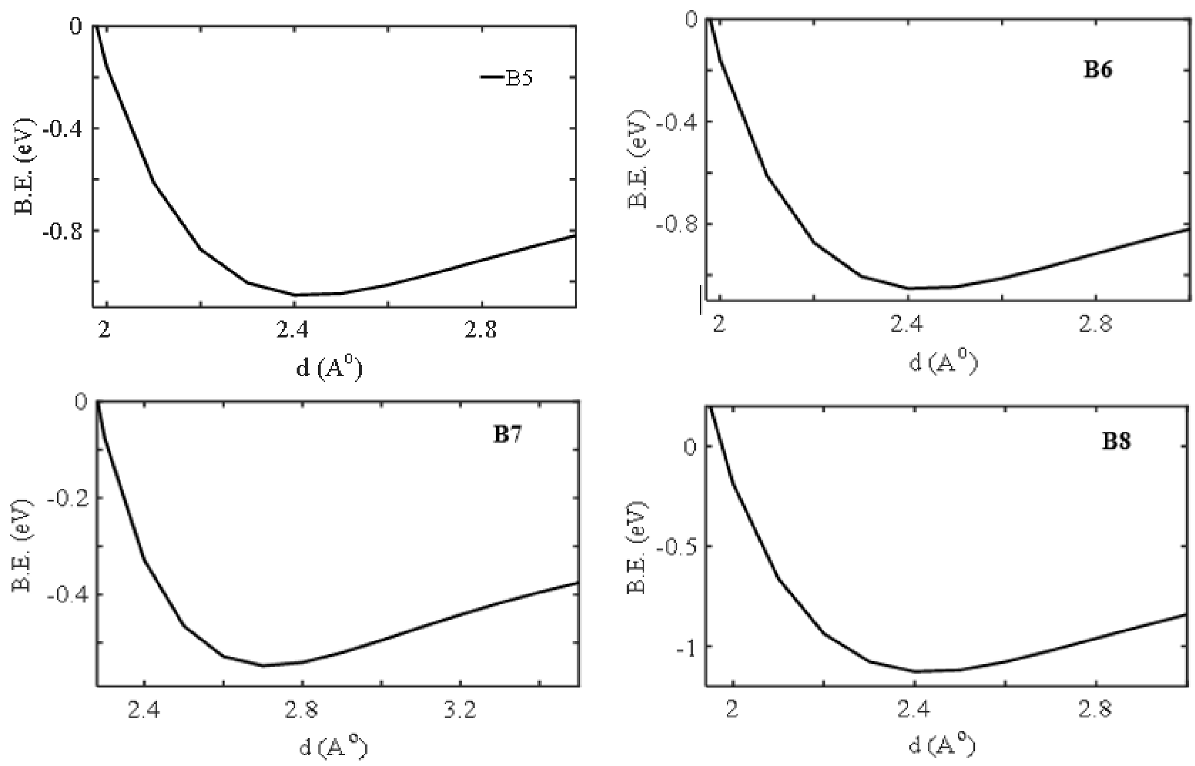

Figure S47: Binding energy plots of B5 (top-left), B6 (top-right), B7 (bottom-left) and B8 (bottom-right). For, B5 d = 2.3 Å and B.E= 0.4 eV gives the optimum distance between molecule **3** and Au. For B6, molecule **3** bound to porphyrin, bound to a graphene sheet d = 2.4 Å (graphene-Au) and B.E= 1.0 eV. For B7, porphyrin (**Zn-TPP**) and a graphene sheet, d = 2.7 Å (Zn- Au) and B.E= 0.5 eV. For B8, molecule **3**, porphyrin and a graphene sheet, d = 2.4 Å (graphene-Au) and B.E= 1 eV.

B4 was added to this study to investigate whether porphyrin (**Zn-TPP**) binds to anthracene with SMe anchor. Comparing B2 (anthracene Py) binding energy curve against B4 (anthracene SMe) curves, shows that anthracene Py binds 10 times more strongly than anthracene SMe (0.5 and 0.05 eV), see B4 and B2 of Fig. S45.

Table S1: Summarises the binding energy calculations for the eight cases.

Compound	B.E (eV)	d (Å)
**B1**	0.15	3.0
**B2**	0.50	2.3
**B3**	0.15	4.0
**B4**	0.05	4.0
**B5**	0.40	2.3
**B6**	1.00	2.4
**B7**	0.50	2.7
**B8**	1.00	2.4

2.4 The tilt angle (θ)

In this section, we determine the tilt angle *θ* of each compound on a gold substrate, which corresponds to the experimentally measured most-probable break-off distance. In previous work we demonstrated how the tilt angle varies between single molecules and SAMs.[9-13] Table S2 shows each compound for a range of tilt angles. Break-off distance values suggest that the eight compounds tilt with angle θ ranging from 45° to 50°. Note, that experimentally, the Au/3 and Au/3/P, and Au/4/G and Au/4/P/G were contacted by Pt probes, in all cases the film thickness and roughness is identical whether contacted by Pt probe or graphene-coated Pt probe.

Table S2: Experimental break-off distance and equivalent tilt angle (θ).

Junction	Experimental film thickness (nm)	Experimental film roughness (nm)	Equivalent experimental tilt angle (θ)	Equivalent theoretical tilt angle (θ)
Au/3/Au	1.23	0.2	38°-55°	38°-55°
Au/4/Au	1.14	0.2	42°-58°	42°-58°
Au/3/P/Au	1.81	0.4	38°-55°	38°-55°
Au/4/P/Au	1.73	0.3	42°-58°	42°-58°
Au/3/G/Au	1.23	0.2	38°-55°	38°-55°
Au/4/G/Au	1.14	0.2	42°-58°	42°-58°
Au/3/P/G/Au	1.81	0.4	38°-55°	38°-55°
Au/4/P/G/Au	1.73	0.3	42°-58°	42°-58°

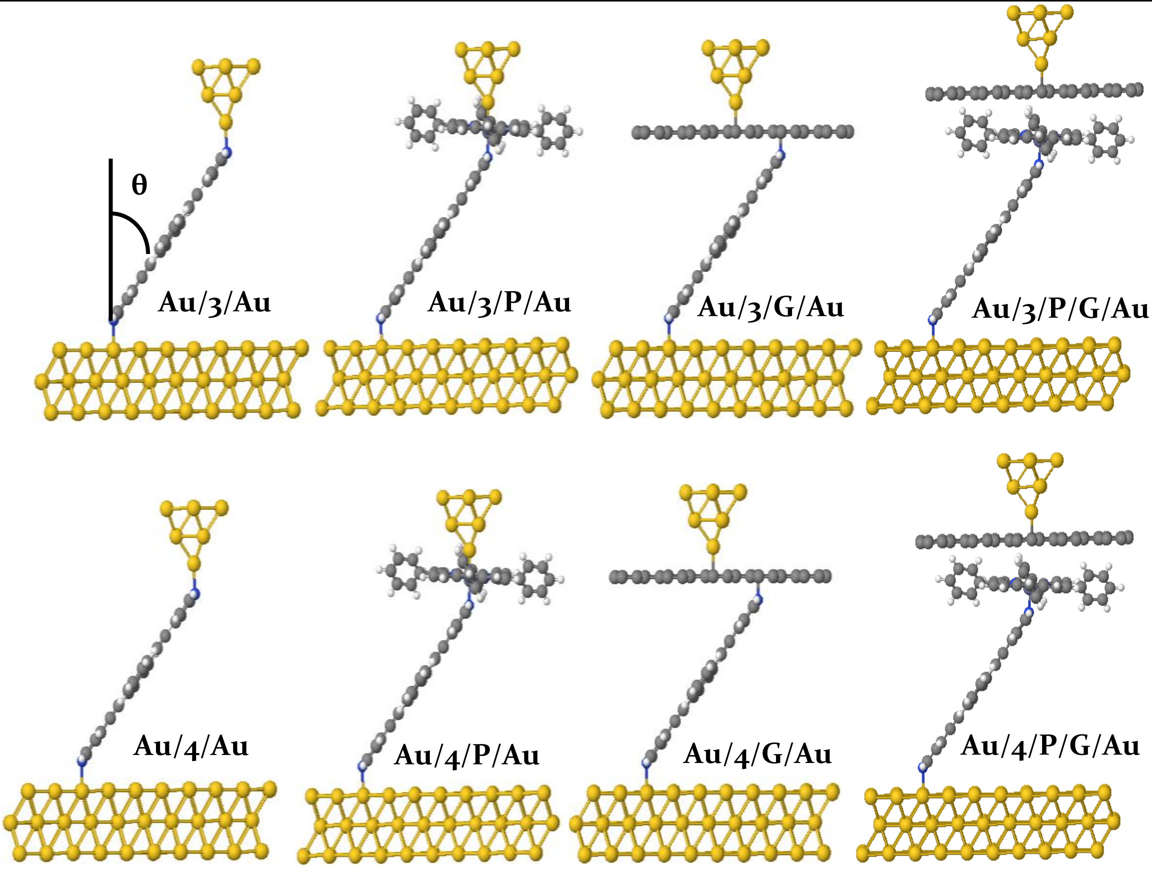

Figure S48: Optimised structures of the eight junctions, demonstrating the tilt-angle of the anthracene (side-view).

2.5 Transport calculations

The transmission coefficient curves *T(E)*, obtained using the Gollum transport code, were calculated for the eight junctions based on the tilt angles in Table S2 (different curves of the same colour correspond to different title angles and the yellow line is the average), as shown in the right panels of Figs. S49-52. Although the LUMO resonance is predicted to be pinned near the Fermi Level of the electrodes for these eight systems, previous comparisons between theory and experiment suggest that better agreement is obtained when the Fermi level is closer to the mid gap (see black-dashed lines in the right panels of Figs. S49-52).

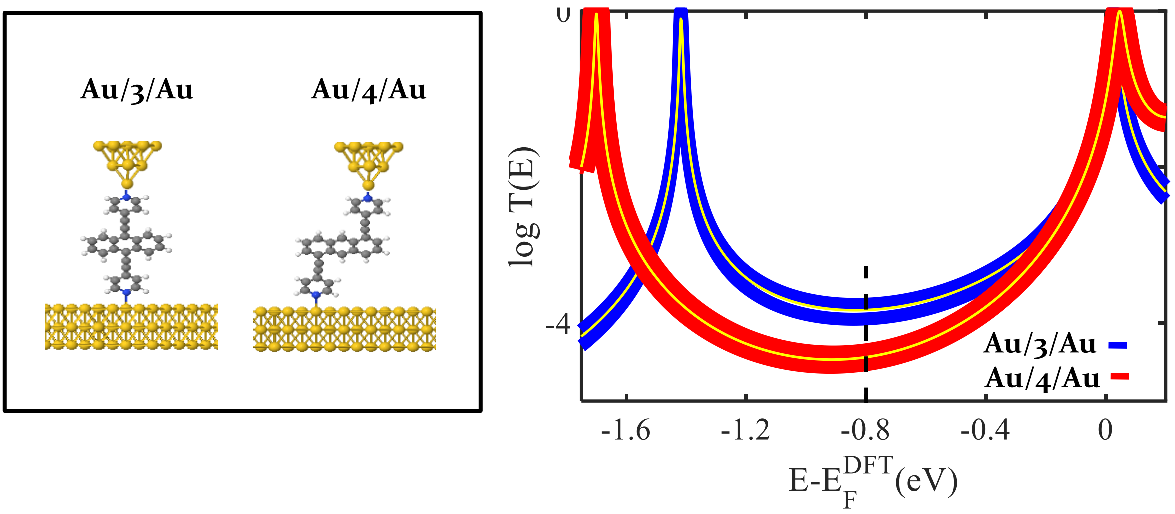

Figure S49: (Left panel): Schematic illustrations of molecular junctions for **Au/3/Au** and **Au/4/Au**. (Right panel): Zero bias transmission coefficient *T(E)* of **Au/3/Au** (blue solid-line) and **Au/4/Au** (red solid-line), against electron energy E. For Au/3/Au and Au/4/Au, results are presented for the tilt angles in ranges shown in Table S2. Different curves of the same colour correspond to different tilt angles. The yellow line is the average of four curves.

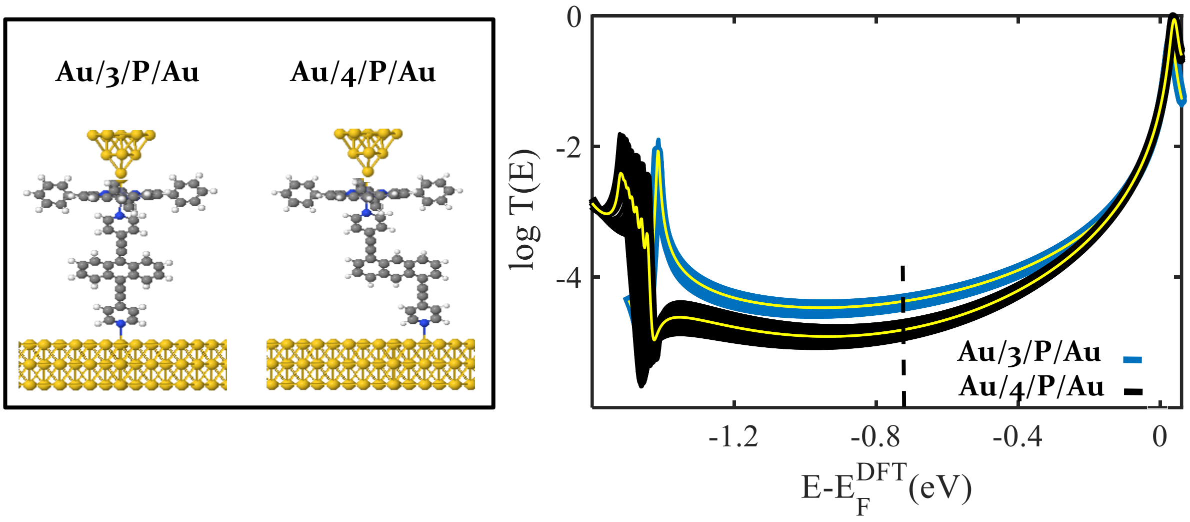

Figure S50: (Left panel): Schematic illustrations of molecular junctions for **Au/3/P/Au** and **Au/4/P/Au**. (Right panel): Zero bias transmission coefficient *T(E)* of **Au/3/P/Au** (light-blue solid line) and **Au/4/P/Au** (black solid-line), against electron energy E. For **Au/3/P/Au** and **Au/4/P/Au**, results are presented for the tilt angles in ranges shown in Table S2. Different curves of the same colour correspond to different tilt angles. The yellow line is the average of four curves.

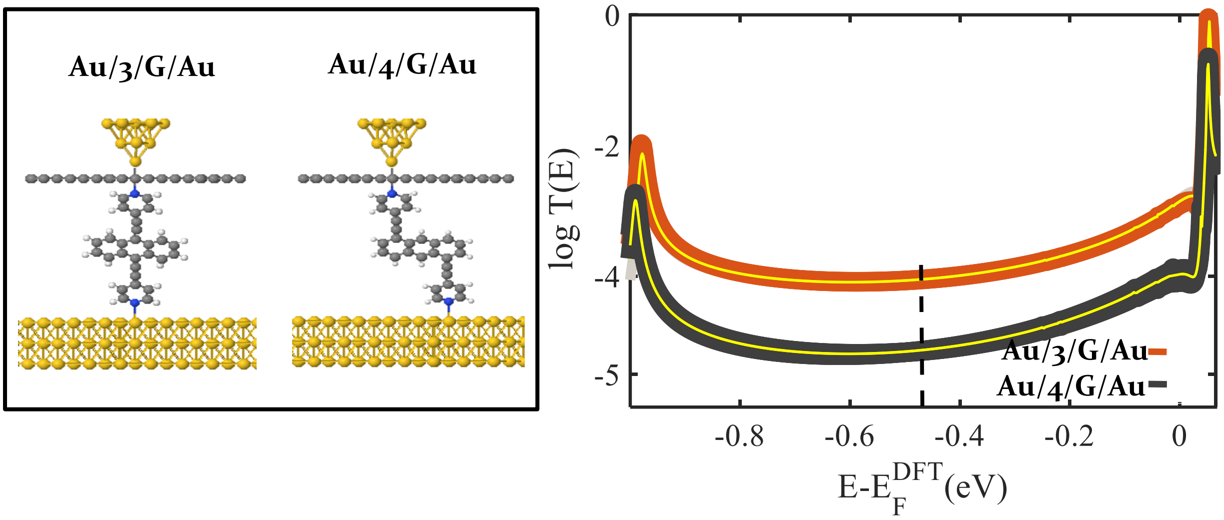

Figure S51: (Left panel): Schematic illustrations of molecular junctions for **Au/3/G/Au** and **Au/4/G/Au**. (Right panel): Zero bias transmission coefficient *T(E)* of molecules **Au/3/G/Au** (orange solid-line) and **Au/4/G/Au** (grey solid-line), against electron energy E. For **Au/3/G/Au** and **Au/4/G/Au**, results are presented for the tilt angles in ranges shown in Table S2. Different curves of the same colour correspond to different tilt angles. The yellow line is the average of four curves.

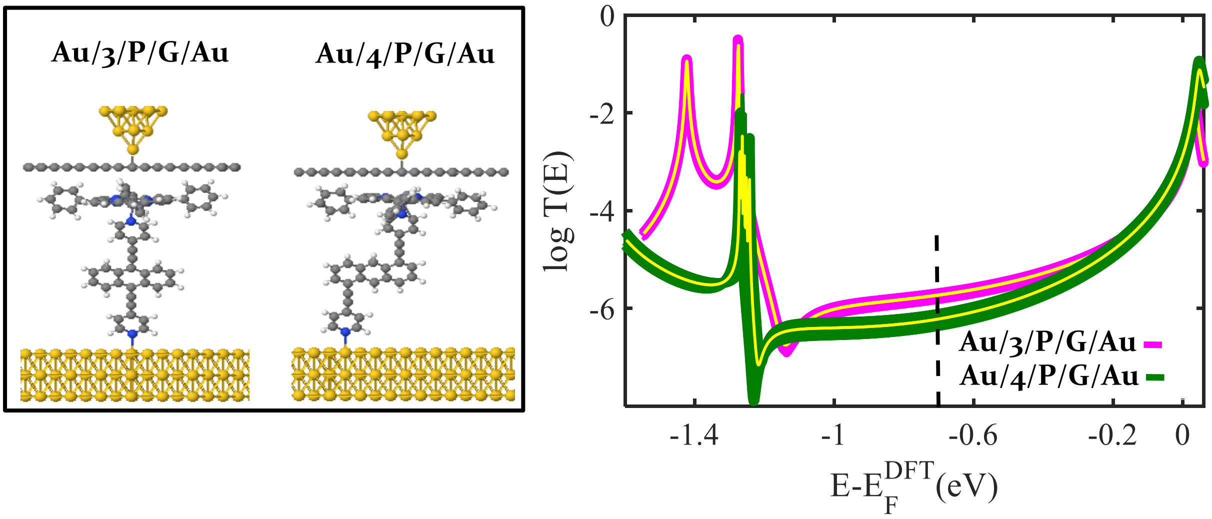

Figure S52: (Left panel): Schematic illustrations of molecular junctions for **Au/3/P/G/Au** and **Au/4/P/G/Au**. (Right panel): Zero bias transmission coefficient *T(E)* of molecules **Au/3/P/G/Au** (pink solid-line) and **Au/4/P/G/Au** (green solid-line), against electron energy E. For **Au/3/P/G/Au** and **Au/4/P/G/Au**, results are presented for the tilt angles in ranges shown in Table S2 Different curves of the same colour correspond to different title angles and the yellow line is the average of four curves.

2.6 Seebeck coefficient

After computing the electronic transmission coefficient for the eight molecules, we now compute their Seebeck coefficients S. To this end, it is useful to introduce the non-normalised probability distribution $\boldsymbol{P(E)}$ defined by;

$$P(E) = -\mathcal{T}(E)\frac{df(E)}{dE} \qquad \text{(S2)}$$

Where $\boldsymbol{f(E)}$ is the Fermi function and $\boldsymbol{\mathcal{T}(E)}$ are the transmission coefficients and whose moments $\boldsymbol{L_i}$ are denoted as follows;

$$L_i = \int dEP(E)(E - E_F)^i \qquad \text{(S3)}$$

Where $\boldsymbol{E_F}$ is the Fermi energy. The Seebeck coefficient, $\boldsymbol{S}$ and electrical conductance $\boldsymbol{G}$ are then given by;

$$S(T) = -\frac{1}{|e|T}\frac{L_1}{L_0} \qquad \text{(S4)}$$

$$G = \frac{2e^2}{h}L_0 \qquad \text{(S5)}$$

Where e is the electronic charge. Supplementary Figures S53-56 show the Seebeck coefficient $\boldsymbol{S}$ evaluated at room temperature for different energy range $E_F - E_F^{DFT}$.

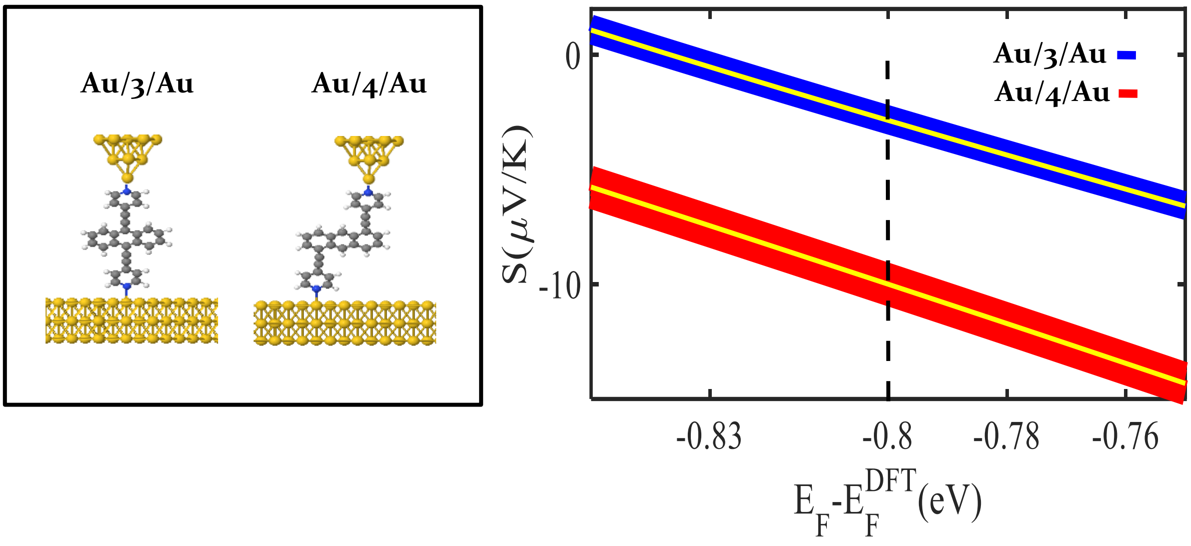

Figure S53: (Left panel): Schematic illustrations of molecular junctions for **Au/3/Au** and **Au/4/Au**. (Right panel): Seebeck coefficient S of **Au/3/Au** (blue solid-line) and **Au/4/Au** (red solid-line), against electron energy E. For **Au/3/Au** and **Au/4/Au**, results are presented for the tilt angles in ranges shown in Table S2 Different curves of the same colour correspond to different title angles and the yellow line is the average of four curves.

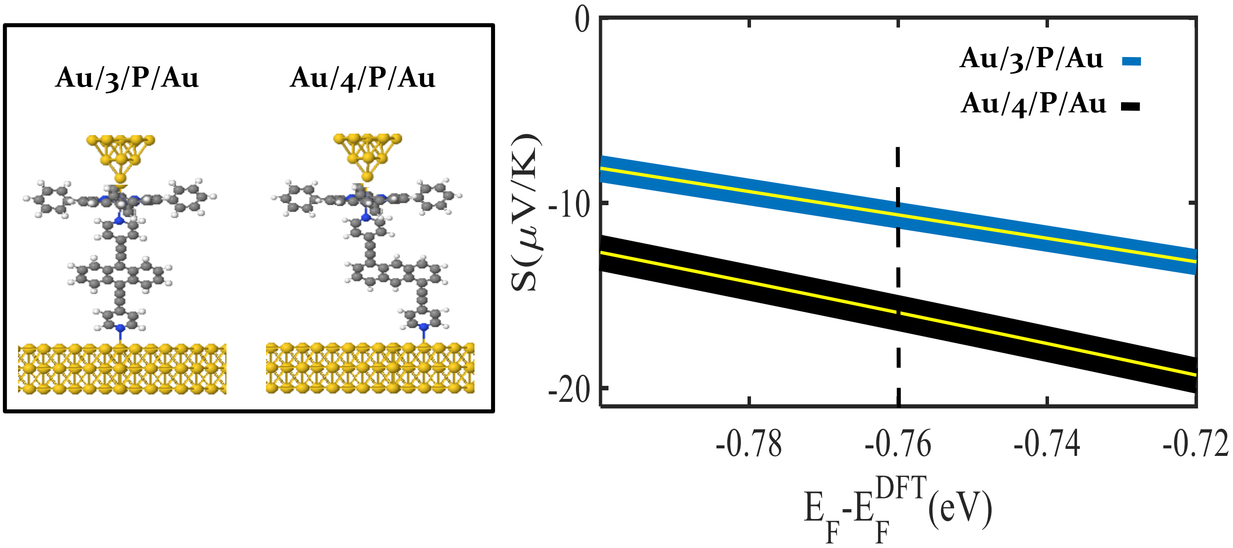

Figure S54: (Left panel): Schematic illustrations of molecular junctions for **Au/3/P/Au** and **Au/4/P/Au**. (Right panel): Seebeck coefficient S of **Au/3/P/Au** (light-blue solid-line) and **Au/4/P/Au** (black solid-line), against electron energy E. For **Au/3/P/Au** and **Au/4/P/Au**, results are presented for the tilt angles in ranges shown in Table S2 Different curves of the same colour correspond to different title angles and the yellow line is the average of four curves.

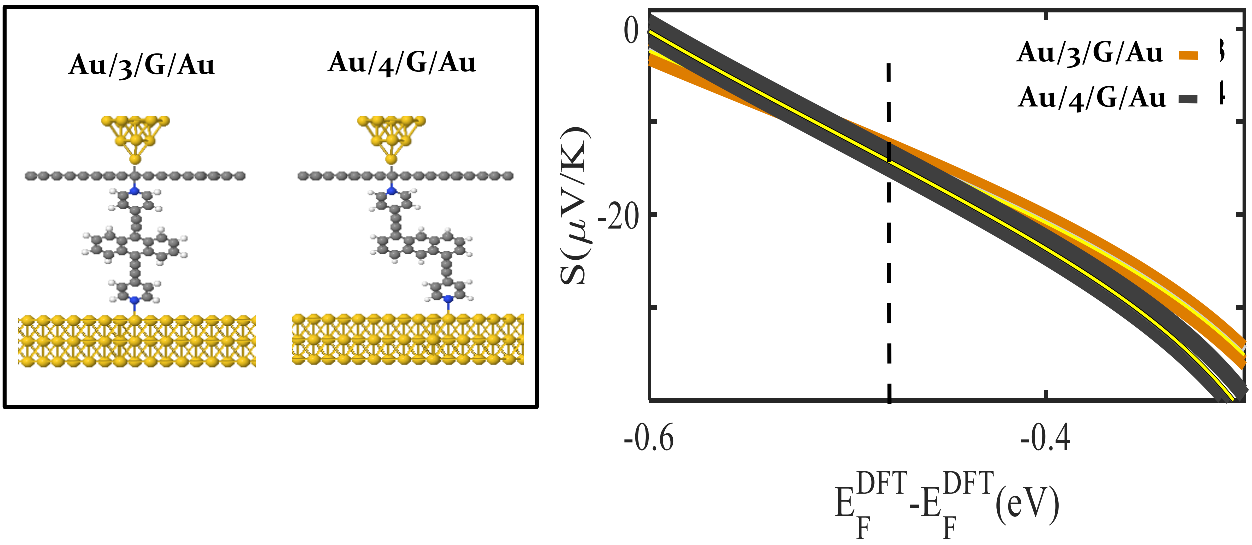

Figure S55: (Left panel): Schematic illustrations of molecular junctions for **Au/3/G/Au** and **Au/4/G/Au**. (Right panel): Seebeck coefficient S of **Au/3/G/Au** (orange solid-line) and **Au/4/G/Au** (grey solid-line), against electron energy E. For **Au/3/G/Au** and **Au/4/G/Au**, results are presented for the tilt angles in ranges shown in Table S2 Different curves of the same colour correspond to different title angles and the yellow line is the average of four curves.

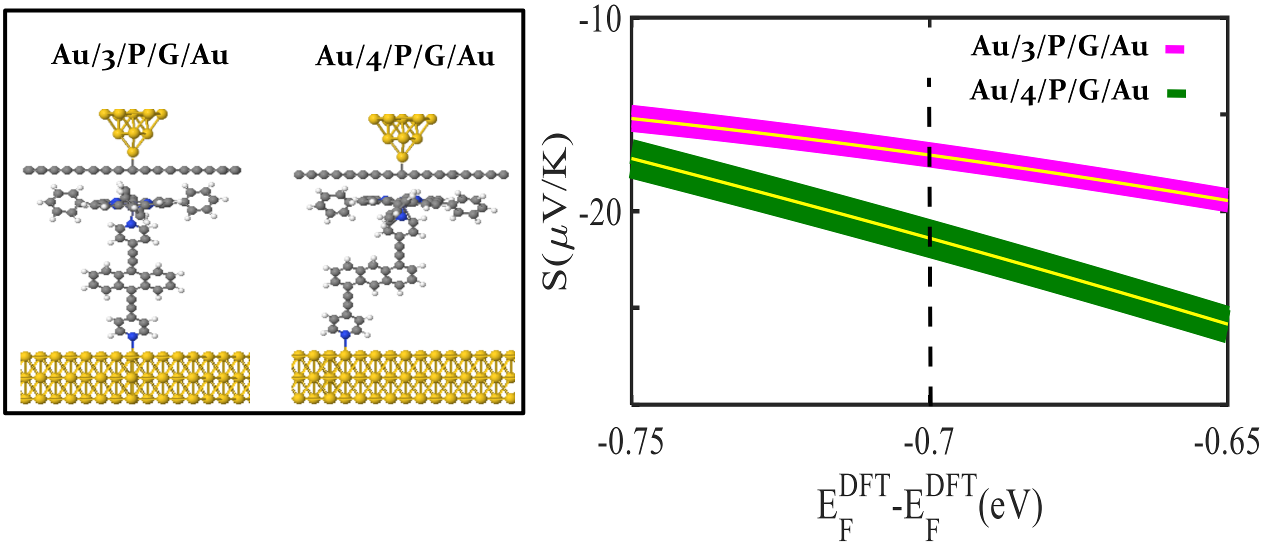

Figure S56: (Left panel): Schematic illustrations of molecular junctions for **Au/3/P/G/Au** and **Au/4/P/G/Au**. (Right panel): Seebeck coefficient S of molecules **Au/3/P/G/Au** (pink solid-line) and **Au/4/P/G/Au** (green solid-line), against electron energy E. For **Au/3/P/G/Au** and **Au/4/P/G/Au**, results are presented for the tilt angles in ranges shown in Table S2 Different curves of the same colour correspond to different title angles and the yellow line is the average of the four curves.

3. Formation and Thermoelectric Characterisation of SAMs

3.1 Au preparation

Template stripped gold (Au^{TS}) was prepared by a modified method of Whitesides and Pinkhassik.[14,15] A Si wafer (5 mm x 5 mm) was cleaned using an ultrasonic bath with acetone, methanol and isopropanol (IPA), then cleaned with oxygen plasma for 5 minutes. The cleaned wafer was glued onto the top surface of a thermal evaporated gold chip (100 nm thickness) with Epotek 353nd epoxy adhesive. The adhesive was cured for 40 minutes at 150 °C, then cooled down to room temperature. The Si contact with gold without epoxy and adhesive was carefully removed using a sharp blade and leaving an atomically-flat Au surface.

The prepared gold was scanned by AFM for 3-5 random spots for quality tests. For all cases, only the substrates with roughness below 0.2 nm were used for SAMs growth.

3.2 SAMs growth

Growth of anthracene-based SAMs: 1mM solution of molecule (1-4) was dissolved in toluene (>99.5%, Sigma Aldrich), with 10 minute nitrogen bubbling for oxygenation. The prepared Au^{TS} was immersed into the solution, and incubated for 24 hours under nitrogen atmosphere, and the molecules will spontaneously form an organic thin film on Au surface due to the specialized anchor and the intermolecular interaction. The SAMs modified Au was rinsed by toluene and IPA several times to wash-off the physisorped molecules. The sample after rinsing was blown with nitrogen for drying and incubated in vacuum oven (10^{-2} mbar) overnight at 35°C for solvent evaporation.

Zn-TPP deposition: The SAMs modified Au substrates were immersed in 100 μM **Zn-TPP** solutions dissolved in toluene for **Zn-TPP** deposition. Among different deposition time, we found that 20 minutes of deposition gives a **Zn-TPP** layer with thickness relatively comparable with a single **Zn-TPP** lying on a flat surface.

The SAMs growth procedure on QCM substrate was the same as the growth on Au^{TS}. The resonance frequency of the substrate before and after SAMs growth was recorded by the QCM instrument (OpenQCM), and the shift in frequency, Δf, implies the amount of molecule adsorbed on the substrate surface, through use of the Sauerbrey equation (S7).[16]

$$\mathbf{n} = \frac{-\Delta \boldsymbol{f} \times \boldsymbol{A} \times \boldsymbol{k} \times \boldsymbol{N_A}}{\boldsymbol{M_w}} \tag{S6}$$

$$\boldsymbol{k} = \frac{\sqrt{\mu * \boldsymbol{\rho}}}{2 * \boldsymbol{f_0^2}} \tag{S7}$$

Where $\boldsymbol{n}$ the amount of molecule adsorbed on Au surface, $\boldsymbol{A}$ the electrode area, $\boldsymbol{N_A}$ the Avogadro's number, $\boldsymbol{M_w}$ the molecular weight, $\boldsymbol{\mu}$ the shear modulus of quartz, $\boldsymbol{\rho}$ the density of quartz, $\boldsymbol{f_o}$ the initial frequency.

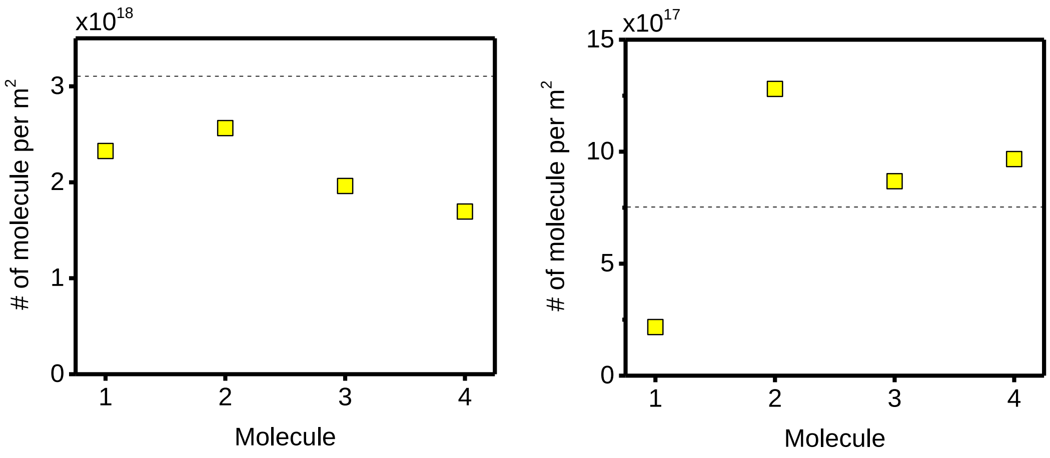

Figure S57: Amount of molecule per m[2] calculated from QCM result (left). SAMs of molecules **1-4** (right) after addition of **Zn-TPP** on the top surface of their SAM's. Dashed line: the expected value of a closely packed monolayer.

3.3 SAM Topography

AFM characterization: peak force AFM (PF-AFM, Brucker, 500 pN force at 2 kHz) was used to characterize the surface of the Au substrate, before and after SAMs growth. Our previous publication demonstrated that these anthracene molecules forms well-ordered SAMs.[1,2]

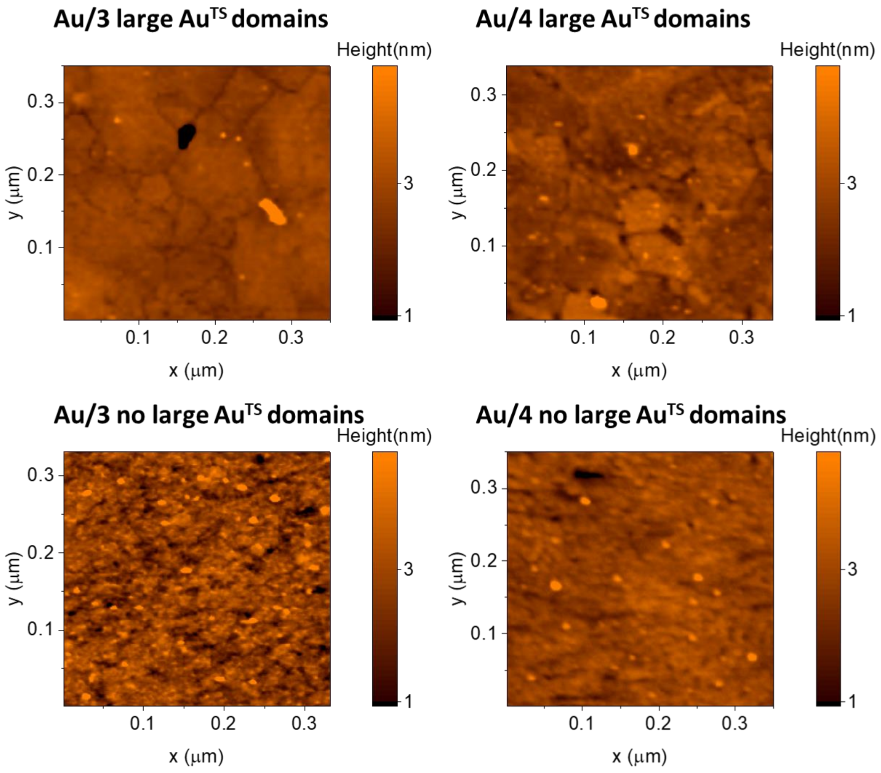

Figure S58: Atypical images to illustrate the growth of SAMs of both **3** and **4**.

The topography of the SAMs of **1-4** after **Zn-TPP** deposition is shown below:

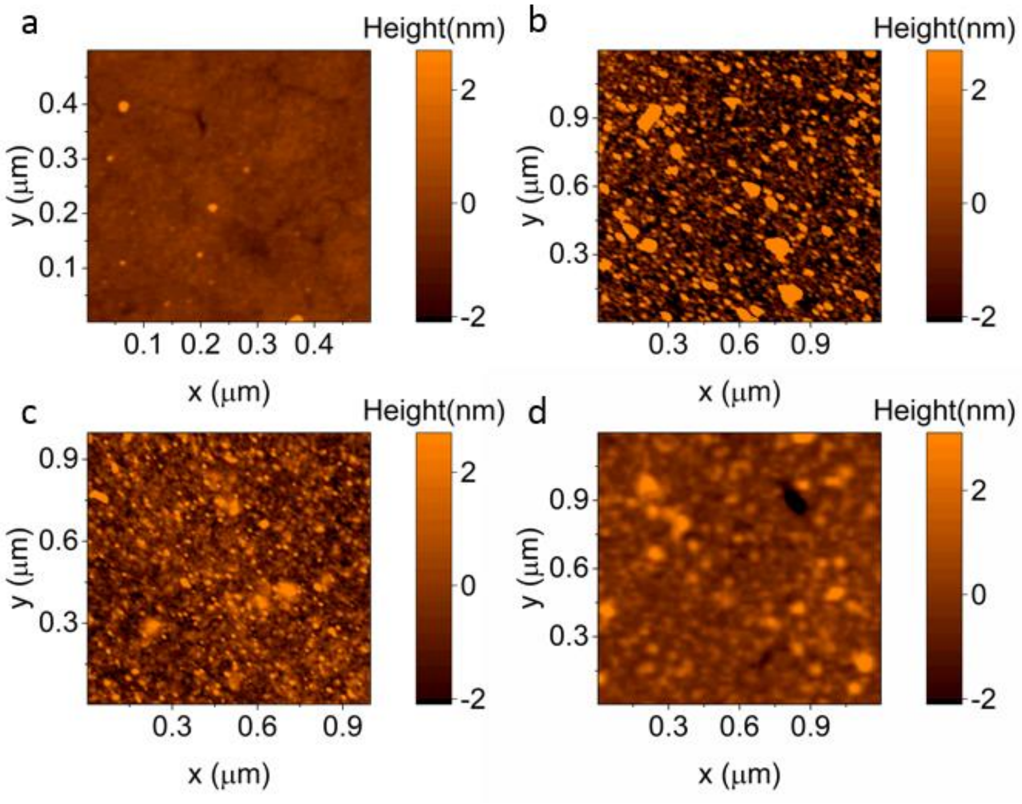

Figure S59: Topographical images of (a) **Au/SAM1**, (b) **Au/SAM2**, (c) **Au/SAM3** and (d) **Au/SAM4** after **Zn-TPP** layer deposition.

The nano-scratching was performed in contact mode at high set force (F = 15 - 40 nN) using a soft probe (Multi-75-G, k = 3 N/m) to 'sweep away' the molecular film from a defined area. The topography of each sample, after scratching, was again characterized in peak force mode, where the scratched window is easily observed. Nano-scratching was also conducted on a bare gold sample under the same conditions to ensure no gold is scratched away in the force range that we used. The height difference between the 'scratched' part and 'un-scratched' part indicates the thickness of SAMs.

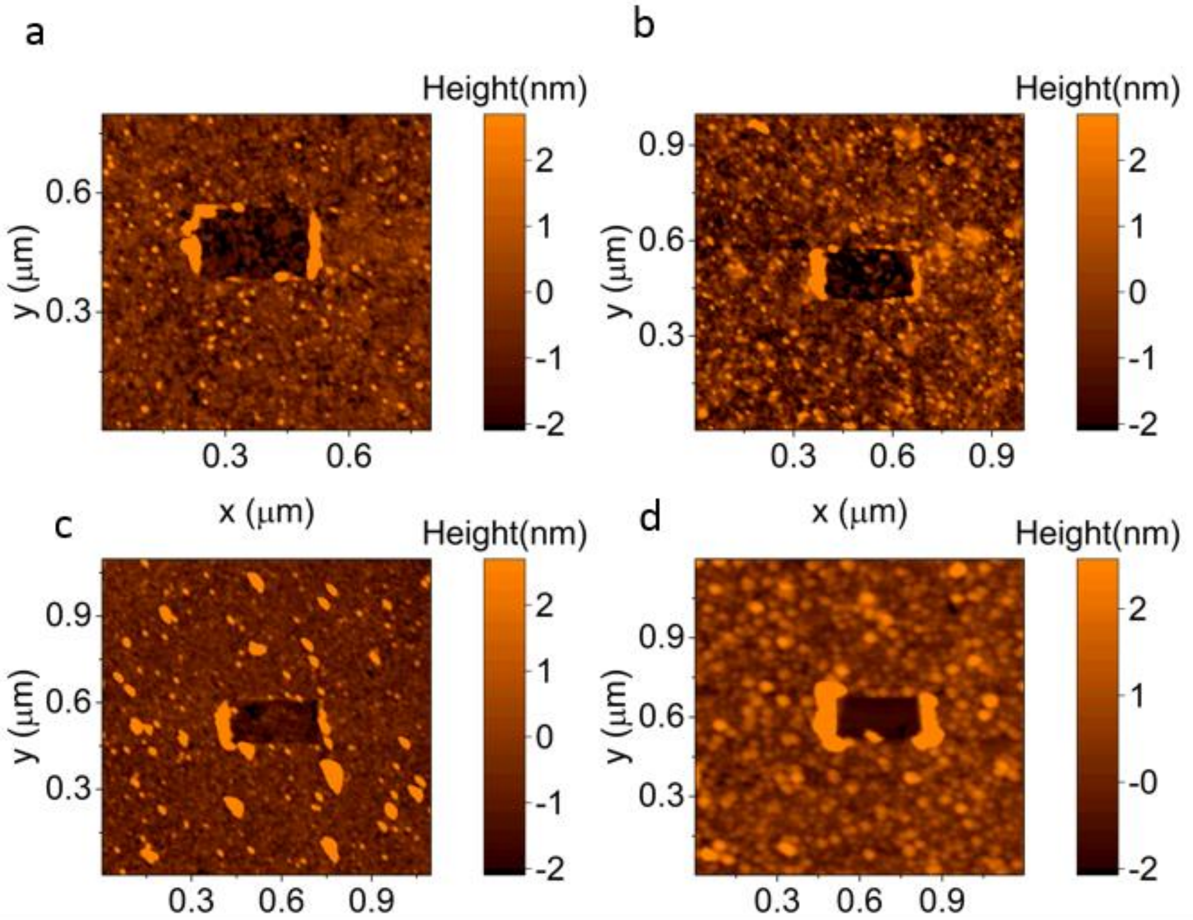

Figure S60: Nanoscratching image of SAMs of molecule **3** before (a) and after (b) **Zn-TPP** deposition, and the nanoscratching image of molecule **2** and **4** after **Zn-TPP** deposition (c) and (d) respectively.

With a combination of QCM, AFM imaging and Nano scratching, we conclude that under the same deposition conditions (100 μM for 20 min), the amount of **Zn-TPP** deposited on each SAM was as follows; **SAM1** < **SAM3** ≈ **SAM4** < **SAM2**. For **SAM2**, topological image's suggested that there were quite a number of large clusters on the sample surface instead of continuous film. However, **SAM3** and **SAM4** formed well defined **Zn-TPP** layer with reasonable thickness, which was useful for further characterization.

3.4 Electric and thermoelectric characterization

A modified AFM system was used for electric and thermoelectric characterization. The electrical transport properties of the SAMs were characterized by a cAFM system. The cAFM setup is based on a Multi-mode 8 AFM system (Bruker Nano Surfaces). The bottom gold substrate was used as the source, and a Pt/Cr coated probe (Multi75 E, BugetSensors) was used as the drain. The force between probe and molecule was controlled at 2 nN, as this force is strong enough for the probe to penetrate through the water layer on the sample surface but not too strong so as to destroy the molecular thin film. The triangular shape AC bias was added between the source and drain by a voltage generator (Aglient 33500B), the source to drain current was acquired by a current pre-amplifier (SR570, Stanford Research Systems) providing current-to-voltage conversion. The I-V characteristics were obtained by Nanoscope 8 controller simultaneously collecting drive bias and current with subsequent correlation of these values at each time point.

The conductance of the molecule was calculated by the derivative of the IV curve, dI/dV, at near 0 bias region (-10 mV to 10 mV), and the amount of molecules getting contact with the probe was estimated utilising the RKJ model (S8).[17,18]

$$\boldsymbol{r} = (\boldsymbol{F} \times \boldsymbol{R} \times \frac{\mathbf{1}}{\boldsymbol{Y}})^{\frac{1}{3}} \qquad \text{(S8)}$$

$$\frac{\mathbf{1}}{\boldsymbol{Y}} = \frac{\mathbf{3}}{\mathbf{4}} \times (\frac{\mathbf{1} - \boldsymbol{v}_{\mathbf{1}}^{\mathbf{2}}}{\boldsymbol{E}_{\mathbf{1}}} + \frac{\mathbf{1} - \boldsymbol{v}_{\mathbf{2}}^{\mathbf{2}}}{\boldsymbol{E}_{\mathbf{2}}}) \qquad \text{(S9)}$$

Where $\boldsymbol{r}$ is the contact radius, $\boldsymbol{F}$ is the loading force from probe to sample, $\boldsymbol{R}$ is the radius of the probe, $\boldsymbol{v_1}$ and $\boldsymbol{v_2}$ the Poisson ratio of the material, $\boldsymbol{E_1}$ and $\boldsymbol{E_2}$ are the Young's Modulus for probe and SAMs respectively. The radius of the probe was obtained from SEM image and estimated to be 25 nm. The Young's modulus was obtained from AFM in peakforce mode, which was about 2 GPa for the SAMs. Other parameters were obtained from literature working on similar systems. The graphene was coated onto the conductive probe by LB film.

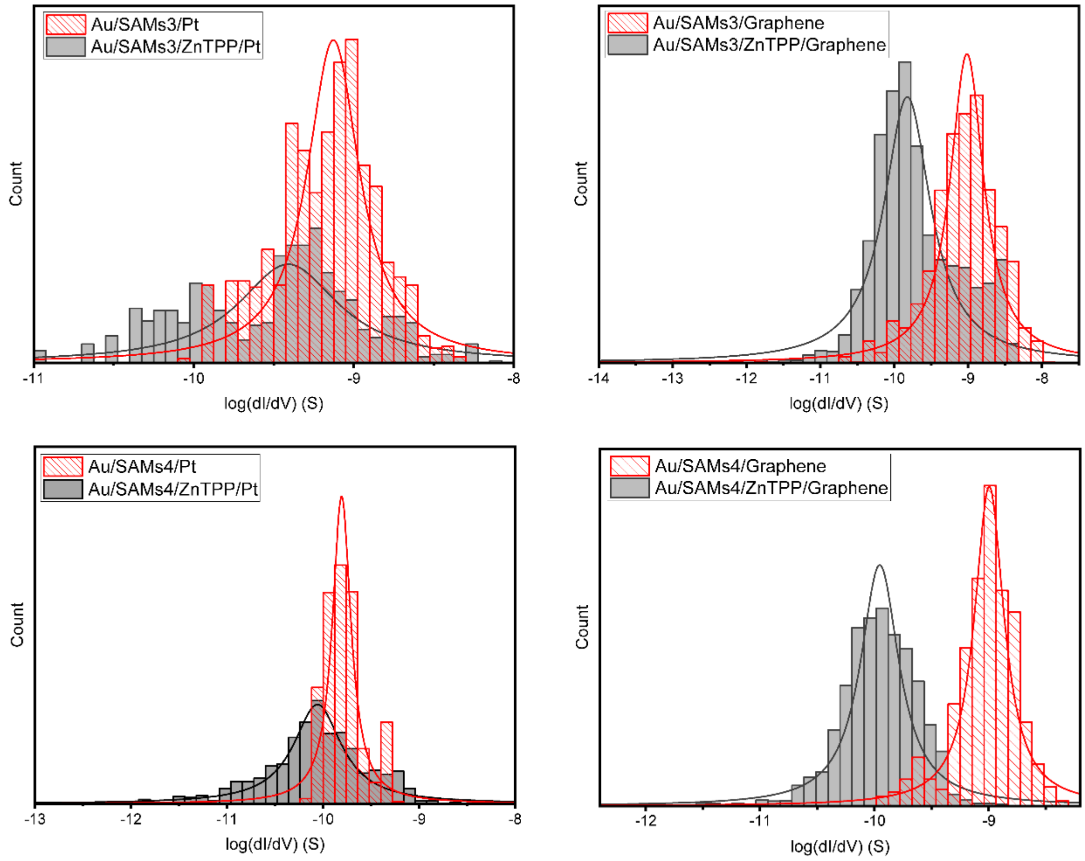

Figure S61: Conductance distribution of the 8 different junctions mentioned in the main text.

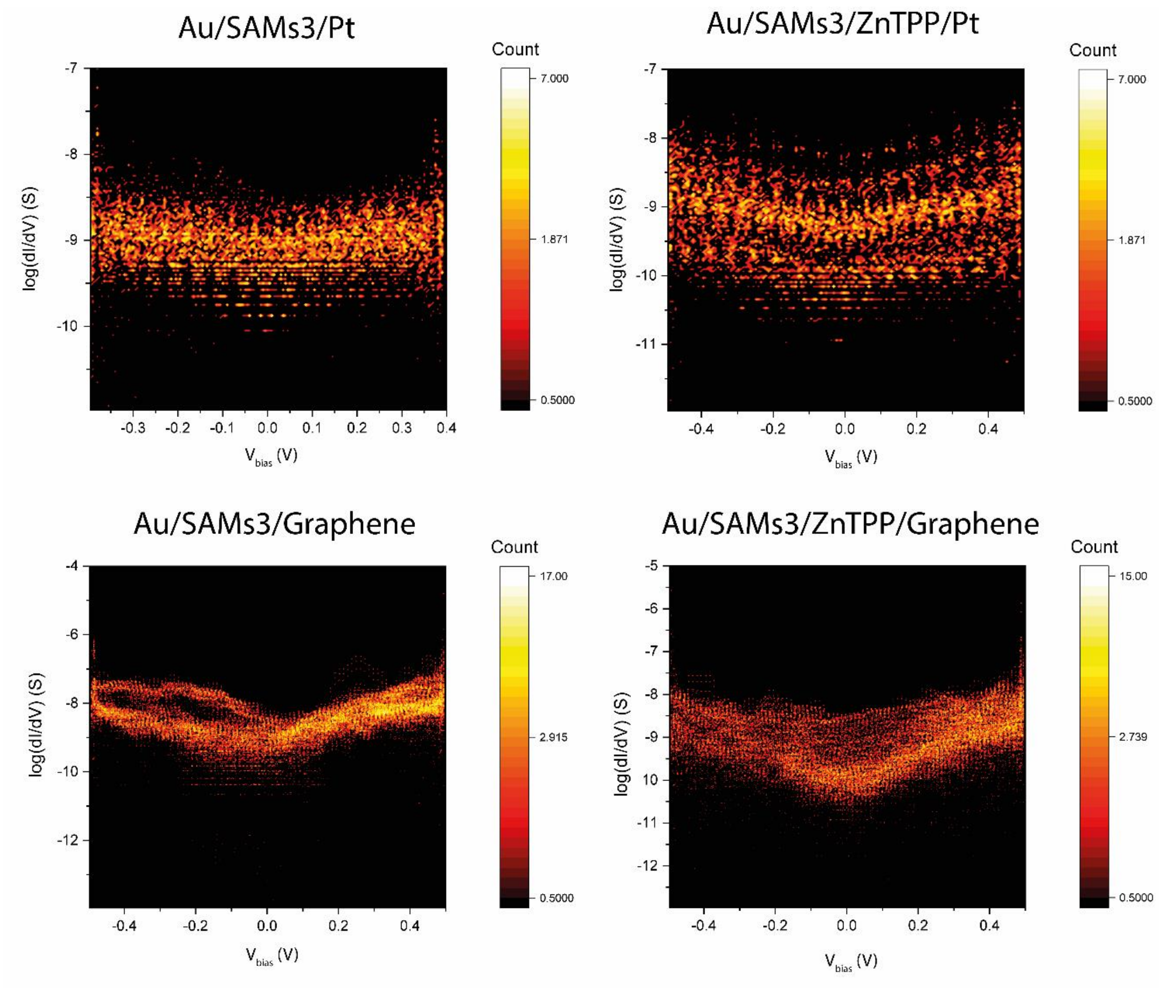

Figure S62: Statistical IV graphs of **SAM3** based molecular junctions **Au/SAM3/Pt** (left) and **Au/SAM3/P/Pt** (right)

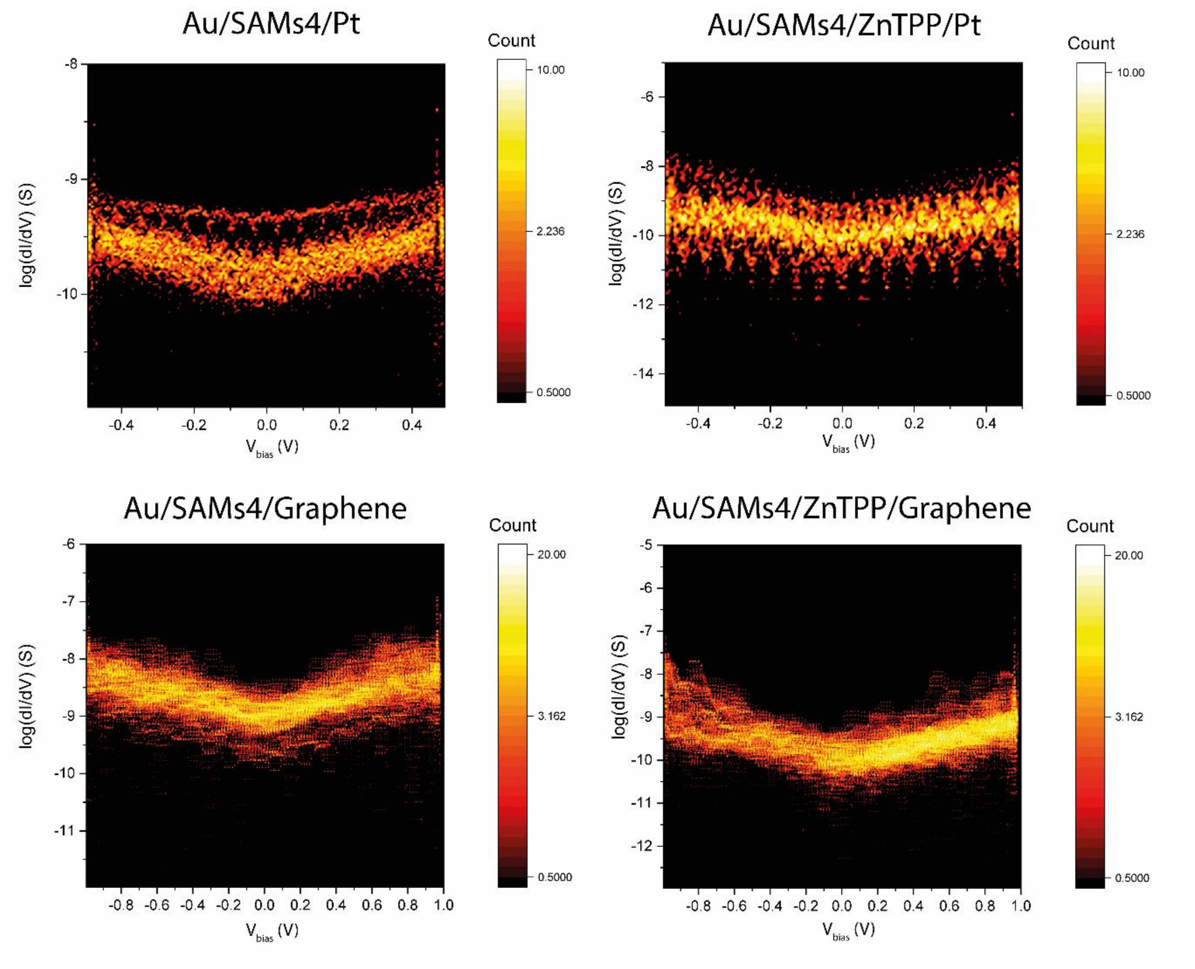

Figure S63: Statistical IV graphs of all **Au/4** based molecular junctions.

The Seebeck coefficients of SAMs were obtained by a ThEFM, modified from the cAFM system used for electrical transport measurement. A Peltier stage driven by a voltage generator (Agilent 33500B, with auxiliary amplifier to provide sufficient drive current) and was used for heating up and cooling the sample, therefore providing a temperature difference between sample and probe. The probe used was a commercially available Pt coated probe coated with additional layers of 5 nm Cr and 30 nm Au to enlarge the contact area for voltage stability. The graphene was coated on the top of the probe with same method as described in the cAFM section.

The sample temperature was measured by a Type T thermal couple, and the probe temperature can be assumed to be equal to ambient temperature as is in the case of low thermal conductivity sample's (organic film) as shown elsewhere.[2] The thermal voltage between sample and probe was amplified by a high impedance differential pre-amplifier (SR560, Stanford Research Systems), and recorded by a computer.

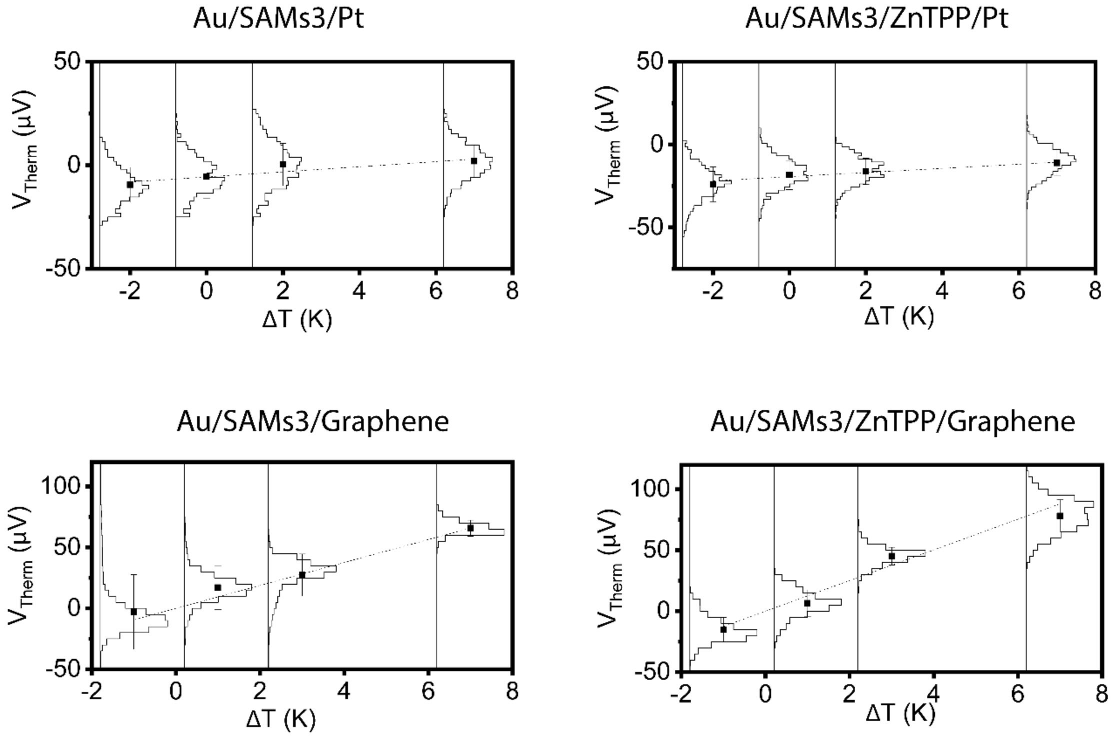

Figure S64: Thermal voltage vs. temperature different, for **Au/3** junctions.

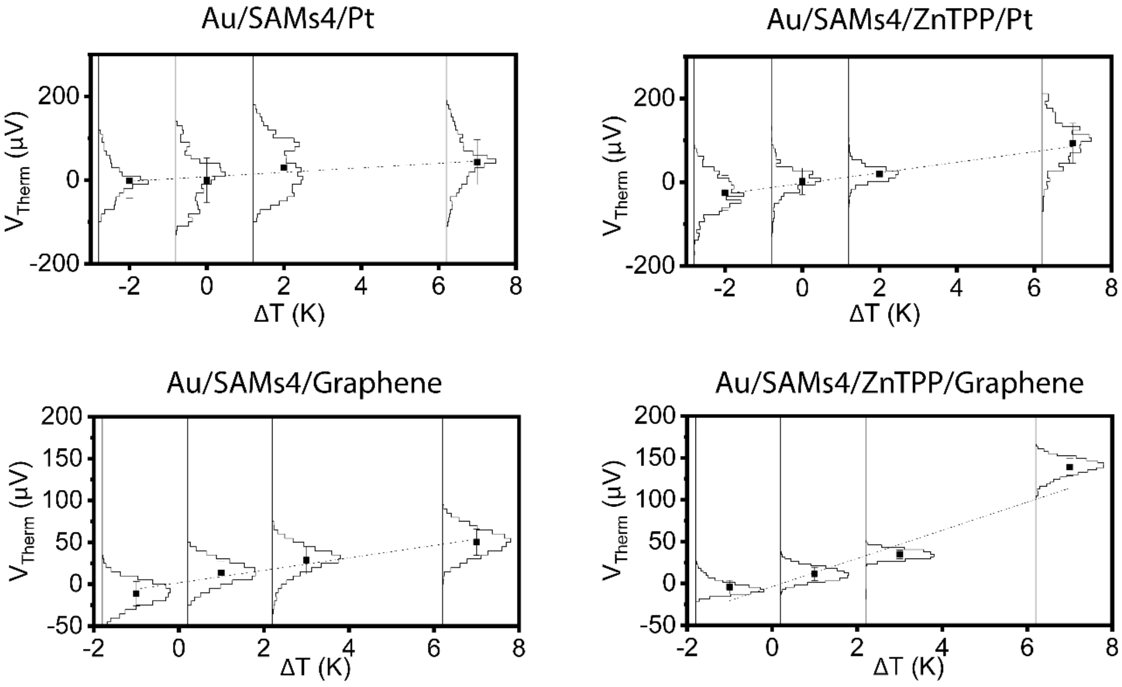

Figure S65: Thermal voltage vs. temperature different, for **Au/4** junctions.

Table S3: Summary of measured and theoretical electric and thermoelectric properties of different junction's.

Junction	Exp. log(G(S))	std	Theo. log(G(S))	Exp. S (μV/K)	std	Theo. S (μV/K)
**Au/SAM3/Pt**	-9.16	0.3	-8.01	-1.22	0.3	-2.4
**Au/SAM4/Pt**	-9.70	0.2	-8.14	-5.35	1.3	-9.2
**Au/SAM3/P/Pt**	-9.49	0.5	-8.20	-1.25	0.2	-10.4
**Au/SAM4/P/Pt**	-10.10	0.5	-9.3	-12.58	1.3	-9.8
**Au/SAM3/G/Pt**	-8.76	0.2	-8.8	-8.8	0.5	-9.4
**Au/SAM4/G/Pt**	-9.03	0.3	-10.05	-7.4	1.6	-14.7
**Au/SAM3/P/G/Pt**	-9.85	0.4	-10.0	-12.9	2.0	-15.1
**Au/SAM4/P/G/Pt**	-9.90	0.3	-10.3	-16.8	2.2	-21.5

4. X-Ray Photoelectron Spectroscopy

4.1 XPS Characterisation of Au/1, Au/1/P, Au/2 and Au/2/P

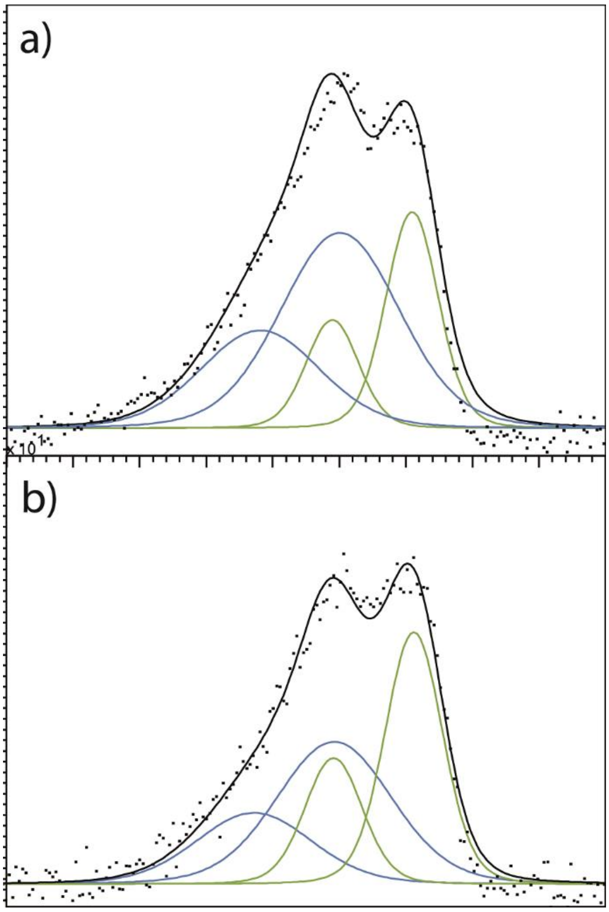

Figure S66: XPS characterisation of the S 2p region in a) **Au/1** and b) **Au/1/P**.

XPS was performed on self-assembled monolayers of **Au/2** with and without **Zn-TPP**. Spectra for **Au/2** in the N 1s region shows two peaks at 399.8 eV and 401.68 eV, representing bound and unbound pyridyl units (Fig. S67 (a)). After **Zn-TPP** deposition, bound and unbound nitrogen peaks were still present at similar binding energies, 400.1 eV and 402.12 eV, however an additional nitrogen appears due to the binding of nitrogen within porphyrin showing three peaks at 398.4 eV, as shown in Fig. S67 b. Similar to **Au/3** and **Au/4**, a peak is expected from the anthracene **Zn-TPP** interaction and cannot be seen under the binding energy from Au-N interactions. We note that the unbound peak following **Zn-TPP** deposition has increased which we attribute to toluene solvent removing some of the bound anthracene layer.

As expected from characterisation of the sulphur region two sets of doublets were observed representing bound and unbound thiomethyl. (Peaks were fitted using standard methods with two Guassian-Lorentzian S 2p doublets with a 2:1 area ratio and splitting 1.2 eV). Identical doublets were found at 161.8 eV and 164.6 eV, as shown in Fig. S67 (c-d). In the **Au/2** system, we note that there is a mixture of both bound and unbound nitrogen and thiol units suggesting that **Au/2** forms a mixed layer. Fig S67 (e) shows spectra recorded in the zinc region to confirm presence of **Zn-TPP**. A peak was found at 1021.7 eV, similar to the **Au/3** and **Au/4** systems.

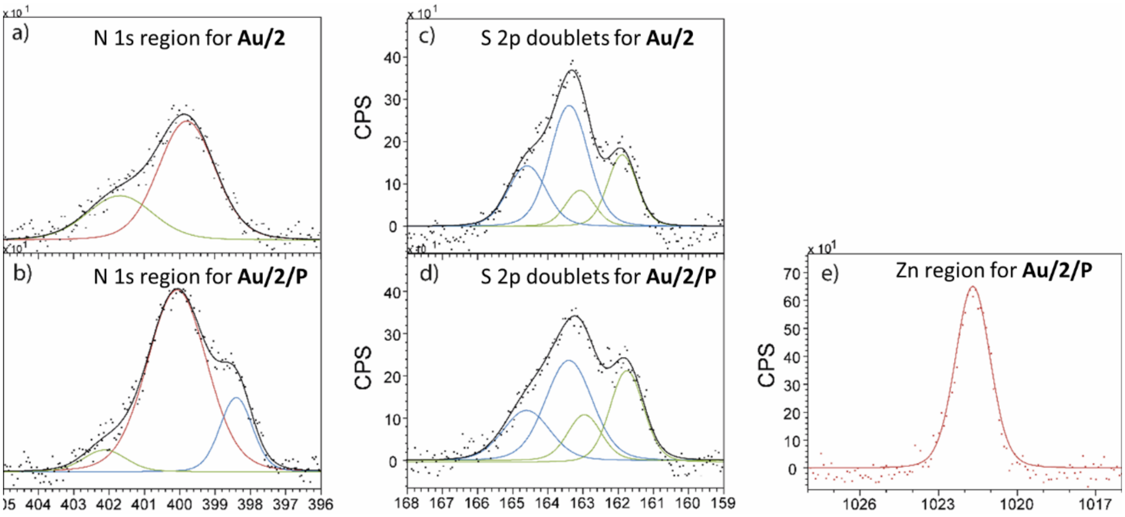

Figure S67: XPS characterisation of surface binding for **Au/2** and **Au/2/P** layers.

Broad scanning indicated the presence of a small amount of iodide species in **Au/2** (6.7%) and **Au/3** (12.6%) believed to originate from the copper-iodide co-catalyst used in the preparation of these systems, but is believed to play no detrimental role in the SAMs assembly or performance.

5. References

1. A. Ismael, X. Wang, T. L. R. Bennett, L. A. Wilkinson, B. J. Robinson, N. J. Long, L. F. Cohen and C. J. Lambert, *Chem. Sci.*, 2020, **11**, 6836.
2. X. Wang, T. L. R. Bennett, A. Ismael, L. A. Wilkinson, J. Hamill, A. J. P. White, I. M. Grace, O. V. Kolosov, T. Albrecht, B. J. Robinson, L. F. Cohen and C. J. Lambert, *J. Am. Chem. Soc.*, 2020, **142**, 8555.
3. A. Lohr and T. M. Swager, *J. Mater. Chem.*, 2010, **20**, 8107.
4. E. Artacho, E. Anglada, O. Diéguez, J. D. Gale, A. García, J. Junquera, R. M. Martin, P. Ordejón, J. M. Pruneda, D. Sánchez-Portal and J. M. Soler, *J. Phys. Condens. Matter*, 2008, **20**, 064208.
5. J. M. Soler, E. Artacho, J. D. Gale, A. García, J. Junquera, P. Ordejón and D. Sánchez-Portal, *J. Phys. Condens. Matter*, 2002, **14**, 2745.
6. I. L. Herrer, A. K. Ismael, D. C. Milán, A. Vezzoli, S. Martín, A. González-Orive, I. Grace, C. J. Lambert, J. L. Serrano, R. J. Nichols and P. Cea, *J. Phys. Chem. Lett.*, 2018, **9**, 5364.
7. A. K. Ismael, K. Wang, A. Vezzoli, M. K. Al-Khaykanee, H. E. Gallagher, I. M. Grace, C. J. Lambert, B. Xu, R. J. Nichols and S. J. Higgins, *Angew. Chem. Int. Ed.*, 2017, **56**, 15378.
8. A. Markin, A. K. Ismael, R. J. Davidson, D. C. Milan, R. J. Nichols, S. J. Higgins, C. J. Lambert, Y. T. Hsu, D. S. Yufit and A. Beeby, *J. Phys. Chem. C*, 2020, **124**, 6479.
9. L. Herrer, A. Ismael, S. Martín, D. C. Milan, J. L. Serrano, R. J. Nichols, C. Lambert and P. Cea, *Nanoscale,* 2019, **11**, 15871.
10. D. C. Milan, M. Krempe, A. K. Ismael, L. D. Movsisyan, M. Franz, I. Grace, R. J. Brooke, W. Schwarzacher, S. J. Higgins, H. L. Anderson, C. J. Lambert, R. R. Tykwinski and R. J. Nichols, *Nanoscale*, 2017, **9**, 355.
11. R. J. Davidson, D. C. Milan, O. A. Al-Owaedi, A. K. Ismael, R. J. Nichols, S. J. Higgins, C. J. Lambert, D. S. Yufit and A. Beeby, *RSC Adv.* 2018, **8**, 23585.
12. A. K. Ismael and C. J. Lambert, *J. Mater. Chem. C*, 2019, **7**, 6578.
13. S. Naghibi, A. K. Ismael, A. Vezzoli, M. K. Al-Khaykanee, X. Zheng, I. M. Grace, D.Bethell, S. J. Higgins, C. J. Lambert and R. J. Nichols, *J. Phys. Chem. Lett.*, 2019, **10**, 6419.
14. E. A. Weiss, G. K. Kaufman, J. K. Kriebel, Z. Li, R. Schalek and G. M. Whitesides, *Langmuir*, 2007, **23**, 9686.
15. L. T. Banner, A. Richter and E. Pinkhassik, *Surf. Interface Anal.*, 2009, **41**, 49.
16. G. Sauerbrey, *Zeitschrift für Phys.* 1959, **155**, 206.
17. K. L. Johnson, K. Kendall and A. D. Roberts, *Proc. R. Soc. London. A. Math. Phys. Sci.*, 1971, **324**, 301.
18. N. A. Burnham, R. J. Colton and H. M. Pollock, *Phys. Rev. Lett.*, 1992, **69**, 144.
